# A Temporal Diversity Analysis of Brazilian Begomoviruses in Tomato Reveals a Decrease in Species Richness between 2003 and 2016

**DOI:** 10.3389/fpls.2020.01201

**Published:** 2020-08-06

**Authors:** Tadeu Araujo Souza, João Marcos Fagundes Silva, Tatsuya Nagata, Thaís Pereira Martins, Erich Yukio Tempel Nakasu, Alice Kazuko Inoue-Nagata

**Affiliations:** ^1^Department of Plant Pathology, University of Brasilia, Brasilia, Brazil; ^2^Laboratory of Virology, Embrapa Vegetables, Brasilia, Brazil; ^3^Department of Cell Biology, University of Brasilia, Brasilia, Brazil

**Keywords:** begomovirus, capulavirus, diversity, population, geminivirus, resistance gene, metagenomics

## Abstract

Understanding the molecular evolution and diversity changes of begomoviruses is crucial for predicting future outbreaks of the begomovirus disease in tomato crops. Thus, a molecular diversity study using high-throughput sequencing (HTS) was carried out on samples of infected tomato leaves collected between 2003 and 2016 from Central Brazil. DNA samples were subjected to rolling circle amplification and pooled in three batches, G1 (2003–2005, N = 107), G2 (2009–2011, N = 118), and G3 (2014–2016, N = 129) prior to HTS. Nineteen genome-sized geminivirus sequences were assembled, but only 17 were confirmed by PCR. In the G1 library, five begomoviruses and one capula-like virus were detected, but the number of identified viruses decreased to three begomoviruses in the G2 and G3 libraries. The bipartite begomovirus tomato severe rugose virus (ToSRV) and the monopartite tomato mottle leaf curl virus (ToMoLCV) were found to be the most prevalent begomoviruses in this survey. Our analyses revealed a significant increase in both relative abundance and genetic diversity of ToMoLCV from G1 to G3, and ToSRV from G1 to G2; however, both abundance and diversity decreased from G2 to G3. This suggests that ToMoLCV and ToSRV outcompeted other begomoviruses from G1 to G2 and that ToSRV was being outcompeted by ToMoLCV from G2 to G3. The possible evolutionary history of begomoviruses that were likely transferred from wild native plants and weeds to tomato crops after the introduction of the polyphagous vector *Bemisia tabaci* MEAM1 and the wide use of cultivars carrying the *Ty-1* resistance gene are discussed, as well as the strengths and limitations of the use of HTS in identification and diversity analysis of begomoviruses.

## Introduction

The cultivation of tomato (*Solanum lycopersicum*) is challenging due to its susceptibility to several pathogens, including begomoviruses (family *Geminiviridae*; genus *Begomovirus*), a major group of plant pathogens found in tropical and subtropical regions (Rojas et al., 2018). Begomoviruses present small, circular, single-stranded (ss) DNA genomes and are transmitted by whiteflies of the *Bemisia tabaci* cryptic species complex. The genome is composed of either two components (bipartite)—DNA-A and DNA-B—each comprising ~2.6 kb or a single component (monopartite, corresponding to the DNA-A component of bipartite begomoviruses) of ~2.8 kb (Fauquet et al., 2003; Brown et al., 2012). The rate of 91% genome-wide nucleotide identity of the complete genome (or DNA-A for bipartite begomoviruses) is used as threshold for species demarcation in the genus *Begomovirus* (Brown et al., 2015).

Begomoviruses exhibit high mutation and recombination rates, both within and among species, resulting in the rapid adaptive evolution and emergence of new variants and species (Duffy et al., 2008; Rocha et al., 2013; Silva et al., 2014; Lima et al., 2017). Historically, the first reported begomoviral disease in tomatoes in Brazil was caused by the tomato golden mosaic virus (TGMV), a New World (NW) bipartite begomovirus, in the 1960s (Costa, 1976). The disease is characterized by distorted growth and yellow to light green mosaic in leaves and is transmitted by whiteflies (Matyis et al., 1975). Thereafter, the disease was either not reported or was at an undetectable level in the country. It is speculated that this low occurrence of begomoviruses in tomato plants was due to the host preference of the *B. tabaci* populations present in Brazil at that time, presumably of the cryptic species *B*. *tabaci* New World (NW), previously known as biotype A. However, after the introduction of the species *B. tabaci* Middle East Asia Minor 1 (MEAM1) in the early 1990s the situation changed, as MEAM1 is more polyphagous and readily attracted to tomatoes; this resulted in a fast and widespread occurrence of begomoviral diseases in Brazil (Faria et al., 1997; Ribeiro et al., 2003; Fernandes et al., 2006; Castillo-Urquiza et al., 2008). Unlike whiteflies of the NW, MEAM1 has a broad range of hosts and is believed to have transferred native viruses from weeds and wild plants to cultivated tomato plants (Castillo-Urquiza et al., 2008; Barreto et al., 2013). Currently, 25 species of tomato-infecting begomoviruses have been described in Brazil (e.g., Flores et al., 1960; Matyis et al., 1975; Ribeiro et al., 2003; Fernandes et al., 2006; Castillo-Urquiza et al., 2008; Fernandes et al., 2008; Albuquerque et al., 2012; Rocha et al., 2013). Bipartite begomoviruses, especially tomato severe rugose virus (ToSRV; Inoue-Nagata et al., 2016; Rojas et al., 2018; Mituti et al., 2019), are the most predominant in tomato plants. The monopartite tomato mottle leaf curl virus (ToMoLCV) (Vu et al., 2015) has also been largely reported in tomato plants and causes severe symptoms such as chlorotic spots, interveinal chlorosis, mottling, mosaic, leaf distortion, and stunting (Inoue-Nagata et al., 2016).

Traditionally, the most common preventive measures for begomoviral disease control are the application of insecticides against the vector and the use of resistant cultivars (Lapidot and Friedmann, 2002; Hurtado et al., 2012). To date, genes of the series *Ty-1* to *Ty-6* (and a few others such as *tcm-1* and *ty-5*) were reported to provide resistance/tolerance to tomato yellow leaf curl virus (TYLCV), the most widespread begomovirus in the world (e.g., Zamir et al., 1994; Giordano et al., 2005; Anbinder et al., 2009; Ji et al., 2009; Hutton et al., 2012; Bai et al., 2018; Gill et al., 2019); some of these genes can provide a moderate level of control against NW begomoviruses (Giordano et al., 2005; Boiteux et al., 2007; Aguilera et al., 2011). For example, an experimental heterozygous hybrid carrying *Ty-1* was less infected and displayed milder or no symptom under infection of the Brazilian begomoviruses tomato rugose mosaic virus (ToRMV) and tomato yellow vein streak virus (ToYVSV), and thus classified as tolerant to these viruses (Boiteux et al., 2007). These resistance genes have been successfully introgressed into tomato lines in breeding programs. Of those, many commercial tomato hybrids carrying the *Ty-1*/*Ty-3* (allelic) genes (Verlaan et al., 2013) are largely in use in Brazil (Pereira-Carvalho et al., 2015). *Ty-1*/*Ty-3* encode a plant RNA-dependent RNA polymerase, which activates transcriptional gene silencing with an increase in viral genome methylation (Verlaan et al., 2013; Butterbach et al., 2014). In plants that carry resistance alleles, viral accumulation continues, although at lower levels than in susceptible plants (Zamir et al., 1994; Belabess et al., 2016).

Based on the overwhelming diversity of begomoviruses in tomato plants in Brazil and the prevalence of only two begomovirus species (Inoue-Nagata et al., 2016; Rojas et al., 2018; Mituti et al., 2019), we hypothesize that several native begomoviruses were introduced from wild and weed plants to tomato and that their diversity decreased over time after intensive interactions between viruses, vectors, hosts, and environmental factors. Furthermore, the broad use of resistant tomato cultivars carrying *Ty-*like may also have influenced the begomovirus population dynamics in the field, as reported earlier for tomato yellow leaf curl disease (García-Andrés et al., 2009). This hypothesis assumes that selective pressure is posed by resistance genes, limiting species diversity over the years in a local environment. In this study, we estimated genetic diversity changes in begomoviruses that infect tomato plants in an important tomato-growing region in Central Brazil through ~14 years. For temporal analysis, begomovirus-infected plant samples were divided into three groups, and all begomovirus sequences were identified using high-throughput sequencing (HTS) data. Subsequently, species-specific PCR and Sanger sequencing were done to confirm the sequences.

## Materials and Methods

### Collection of Tomato Samples and Detection of Begomovirus

Symptomatic tomato leaves (with chlorotic spots, interveinal chlorosis, and leaf curling; **Supplementary Figure 1**) were collected from 2003 to 2016 in a ~400 km^2^ area comprising Taquara and Pipiripau, located at the Federal District, at altitudes varying from 905 to 1225 m above sea level (**Supplementary Table 1**)—two of the major tomato production areas in Central Brazil. In this region, tomatoes are grown for the fresh market. Begomovirus infections were previously confirmed by total DNA extraction using the CTAB method (Doyle and Doyle, 1990) and PCR amplification using universal primers (PAR1c496 and PAL1v1978; Rojas et al., 1993). To analyze temporal population change in the genetic variants of begomovirus, we divided the samples into three groups according to the collection year: G1 (2003, 2004, 2005; N = 107), G2 (2009, 2010, 2011; N = 118), and G3 (2014, 2015, 2016; N = 129). Fields visited in G1 to G3 (**Supplementary Table 1**) equally represented the surveyed area.

### Diversity Analysis of Begomoviruses by Rolling Circle Amplification and RFLP

Circular viral DNA was amplified in individual samples by rolling circle amplification (RCA) using the illustra TempliPhi DNA amplification kit (GE Healthcare, Milwaukee, USA), following the manufacturer’s instructions and random hexamer primers, which efficiently amplifies circular DNA molecules at random. Each RCA product was digested with MspI and the digestion profile was analyzed by 1% agarose gel electrophoresis.

### HTS of RCA Products From Each Group and Identification of Begomoviruses

Three independent libraries were prepared, each containing a different pool of RCA products, namely G1, G2, and G3 (as above). The libraries were prepared using the TruSeq DNA sample preparation v.2 kit and sequenced using 100 bp paired-end reads on the Illumina Hiseq 2000 platform (Macrogen Inc., Seoul, South Korea). The HTS reads were trimmed using Trimmomatic (Bolger et al., 2014), and the contigs were *de novo* assembled using Velvet and MEGAHIT v1.1.3 (Phred = 34, 71 k-mer) (Zerbino and Birney, 2008; Li et al., 2015). The sequences assembled by these two assemblers were transferred to Geneious 8.1.9 (Biomatters, Auckland, New Zealand) and subjected to a BLAST search against the geminivirus reference database (downloaded from NCBI on 28/01/2019). The short contigs (<100 nucleotides) or those containing repeated sequences were re-blasted for confirmation. For every detected virus from contigs of both assemblers, the reference sequence was used to map the reads in Geneious (using the map to reference tool), and thus consensus sequences were assembled. After manual verification of the genome coverage, only a fully covered genome was considered for further analyses. Pairwise comparisons of the full genome were performed using Sequence Demarcation Tool program (SDT, Muhire et al., 2014). To confirm the presence of various begomoviruses detected in the pooled samples by HTS analyses, PCR with specific primers for complete/partial genomic regions (**Supplementary Table 2**) was performed using the three pooled samples. Then primers were designed to amplify the complete DNA-A and DNA-B segments (**Supplementary Table 2**) of the confirmed viruses in the pooled samples, and the amplicons were directly sequenced by the Sanger method at Macrogen, both by using PCR primers and primer walking. More than one primer pair were used for some viruses (**Supplementary Table 2**).

### Estimation of Intraspecies Diversity of ToMoLCV and ToSRV Sequences

Genetic diversity and population dynamics of begomoviruses through time were estimated using the HTS data. In this analysis, the reads were mapped to three reference databases comprising all ToMoLCV, ToSRV, and begomovirus sequences available in GenBank using BWA MEM v.0.7.17 (Li, 2013) with a seed length (-k) of 55 nucleotides. We opted for using the number and frequency of unique k-mers extracted from the aligned reads to estimate the diversity in order to mitigate cross alignments between different species, which should be common considering the 91% nucleotide identity thresholds for species demarcation (Brown et al., 2015). Twenty-seven mers were extracted and counted from the reads that aligned to each database using SAMtools v1.9 (Li et al., 2009) and Jellyfish v.2.2.3 (Marçais and Kingsford, 2011). Shannon entropy (Shannon, 1948) was calculated for each data set based on the frequency of each unique 27-mer. The number of reads aligned to each database was used to calculate the relative abundance of ToSRV and ToMoLCV for each sample group. To investigate whether the diversity of ToSRV and ToMoLCV significantly changed over time, we aligned previously extracted reads mapped to the genomes of these viruses in order to annotate single nucleotide polymorphisms (SNP) with LoFreq (Wilm et al., 2012). Thereafter, the entropy of each SNP was calculated and the cumulative sum of the entropy was used to perform the Wilcoxon signed-rank tests between two time points.

### *Ty-1* Detection in Individual Total DNA Samples

Considering that *Ty*-1 is the most common resistance gene introgressed in commercial cultivars in Brazil, PCR was performed to confirm its the presence in individual samples, as described by Prasanna et al. (2015). The amplicons were digested with HinfI and polymorphism was evaluated. The DNA profile from cultivars carrying *Ty-1* yields a single ~1 kb DNA fragment, in contrast to the ~0.6 kb fragment in those not carrying the gene.

### Detection of the Major Begomoviruses in Individual Samples

The four most frequent begomoviruses in the data sets were selected: ToSRV, ToMoLCV, tomato golden vein virus (TGVV), and Sida micrantha mosaic virus (SiMMV). Thereafter, PCR was performed to evaluate their presence in individual total DNA samples within groups G1, G2, and G3 (primers listed in **Supplementary Table 2**).

### Phylogenetic Analysis

Phylogenetic analyses were performed with the complete set of DNA-A and DNA-B sequences of all viruses identified in this study. All sequences related to these viruses were retrieved from GenBank and aligned using MUSCLE. The phylogenetic tree was constructed using the Tamura-Nei model (Tamura and Nei, 1993) and the maximum likelihood statistical method with 3000 repetitions included in the MEGAX program (Kumar et al., 2018). The reference sequence of the most closely related virus detected by BLAST search was used as outgroup.

## Results

### Diversity Analysis by RCA-RFLP

**Figure 1:**
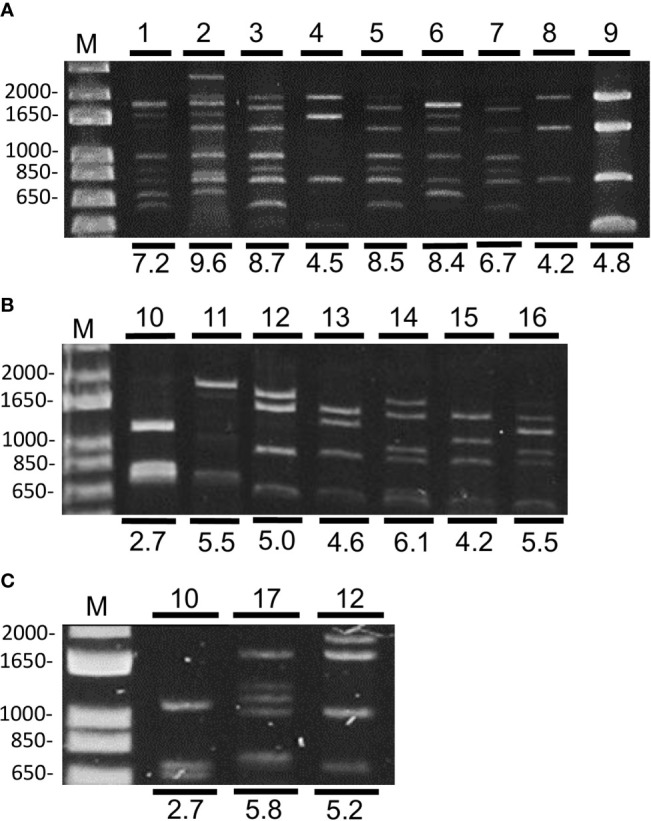
Restriction enzyme digestion profiles of begomoviruses amplified by RCA and digested with MspI on 1% agarose gel, observed in representative samples of G1 **(A)**, G2 **(B)**, and G3 **(C)**. The estimated sum of each genome fragment (in kbp) is indicated below the restriction profile. M = 1 kb plus DNA ladder (Thermo Fisher Scientific).

A collection of begomoviruses sampled in the Taquara and Pipiripau regions was used for begomovirus diversity analysis (**Supplementary Table 1**). A subset of these samples was divided into three groups according to the collection year: G1 (2003–2005), G2 (2009–2011), and G3 (2014–2016). The circular DNAs of individual samples from each group were amplified by RCA and digested with MspI for a preliminary evaluation of the diversity of begomoviruses in the samples, as viruses of the same species share a similar digestion profile. Summing the length of each fragment showed the genome size of the begomovirus to be ~2.7 kb for monopartite viruses and ~5.2 kb for bipartite viruses. The digestion profiles were analyzed individually and grouped into 17 distinct pattern profiles (representative profiles shown in **Figure 1**). The size of potential begomovirus genomes was estimated to range from 2.7–9.6 kb (numbers below the electrophoresis image, **Figure 1**), suggesting the presence of a monopartite begomovirus in some samples and mixed infections in others. In the G1 group, most of the profiles were mixed infection types, as the sum exceeded 6.7 kb (**Figure 1A**). In contrast, samples of the G2 and G3 groups likely contained either a monopartite or bipartite begomovirus (**Figures 1B, C**). Nine digestion profiles were observed in G1, seven were observed in G2 and only three in G3 (**Figure 1**). This suggested a local decrease in begomovirus diversity over time. Two identical profiles were observed in G2 and G3 (profiles 10 and 12, **Figures 1B, C**), indicating that the same viruses were potentially present in these groups, whereas those patterns observed in G1 were not found in the other groups. From these results, we concluded that a substantial change in the diversity of begomovirus species occurred in the evaluated time.

### A Gradual Decrease in Begomovirus Diversity From 2003 to 2016 Is Confirmed by Metagenomic Studies

Illumina sequencing generated a total of 25,522,962 reads from G1 (2003–2005), 21,442,638 reads from G2 (2009–2011), and 19,960,206 reads from G3 (2014–2016) (**Supplementary Table 3**). Removal of adapter residues and low-quality sequences yielded > 18,000,000 final reads each (**Supplementary Table 3**). Two assembling programs, Velvet and MEGAHIT, were evaluated for the number and size of the contigs. The number of contigs was higher for Velvet (**Supplementary Table 4**); however, they were in average shorter (141 to 1,089 bp) than those from the MEGAHIT assembler (406 to 2,631) (**Supplementary Table 5**). The BLAST results of contigs by both assemblers were highly similar, with contigs sharing high identities with seven viruses in G1, three in G2, and three in G3 (**Supplementary Tables 4** and **5**). Only one complete genome sequence containing 2,631 bases and sharing 98.29% identity with an isolate of ToMoLCV (**Supplementary Table 5**) was assembled. The remaining contigs were partial sequences of either DNA-A or DNA-B components.

After all reads were fine mapped using the reference sequence of the viruses corresponding to each contig, 19 genome-sized (~2.6 kb) sequences were assembled with full coverage (**Table 1**). In one case, when the reads were mapped to the ToMoLCV reference sequence (KX896398), the consensus sequence shared 89.47% identity with ToMoLCV accession KC706615, and 87.87% with the reference sequence, being distinct from the sequence assembled by MEGAHIT. This sequence was identified as Bego1:BR:G1, a potentially new begomovirus. In contrast, the consensus ToMoLCV sequence assembled by MEGAHIT (**Supplementary Table 5**) shared 91.97% with the ToMoLCV reference sequence. Hence, these two ToMoLCV-like sequences were included in **Table 1**.

**Table 1:** Consensus viral sequences identified by high-throughput sequencing in individual libraries G1, G2, and G3 and nucleotide comparison with reference sequence of the closest begomovirus.

Group		Identification/Genomic component	Consensus length^1^	Coverage	Reads	Closest begomovirus	Percentage identity (%)^2^	Reference accession^3^
1	1	SiMMV : BR:G1 DNA-A	2691	100	2 610 718	SiMMV DNA-A	89.12 [94.09]	AJ557451
	2	ToCMoV : BR:G1 DNA-A	2622	100	4 503 800	ToCMoV DNA-A	92.12	AF490004
	3	ToCMoV : BR:G1 DNA-B	2577	100	1 566 484	ToCMoV DNA-B	85.78 [92.65]	AF491306
	4	TGVV : BR:G1 DNA-A	2563	100	2 628 102	TGVV DNA-A	98.01	JF803254
	5	TGVV : BR:G1 DNA-B	2530	100	736 932	TGVV DNA-B	99.37	JF803265
	6	ToMoLCV : BR:G1	2631	100	2 137 458	ToMoLCV	91.97	KX896398
	7	ToRMV : BR:G1 DNA-A	2620	100	6 629 911	ToRMV DNA-A	96.07	AF291705
	8	ToSRV : BR:G1 DNA-A	2592	100	8 275 251	ToSRV DNA-A	99.38	DQ207749
	9	ToSRV : BR:G1 DNA-B	2572	100	4 349 407	ToSRV DNA-B	99.53	EF534708
	10	Bego1:BR:G1	2632	100	2 507 736	ToMoLCV	87.87 [89.47]	KX896398
	11	ToALCV : BR:G1	2875	100	32 211	ToALCV	95.23	MG491196
2	1	SiMMV : BR:G2 DNA-A	2693	100	2 507 684	SiMMV DNA-A	89.23 [92.75]	AJ557451
	2	ToMoLCV : BR:G2	2631	100	3 485 577	ToMoLCV	91.52	KX896398
	3	ToSRV : BR:G2 DNA-A	2593	100	7 891 080	ToSRV DNA-A	99.50	DQ207749
	4	ToSRV : BR:G2 DNA-B	2572	100	4 838 835	ToSRV DNA-B	99.38	EF534708
3	1	ToMoLCV : BR:G3	2633	100	6 739 860	ToMoLCV	92.12	KX896398
	2	ToSRV : BR:G3 DNA-A	2593	100	3 498 480	ToSRV DNA-A	98.92	DQ207749
	3	ToSRV : BR:G3 DNA-B	2570	100	4 739 156	ToSRV DNA-B	98.25	EF534708
	4	Bego2:BR:G3	2617	100	2 067 809	BGMV DNA-A	89.30 [90.44]	M88686

^1^Sequences are in **Supplementary Table 6**.

^2^Nucleoitide identity of the genome-wide consensus sequence with the reference genome, calculated by SDT (Muhire et al., 2014). In square brackets, comparison with the closest begomovirus sequence for those with identity <91%, SiMMV G1 DNA-A compared to JX415187, ToCMoV G1 DNA-B to KC706562, ToMoLCV G1 to KC706615, SiMMV G2 DNA-A to JX415194, BGMV G3 to KJ939806.

^3^Accession number of the reference sequences.

In the G1 group, seven begomoviruses were identified from 10 segments: (1) SiMMV (DNA-A), (2, 3) tomato chlorotic mottle virus (ToCMoV; DNA-A and DNA-B), (4, 5) TGVV (DNA-A and DNA-B), (6) ToMoLCV (monopartite), (7) ToRMV (DNA-A), (8, 9) ToSRV; (DNA-A and DNA-B), and (10) Bego1:BR:G1 (DNA-A) (**Table 1**). In addition, the genome of another geminivirus, the capula-like virus (11) tomato apical leaf curl virus [ToALCV, monopartite; Vaghi Medina et al., 2018)] was identified. Although SiMMV is a bipartite begomovirus, its DNA-B sequence could not be detected. The DNA-B of ToRMV was not found, but it is known to share a high nucleotide identity to ToSRV (Silva et al., 2014). Several reads were mapped to ToYVSV sequences (data not shown), which is highly related to TGVV. In fact, all ToYVSV-like reads were clearly mapped to TGVV sequences, and the ToYVSV genome could not be assembled using our data.

From the G2 group, three viral genomes were assembled (**Table 1**): (1) SiMMV (DNA-A), (2) ToMoLCV, and (3, 4) ToSRV (DNA-A and DNA-B). These three viruses were also detected in G1, suggesting that they are well-adapted to the tomato crop and remained in the evaluated region. The SiMMV sequences from G1 and G2 shared 95.13% nucleotide identity, indicating that they belonged to the same strain (i.e., > 94% nucleotide identity). They shared <90% genome-wide nucleotide identity with the reference sequence of SiMMV (AJ557451, **Table 1**). However, the closest match of SiMMV DNA-A of G1 was SiMMV accession JX415187 (from *Sida* sp.) sharing 94.09% identity, and of G2 was SiMMV accession JX415194 (from *Sida santaremnensis*) with 92.75% identity; thus, they were identified as SiMMV isolates: SiMMV : BR:G1 and SiMMV : BR:G2, respectively. Similar to the results for G1, the DNA-B of SiMMV was undetected. This suggests that SiMMV DNA-A might use the ToSRV DNA-B if SiMMV needs DNA-B for infection, as the three viruses—SiMMV, ToSRV, and the monopartite ToMoLCV—were detected in G2 samples. Another possibility is that the DNA B from this particular virus was under a detectable level or was outcompeted by other small circular DNA templates.

In G3, three viruses were identified: (1) ToMoLCV, (2, 3) ToSRV (DNA-A and DNA-B), and Bego2:BR:G3 (DNA-A) (**Table 1**). The genome of Bego2:BR:G3 shares 89.30% nucleotide identity with bean golden mosaic virus (BGMV) accession M88686 (reference sequence), and 90.44% with the closest begomovirus, the BGMV accession KJ939806; and it was provisionally named as Bego2. A BGMV DNA-B-like sequence was not found in the HTS reads. This result implies that ToMoLCV and ToSRV persisted in the area for over 14 years, whereas the other six viruses detected in G1 were not detected in the last samplings (G2 and G3). By comparing the sequences of ToSRV, ToMoLCV, and SiMMV from the three groups, nucleotide identities ranging from 95.13% to 99.80% were obtained, indicating their genomes accumulated non-lethal mutations. Furthermore, a thorough search for begomovirus-related satellites yielded no results for the three HTS data sets.

### Intraspecies Diversity Analysis

**Figure 2:**
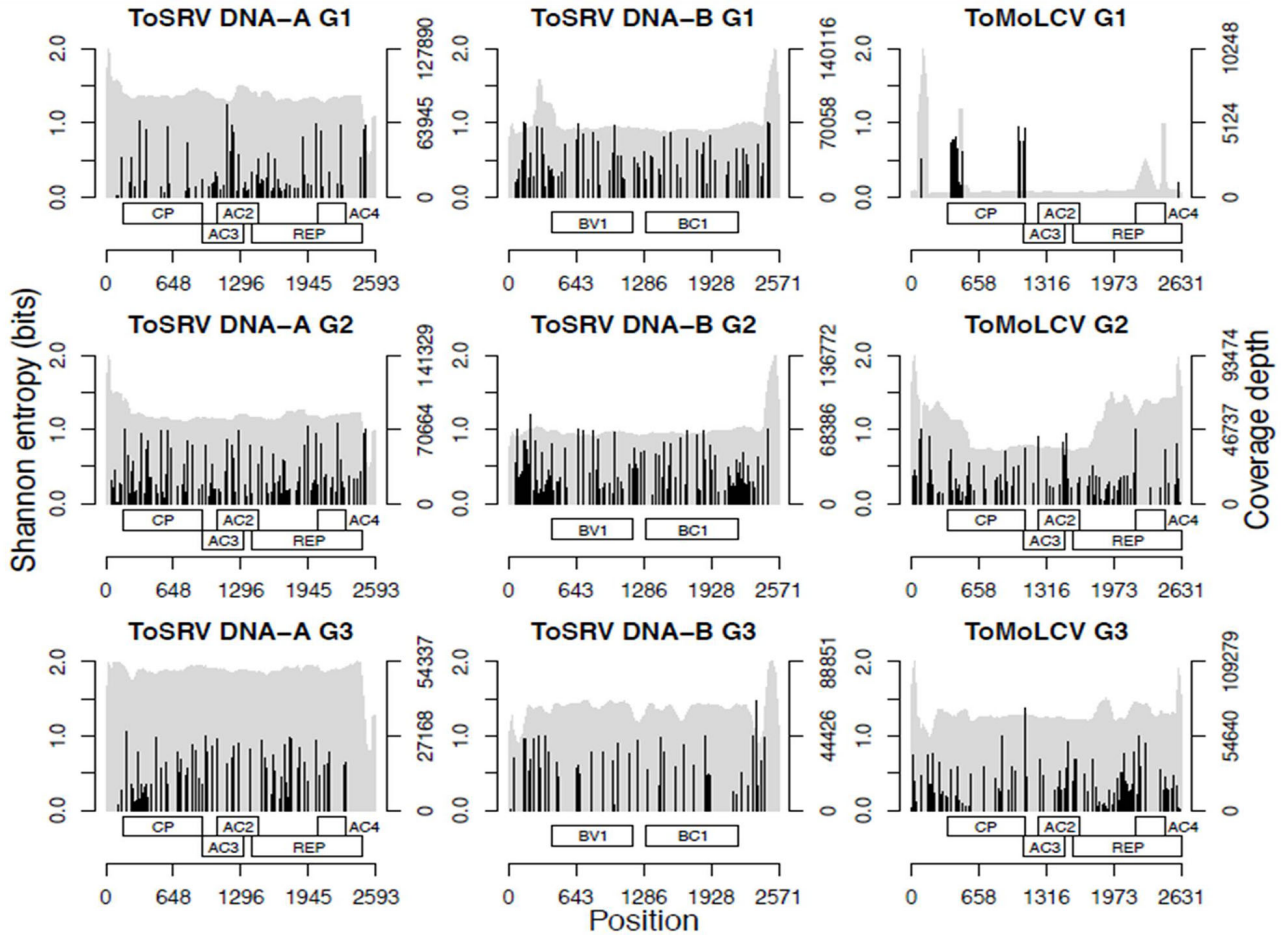
Nucleotide diversity analysis of ToSRV and ToMoLCV showing coverage depth (gray) and Shannon entropy for the SNPs (black) and a representation of genome organization.

Two parameters, unique k-mer count and Shannon entropy of unique k-mers, were used to estimate intraspecies diversity of ToMoLCV and ToSRV, the viruses found in all three groups, and of all begomoviruses for the G1, G2, and G3 data sets (**Table 2**). Additionally, total Shannon entropy, based on the frequency of each SNP of ToMoLCV and ToSRV, was calculated and the cumulative sum was used to test for a significant increase or decrease in diversity based on the Wilcoxon signed-rank test (**Figure 2**). The number of unique 27-mers of ToSRV and ToMoLCV compared to that of all begomoviruses suggests that these two species share several 27-mers. The overall diversity of begomoviruses decreased from 2003 to 2016 (G1–G3); conversely, the diversity of ToMoLCV increased from G1 to G3 and that of ToSRV increased from G1 to G2 but decreased from G2 to G3 (**Table 2**). Overall, the genetic diversity of ToSRV and ToMoLCV varied according to their relative abundance, however, the diversity of ToSRV DNA-B alone decreased from G2 to G3 while its relative abundance increased (**Table 2**). Based on total Shannon entropy, the diversity of SNPs followed a similar trend (**Table 2**), although in this case the diversity of DNA-B of ToSRV at G3 was smaller than that at G1. Importantly, in the latter diversity analysis, indels and epistasis are not accounted for. All Wilcoxon signed-rank tests of the cumulative SNP entropy between two time points were significant (*P* < 1e-16). Although SNPs appear to be concentrated in two regions of the ToMoLCV genome at G1, this result should be looked at with caution. These polymorphisms are located at the beginning or at the end of coding regions and likely represent cross alignment of reads from closely related species.

**Table 2:** Genetic diversity and relative abundance of ToSRV, ToMoLCV and all begomoviruses detected from 2003 to 2016.

Group		Virus	Unique 27-mers	27-mer Shannon entropy	Total SNP Shannon entropy	Total number of reads	Relative abundance
1	1	ToMoLCV	248 458	11.24	13.39	428 057	0.02
	2	ToSRV	3 493 062	14.07	72.31	11 606 934	0.67
	3	ToSRV DNA-A	2 099 063	13.12	29.77	7 718 716	0.45
	4	ToSRV DNA-B	1 448 480	13.24	42.53	3 888 218	0.22
	5	Begomovirus	6 555 088	15.20	NA	17 136 500	1
2	1	ToMoLCV	1 240 059	13.13	38.77	2 584 634	0.18
	2	ToSRV	4 369 323	14.59	112.57	11 714 792	0.81
	3	ToSRV DNA-A	2 446 504	13.58	51.52	7 531 010	0.52
	4	ToSRV DNA-B	2 002 928	13.91	61.04	4 183 782	0.29
	5	Begomovirus	5 611 647	15.02	NA	14 349 739	1
3	1	ToMoLCV	1 762 003	13.38	42.84	5 833 049	0.45
	2	ToSRV	2 340 172	14.25	73.73	6 867 494	0.53
	3	ToSRV DNA-A	1 159 760	13.37	39.67	2 943 937	0.23
	4	ToSRV DNA-B	1 222 633	13.29	34.05	3 923 557	0.30
	5	Begomovirus	4 079 349	14.84	NA	12 771 519	1

### Validation of Begomovirus Sequences Identified by HTS Using PCR and Sequencing

**Figure 3:**
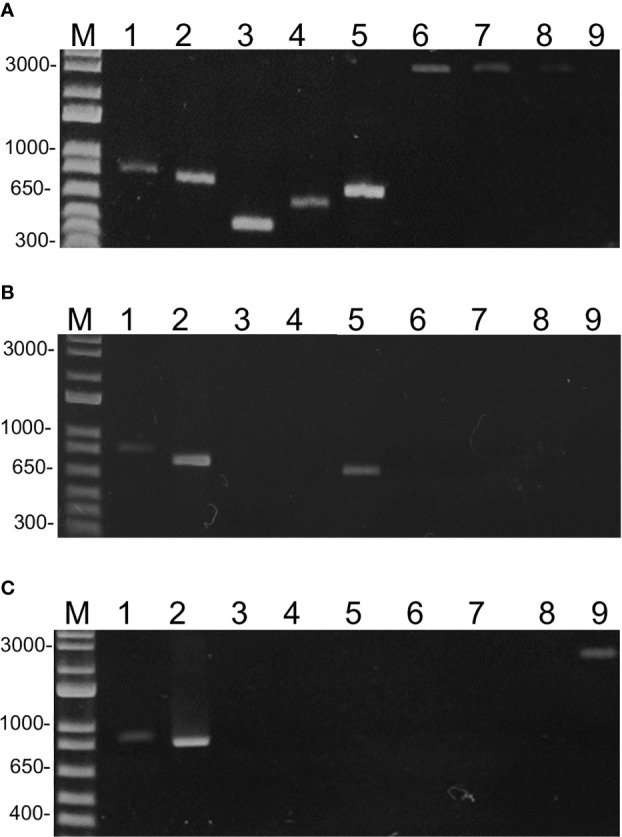
Electrophoretic analysis of PCR amplicons on 1% agarose gel from pooled RCA samples of the G1 **(A)**, G2 **(B)**, and G3 **(C)** groups. PCR was performed using specific primers (**Supplementary Table 1**) for ToSRV (1), ToMoLCV (2), TGVV (3), ToCMoV (4), SiMMV (5), ToALCV (6), ToRMV (7), Bego1 (8), and Bego2 (9). M = 1 kb plus DNA ladder (Thermo Fisher Scientific).

To demonstrate that the geminivirus sequences were not artifacts of the assembly programs, we performed PCR using species-specific primers (**Supplementary Table 2**). Based on the assembled, complete sequences, primers were designed to amplify the entire or partial DNA-A of TGVV, SiMMV, ToMoLCV, ToSRV, ToCMoV, Bego1, Bego2, ToRMV, and ToALCV, producing amplicons of ~0.5 to ~2.9 kbp (**Supplementary Table 2**). SiMMV detection was confirmed in G1 and G2, TGVV, ToCMoV, ToALCV, ToRMV, and Bego1 in G1, Bego2 in G3, and ToMoLCV and ToSRV in pooled samples of G1, G2, and G3 (**Figure 3**). Furthermore, PCR-specific to the DNA-B sequences (**Supplementary Table 2**) confirmed the presence of DNA-B of ToSRV, ToCMoV, and TGVV in the corresponding group, but not of SiMMV DNA-B in the three groups (data not shown). These results confirmed the begomovirus identification by analyzing the HTS data sets of G1, G2, and G3.

Next, PCR was performed to amplify the complete genome (DNA-A and DNA-B, **Supplementary Table 2**) of each virus in the pools and hence to compare with the HTS-assembled sequences. The amplicons were directly sequenced and used to assemble the final consensus sequence (**Table 3**). The amplicon of ToRMV resulted in a ToSRV sequence, and the one of Bego1 in a ToMoLCV sequence. Alignments of the HTS-assembled ToRMV and Bego1 sequence with other begomovirus sequences showed that the ToRMV sequence was in fact a chimera between ToSRV and ToCMoV sequences, and Bego1 a chimera between ToMoLCV and ToSRV sequence, i.e., artifact sequences. Therefore, these two viruses, ToRMV and Bego1, were eliminated from the analysis. The Bego2 amplicon resulted in a sequence sharing >91% nucleotide identity with the DNA-A reference sequence of BGMV, and thus it was renamed BGMV : BR:G3 (**Table 3**).

**Table 3:** Consensus viral sequences identified by sequencing PCR amplicons in libraries G1, G2, and G3 and comparison with HTS and reference sequences.

Group		Identification/Genomic component	Genome length (Accession)^1^	Comparison with HTS^2^	Comparison with reference^3^
1	1	SiMMV : BR:G1 DNA-A	2676 (MT733803)	90.84*	94.39
	2	ToCMoV : BR:G1 DNA-A	2622 (MT733804)	98.09	92.75*
	3	ToCMoV : BR:G1 DNA-B	2536 (MT733805)	97.50	85.03*
	4	TGVV : BR:G1 DNA-A	2561 (MT733806)	98.71	98.20
	5	TGVV : BR:G1 DNA-B	2534 (MT733807)	99.37	98.93
	6	ToMoLCV : BR:G1	2631 (MT733810)	99.89	91.98*
	7	ToSRV : BR:G1 DNA-A	2592 (MT733808)	99.77	99.15
	8	ToSRV : BR:G1 DNA-B	2570 (MT733809)	99.73	99.26
	9	ToALCV : BR:G1	2875 (MT135209)	100.00	95.23
2	1	SiMMV : BR:G2 DNA-A	2691 (MT733814)	95.10	92.00*
	2	ToMoLCV : BR:G2	2631 (MT733813)	98.59	92.36*
	3	ToSRV : BR:G2 DNA-A	2592 (MT733811)	99.88	99.50
	4	ToSRV : BR:G2 DNA-B	2570 (MT733812)	99.88	99.34
3	1	ToMoLCV : BR:G3	2631 (MT733817)	99.92	92.12*
	2	ToSRV : BR:G3 DNA-A	2592 (MT733815)	99.31	99.27
	3	ToSRV : BR:G3 DNA-B	2570 (MT733816)	97.82	97.04
	4	BGMV : BR:G3 DNA-A	2619 (MT733818)	89.56*	96.48

^1^Accession number at GenBank.

^2^Nucleotide identity of the genome-wide PCR amplicon sequence with the corresponding genome assembled by HTS, calculated by SDT (Muhire et al., 2014).

^3^Nucleotide identity of the genome-wide sPCR amplicon sequence with the corresponding reference genome (see **Table 1**), calculated by SDT (Muhire et al., 2014).

*Percent identity below 94%.

The PCR-derived sequences shared >97% nucleotide identities with the corresponding HTS-derived sequences, except for SiMMV : BR:G1 DNA-A, SiMMV : BR:G2 DNA-A, and BGMV : BR:G3 (**Table 3**). The PCR amplicon sequence of SiMMV from G1 diverged almost 10% from the HTS consensus, while from G2 about 5%. The PCR amplicon sequence of BGMV shared 89.56% with the HTS-derived sequence, but 96.48% with the BGMV reference sequence (**Table 3**). The sequences of ToALCV from PCR and from HTS were completely identical, suggesting a highly homogenous population with genomes with minor point mutations. From 19 genomes, two (ToRMV and Bego1) were not confirmed, and two (SiMMV G1 and BGMV G3) were >9% divergent from the HTS-assembled sequence.

The number of reads used to assemble the begomovirus genomes out of the total reads in G2 and G3 was 93.12% and 90.33%, respectively. This indicates that <10% of reads were not mapped to begomovirus sequences. However, in G1 the reads exceed in 12.33% (26,840,363 out of 23,894,300; **Table 1**, **Supplementary Table 3**) from the total obtained reads. It clearly shows that some reads were used to assemble more than one genome, i.e., the genomes share short regions of high identity among the viruses. It makes the assembling process complex in the case of mixed population of highly related viruses. We hypothesized that this has caused incongruent assembly of SiMMV in G1 and BGMV in G3, and also of ToRMV and Bego1 in G1.

In a phylogenetic analysis of the complete genomes, the HTS-assembled sequences and the PCR-assembled sequences tightly clustered for most genomes (**Supplementary Figure 2**). The two exceptions were the DNA-A of SiMMV and BGMV. The HTS-assembled SiMMV G1 and G2 sequences were grouped, but were more distantly separated to the majority of SiMMV sequences. In contrast, the PCR-based sequences were closer to the other SiMMV sequences. In the BGMV tree, the PCR-derived sequence tightly clustered with other BGMV sequences, while the Bego2 sequence was distant from all of them, suggesting that these particular sequences generated by HTS assembly were not reliable.

### Detection of Selected Begomoviruses in Individual Total DNA Samples

**Figure 4:**
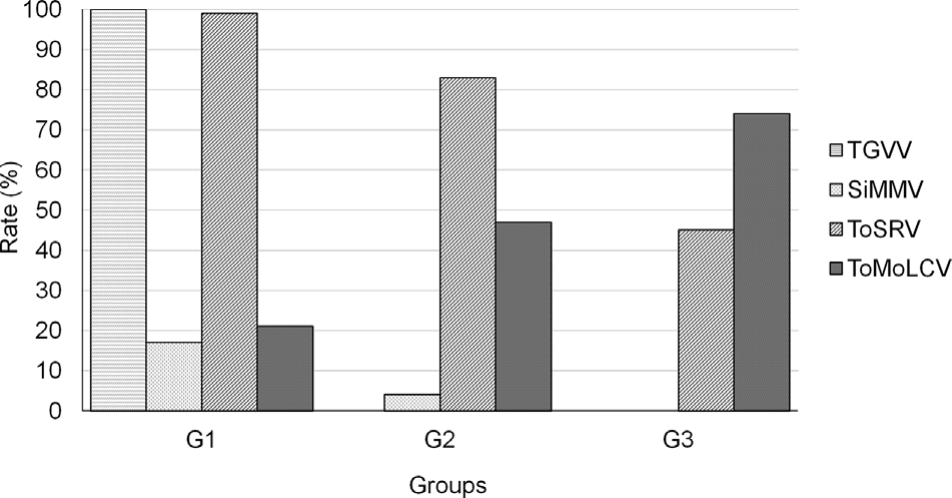
Detection rate of TGVV, SiMMV, ToSRV, and ToMoLCV by PCR in individual total DNA from the G1 (2003–2005), G2 (2009–2011), and G3 (2014–2016) groups.

It became clear that begomovirus diversity varied through time as did the prevalence of the viruses in each group. The four most frequent begomoviruses were: (1) SiMMV, (2) TGVV, (3) ToMoLCV, and (4) ToSRV. Consequently, to understand the frequency of each virus over time in the groups, we performed species-specific PCR to detect each of the four viruses in individual samples (**Figure 4**). In the G1 samples, a high detection rate of TGVV (100%) and ToSRV (99%) was observed, clearly indicating mixed infections. Approximately 21% of the plants were infected with ToMoLCV and 17% with SiMMV. In plants from G2 and G3, TGVV was not detected in any sample. SiMMV was detected in 4% of the samples in G2 and absent in G3. The rate of plants infected with ToSRV decreased from 99% in 2003–2005 to 83% in 2009–2011, and to 45% in 2014–2016. In contrast, ToMoLCV detection rate increased over time, from 21% in 2003–2005 to 47% in 2009–2011, and to 74% in 2014–2016, indicating that ToSRV and ToMoLCV were the most predominant viruses in this region.

### Detection of the Resistance Gene *Ty-1* in the Samples

**Figure 5:**
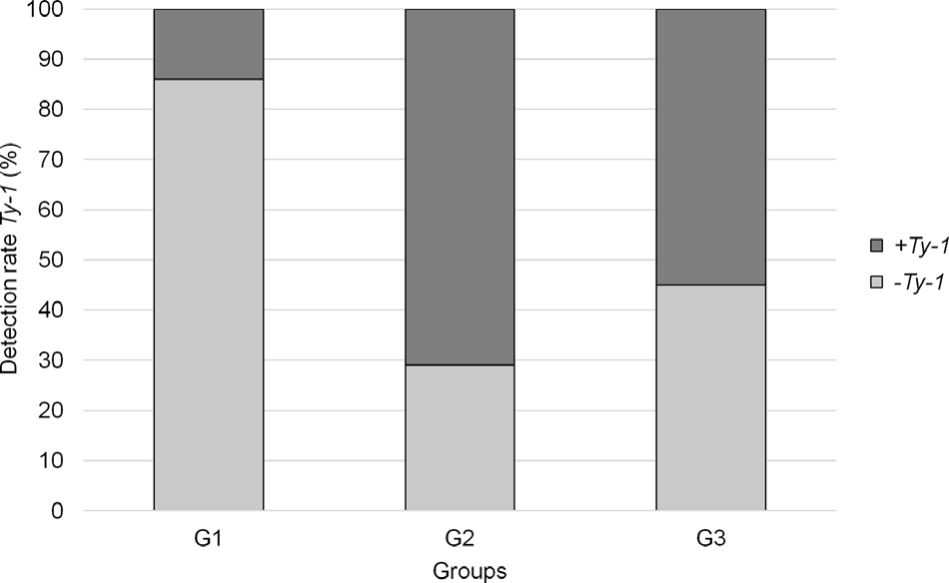
Detection rate of *Ty-1* in individual total DNA from the G1 (2003–2005), G2 (2009–2011), and G3 (2014–2016) sample groups.

The variation in begomovirus species composition and the shift in the predominant virus from samples collected between 2003 and 2016 were striking. Several factors may have contributed to this variation, but an increase in the use of resistant cultivars may have been one of the most important factors. Assuming *Ty-1* to be the major resistance gene in Brazilian commercial hybrids, PCR was performed to detect this gene in individual samples of the three groups (**Figure 5**). *Ty-1* was detected in 14% of G1 samples, suggesting that 86% plants were susceptible to begomovirus infection. In G2 and G3, the rate of *Ty-1* positive samples increased dramatically to 71% and 55%, respectively, indicating that it is an important trait for the growers.

## Discussion

HTS provides an easy and fast means for sequencing of viral genomes present in infected plant samples at a large scale (e.g., Idris et al., 2014; Rodríguez-Negrete et al., 2019). Although viral DNA could be directly sequenced, we enriched the circular DNA using RCA prior to library preparation for specifically targeting begomoviruses (Idris et al., 2014). Two *de novo* assembling programs, Velvet (Zerbino and Birney, 2008) and MEGAHIT (Li et al., 2015) were used. Although Velvet produced a high number of contigs, they were shorter than those from MEGAHIT, as reported by Blawid et al. (2017) and Jo et al. (2017) (**Supplementary Tables 4** **and** **5**). Velvet reduces the chances of missing low frequent reads, which may not assemble to a detectable contig by other assemblers that generate longer contigs, being this an advantage of this method (Blawid et al., 2017). MEGAHIT, on the other hand, produces longer contigs, which facilitates the assembling step and has a high sensitivity (Blawid et al., 2017). The final list of viruses was the same for both assemblers (**Table 1**, **Supplementary Tables 4** and **5**), indicating that the results were robust. However, the strategy of using 100 bp paired-end reads generated on the Illumina Hiseq 2000 platform for pooled highly related begomovirus samples proved to be challenging and could not be used as a stand-alone technique, as the final consensus sequence was significantly divergent in five (ToRMV, Bego1, Bego2, SiMMV G1 and SiMMV G2) out of 19 genomes. In the case of begomoviruses, it is therefore sensible that users avoid pooling samples, instead making use of barcoding to individualize samples, or by taking advantage of systems that generate longer sequences. Nevertheless, the analyses provided important information, such as the detection and identification of several begomoviruses and of one capula-like virus in the samples, which would be unlikely detected using traditional techniques as PCR/sequencing. Also important, it enabled the analysis of their diversity within the sampling pool, and through the three time points along the selected tomato cultivation area.

Analysis of ToSRV and ToMoLCV reads in the three libraries (**Figure 2**) showed that the coverage depth along the genome was reasonably uniform. A higher number of reads was concentrated at intergenic region of ToSRV, which may be related to overlapping reads in the common region shared by DNA-A and DNA-B. Exceptions were observed for ToMoLCV reads in G1 and G2, possibly correlating with highly conserved genomic regions among begomoviral species, and thus sharing many identical or near identical reads.

The uniform coverage of reads in the genome of ToSRV and ToMoLCV (**Figure 2**) also suggests that the amplification steps introduced by RCA and HTS were not biased and the entire genome was evenly covered. We do not discard, though, the possibility that an amplification bias was produced during the RCA step that could have introduced some artifacts.

We observed a remarkable population change in begomoviruses that infect tomatoes in the Federal District, Brazil, from 2003 to 2016. This study focused on samplings carried out within ~14 years, collected in an area of approximately 400 km^2^. This region is an important tomato-growing area of the Brasilia greenbelt, where tomato is intensively planted throughout the year. In the G1 sample set (2003–2005), containing isolates collected approximately ten years after the introduction of *B. tabaci* MEAM1 and after the first report of begomovirus in tomatoes in this region (Ribeiro et al., 1994), we detected six geminivirus species (including one capula-like virus). A decrease in species richness was observed between 2007–2016, with only three species detected in each group (**Table 3**). The high rate of mixed infection in G1 suggests that the prevalence of begomovirus was high in the area and the plants were susceptible to begomovirus infection. Thereafter, a decrease in the number of begomovirus species and intraspecies diversity was observed (**Table 3**). Although it confirmed our initial hypothesis of continuous reduction of viral diversity following their transfer from wild and weed plants to a new cultivated plant, this phenomenon was not observed for tomato yellow leaf curl virus (TYLCV) in China (5-year survey; Yang et al., 2014) or tomato yellow leaf curl Sardinia virus (TYLCSV) in Spain (8-year survey; Sánchez-Campos et al., 2002). However, Font et al. (2007) reported that the number of haplotypes of TYLCV and TYLCSV was reduced and resulted in the prevalence of one haplotype with low genetic diversity in a time span of four years. Furthermore, in a study on begomovirus diversity, focused on pepper golden mosaic virus (PepGMV) and pepper huasteco yellow vein virus (PHYVV), on chiltepin in Mexico, Rodelo-Urrego et al. (2013) reported a reduction in coat protein sequence diversity within a 4-year sampling period (2007–2010) and attributed it to the level of landscape heterogeneity and not the effect of virus-host co-evolution. We speculate that after the invasion of MEAM1, which colonized myriad plants (crops and wild and weed hosts) in the early 1990s, the vectors transferred indigenous begomovirus populations from these plants to tomatoes (Castillo-Urquiza et al., 2008; García-Arenal and Zerbini, 2019), thereby resulting in an explosion of begomoviruses in the highly permissive tomato plants. Later, intense anthropic actions, such as cultivation on a commercial-scale, host uniformity, and lower crop diversity, contributed to a high degree of viral competition, and thus the selection of the most fit, and on the decrease in the diversity of these begomoviruses, as also proposed by Pagán et al. (2012) and Rodelo-Urrego et al. (2013).

In this survey, six viruses, including the newly reported capula-like virus ToALCV with ~32,000 reads, were identified by HTS. ToALCV is a monopartite virus, and was recently described in Argentina (Vaghi Medina et al., 2018) and in the Federal District of Brazil (Batista et al., 2019). Thus, despite being recently described, the virus had been present in samples collected between 2003 and 2005. The vector of this virus is still unknown. DNA satellites associated to begomoviruses were not detected in our libraries, although alphasatellites have been previously reported in Brazil (Paprotka et al., 2010; Mar et al., 2017).

In this study, the use of >100 samples in at least eight farms for each grouping is believed to be sufficient to represent the begomovirus diversity in the region. One of the drawbacks of the sampling strategy was the selection of symptomatic plants. Although detecting begomoviral disease symptoms in resistant cultivars is simple, we may have generated a bias in searching for plants with stronger symptoms. However, in each field survey, an effort was made to cover all types of symptoms present in the area.

Notably, the DNA-A sequence of SiMMV was detected only in the G1 and G2 libraries, although at a low rate (**Figure 4**). SiMMV is usually associated with *Sida* spp., a widespread group of malvaceous weeds frequently found in association with tomato fields, and is an example of a virus that might be transferred from weeds to cultivated plants (Barreto et al., 2013). However, SiMMV DNA-B could not be detected by either HTS or PCR. As SiMMV DNA-A was found in G2 with ToSRV (always in mixed infection), it is possible that they share the DNA-B. This is because these two viruses are closely related and their DNA-A sequences share ~87% nucleotide identity. The analysis of the common region of all ToSRV and SiMMV sequences assembled by HTS and PCR revealed an identical core-iterated sequence (GGTAG-GGTAG), except for the genome of SiMMV G1 assembled by PCR (GGGGT-GGGGA). Thus, it is possible that SiMMV DNA-A uses the ToSRV DNA-B for infection. Infectious clones prepared from a bipartite isolate of SiMMV, ToSRV, and G1 and G2 SiMMV DNA-A, tested in all possible combinations by inoculation in plants would clarify this relationship, and this study is underway. Furthermore, a BGMV isolate is expected to be a bipartite virus, but attempts to detect the DNA-B of a BGMV-like virus failed (data not shown). It means that it may also share the DNA-B of ToSRV or it behaves as a monopartite virus. The core-iterated sequence was (GGTGT-GGTGC) which is different from those of ToSRV. Another hypothesis is that when SiMMV or BGMV DNA-A coinfect a plant with a monopartite begomovirus, the viral derived proteins of the monopartite virus complement the DNA-A of SiMMV or BGMV. However, these hypotheses, and also the possibility that the DNA-B was under a detectable level, need to be further tested.

Due to the epidemic of begomovirus disease in the major tomato-growing regions in Brazil soon after the outbreak of MEAM1, (moderately) resistant cultivars became available and were largely used in the G2 group (**Figure 5**). Other resistance genes may be present in Brazilian cultivars but only the most prevalent gene, *Ty-1* (Pereira-Carvalho et al., 2015), was tested. We demonstrated that *Ty-1* was present in 55%–71% plants collected at later time points (**Figure 5**). We speculate that this might have produced a bottleneck effect during 2006–2008 and resulted in SiMMV, ToMoLCV and ToSRV to be filtered and survive the selection pressure. Even though the use of resistant cultivars is an effective strategy for disease control, it depends on the preference of growers. In many cases, susceptible cultivars have desired traits such as higher yield, resistance to other pathogens, and bigger fruit size and fruit quality (Boiteux et al., 2007; Rubio et al., 2010).

We estimated the rate of plants infected with the four prevalent viruses—ToSRV, ToMoLCV, TGVV, and SiMMV—by DNA-A-specific PCR. Only ToSRV and ToMoLCV were detected in all three groups (G1, G2, and G3). Although the number of plants infected with ToSRV decreased over time, the rate of ToMoLCV infection increased, outcompeting ToSRV (**Figure 4**). Whether ToMoLCV performs better during infection than ToSRV in resistant cultivars or if it is transmitted more efficiently by the MEAM1 population present in the area remains to be elucidated. The rate of mixed infection decreased over time, from 100% to almost 0%, after 13 years. The rate of mixed infection of two begomoviruses, PepGMV and PHYVV, also decreased from one year to another in cultivated chiltepin fields, which can be attributed to a decrease in host heterogeneity and an increase in host density (Pagán et al., 2012; Rodelo-Urrego et al., 2013), similar to what happened in the sampled area. It suggests that after their establishment in a specific crop, under a certain vector population in an intensively cultivated area, the co-existence of two begomoviruses in the same plant is not a common phenomenon. In fact, some regions may have reached this stage with the predominance of ToSRV in the major tomato-growing areas of the country (States of Goiás, São Paulo, and Minas Gerais, Mituti et al., 2019), ToMoLCV in the North-East region (Fernandes et al., 2008; Inoue-Nagata et al., 2016; Rojas et al., 2018), and tomato common mosaic virus (ToCmMV) in the states of Espírito Santo and Rio de Janeiro (Barbosa et al., 2016).

Interestingly, in the G1 library, ToSRV reads corresponded to a relative abundance of 0.67, implying that this virus was more prevalent than the others (**Table 2**). The increase in relative abundance to 0.81 in the G2 library confirms that it is most fit in this agricultural environment, such as continuous tomato cultivation, mild tropical climate, standard cultivation system, and the presence of *B. tabaci* MEAM1. However, this rate decreased in the G3 library, whereas that for ToMoLCV showed a substantial increase, similar to the tendency seen in G1–G2 (**Table 2**). Taken together, this result and the detection of these viruses in most individual samples (**Figure 4**) demonstrate that ToSRV and ToMoLCV were the most successful tomato plant viruses in the area. This result must be carefully analyzed since an amplification step by RCA was added prior to HTS, which may influence population profiles in the samples. Notably, the number of tomato samples was similar in all three groups in an attempt to reduce the bias introduced by the amplification step. In the field of virus genome analysis, Shannon entropy (**Table 2**) measures diversity, based on haplotype frequencies (Gregori et al., 2016), and demonstrates the abundance of unique sequences. In our case, we used 27-mers frequencies to circumvent the difficulties in assembling haplotypes from 100 bp reads. An increase in Shannon entropy index was observed in ToMoLCV (G1 to G3), but the decrease in ToSRV (G2 to G3) confirmed that ToMoLCV became more diverse (and more frequent) than ToSRV isolates. As expected under neutral selection, the diversity and population size of ToMoLCV and ToSRV varied accordingly overall (Kimura, 1983). Exceptionally, genetic diversity of ToSRV DNA-B decreased from G2 to G3 although its relative abundance increased, suggesting purifying selection.

ToSRV and ToMoLCV are considered successful begomoviruses in this production system. Future studies may reveal that only one—ToMoLCV—predominates, based on the tendency. This scenario may change dramatically as new resistance genes are being used for introgression into commercial tomato hybrids. During the surveyed period, only *B. tabaci* MEAM1 was detected in the area (data not shown). The first report of the invasive species *B. tabaci* Mediterranean (Med) in Brazil occurred in 2015 (Barbosa et al., 2015). To date, it has not been reported in the central region, although it is rapidly spreading in the country (Moraes et al., 2018). We speculate that the introduction of Med whiteflies can change the incidence, prevalence, and diversity of begomoviruses in our crops in the coming years.

## Data Availability Statement

The datasets generated for this study can be found in the GenBank. MT135209, MT733803, MT733804, MT733805, MT733806, MT733807, MT733808, MT733809, MT733810, MT733811, MT733812, MT733813, MT733814, MT733815, MT733816, MT733817, MT733818.

## Author Contributions

TS and AI-N designed the experiments, and collected the samples. TS prepared the samples, analyzed the genetic polymorphism, performed the sequencing and validated the sequences. TS and TM amplified and sequenced the complete virus genomes. TS, JS, EN, TN and AI-N performed the bioinformatics analyses. TS, JS, TN, TM, EN and AI-N read, revised, and approved the manuscript.

## Funding

The research leading to these results has received funding from the Fundação de Apoio à Pesquisa do Distrito Federal (Project 0193.001460/2016) and from the Fundação de Amparo à Pesquisa de São Paulo (Project 2018/18274-3).

## Conflict of Interest

The authors declare that the research was conducted in the absence of any commercial or financial relationships that could be construed as a potential conflict of interest.
