# Supplementary figures and images for: A Temporal Diversity Analysis of Brazilian Begomoviruses in Tomato Reveals a Decrease in Species Richness between 2003 and 2016
Source: Front Plant Sci. 2020 Aug 6;11:1201. doi: 10.3389/fpls.2020.01201 (PMC7424291; doi:10.3389/fpls.2020.01201)

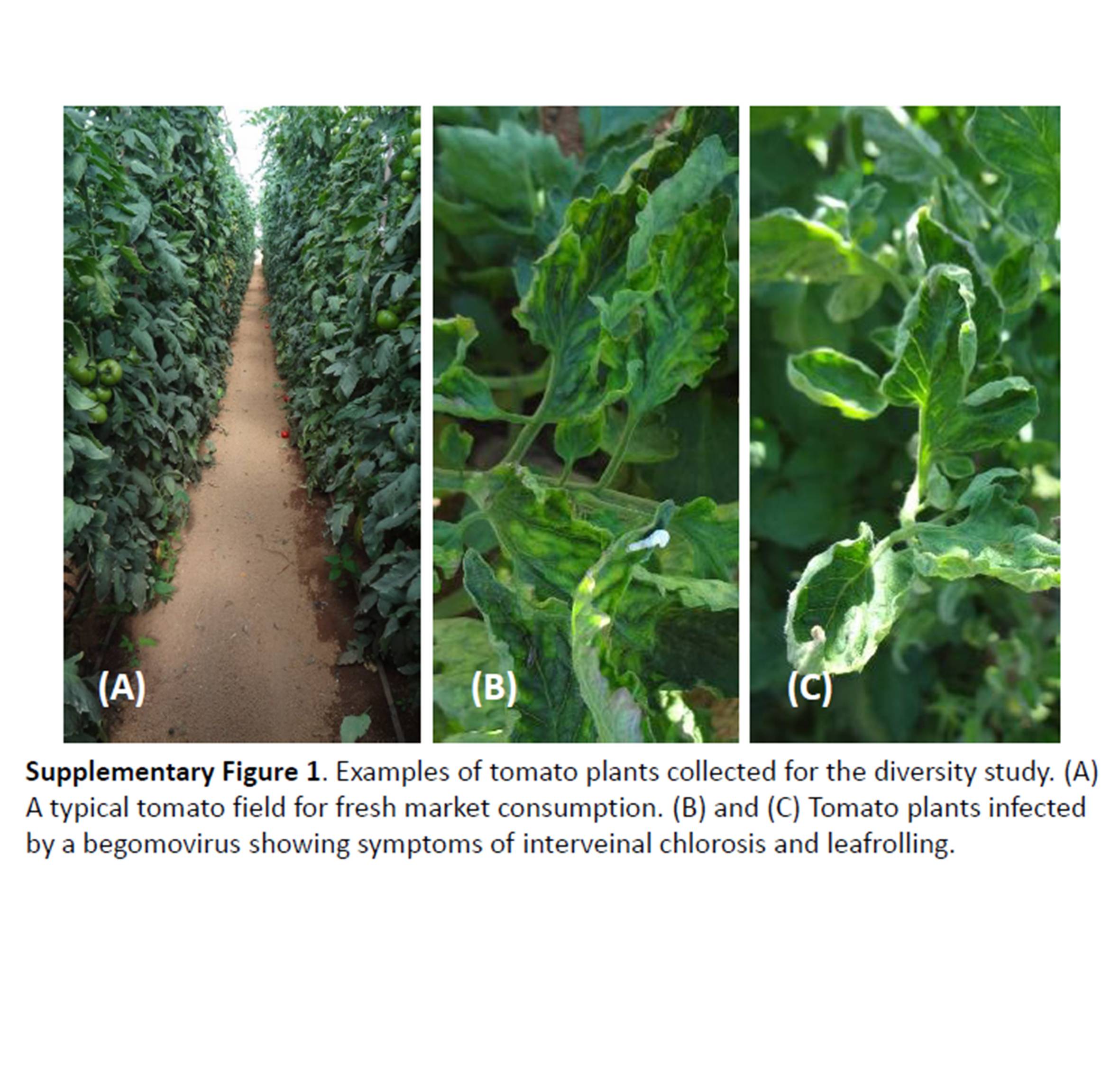

Supplement: Supplementary file 1 [file Image_1.jpeg]

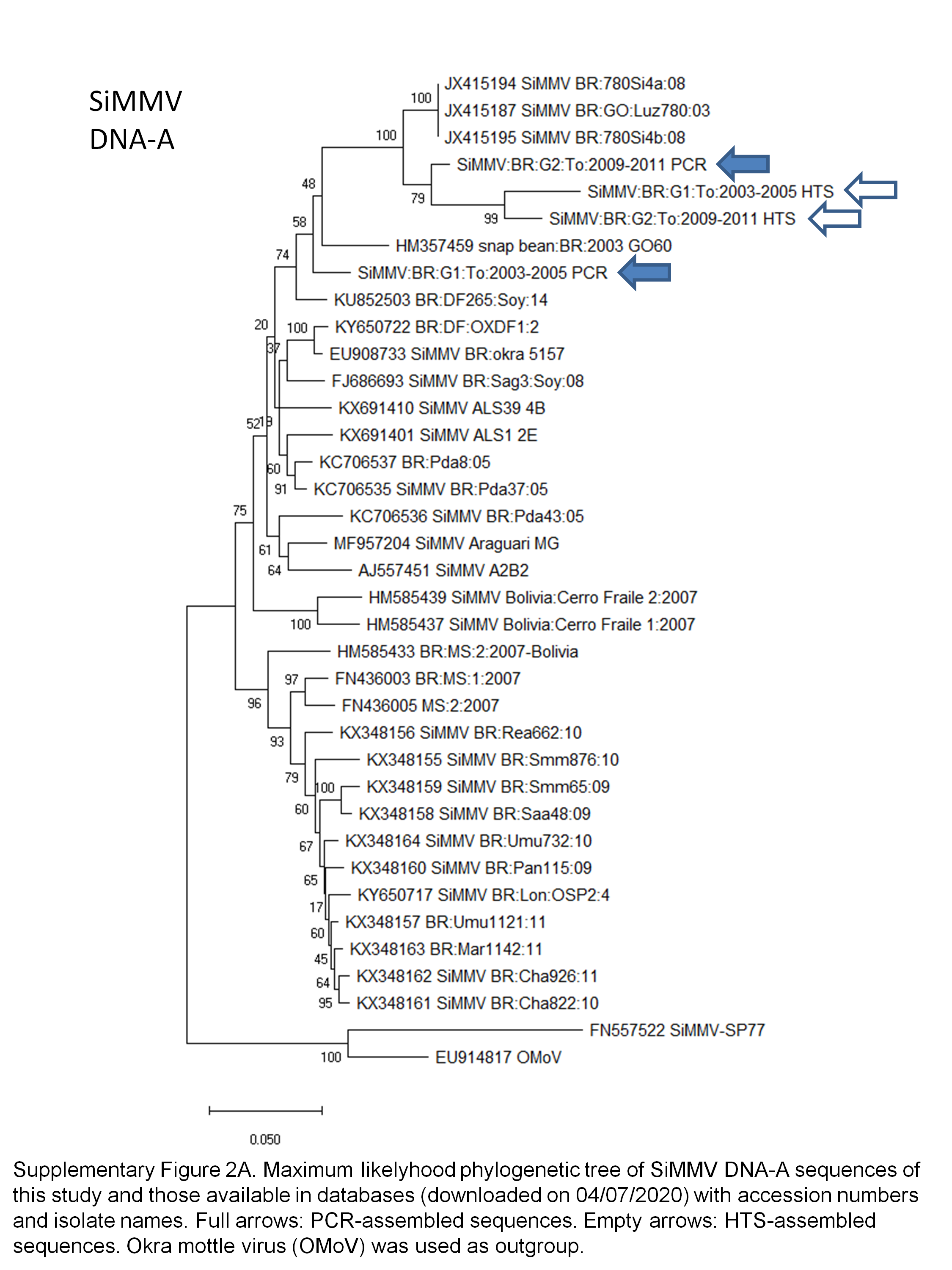

Supplement: Supplementary file 2 [file Image_2.tif]

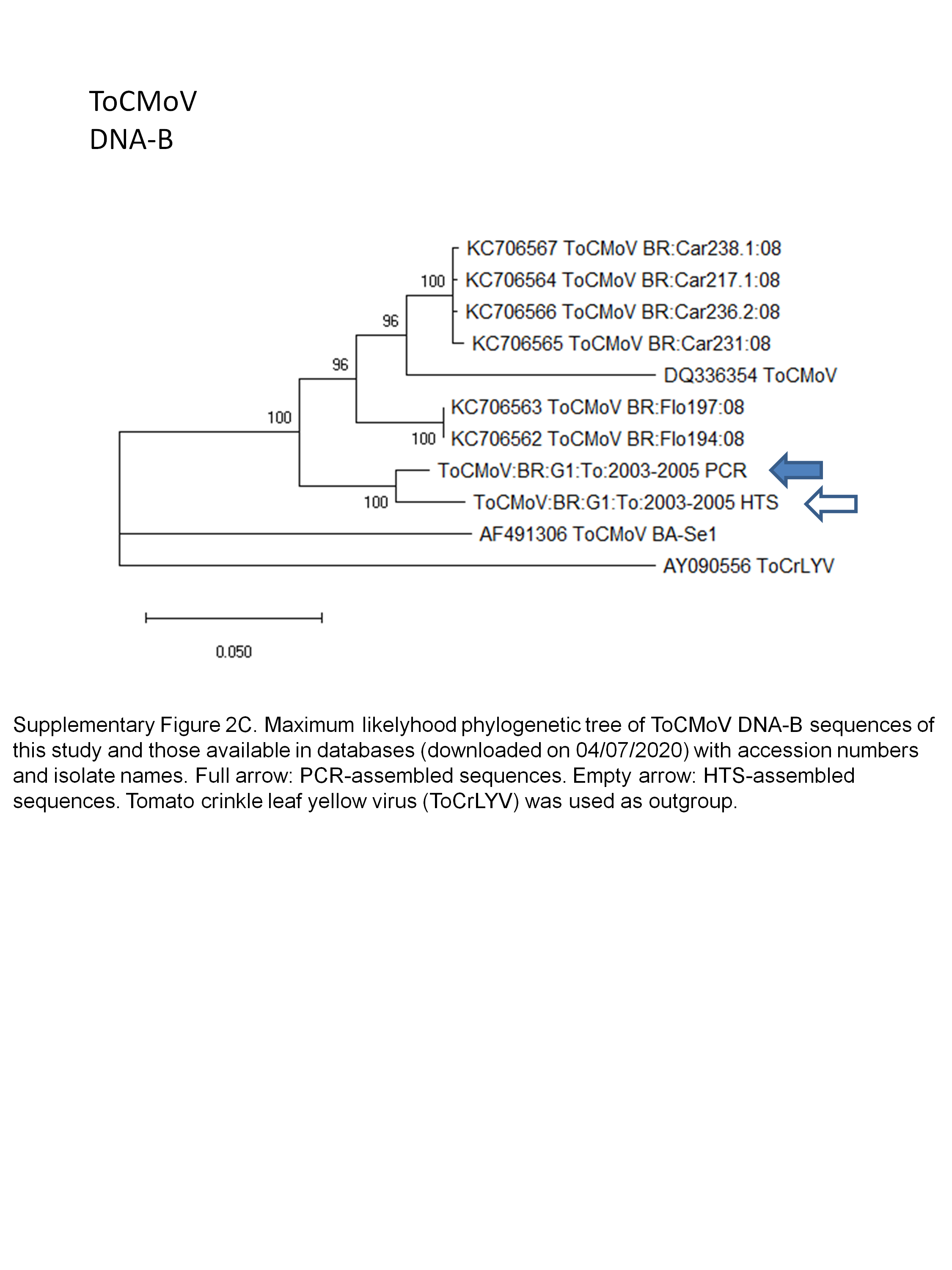

Supplement: Supplementary file 3 [file Image_3.tif]

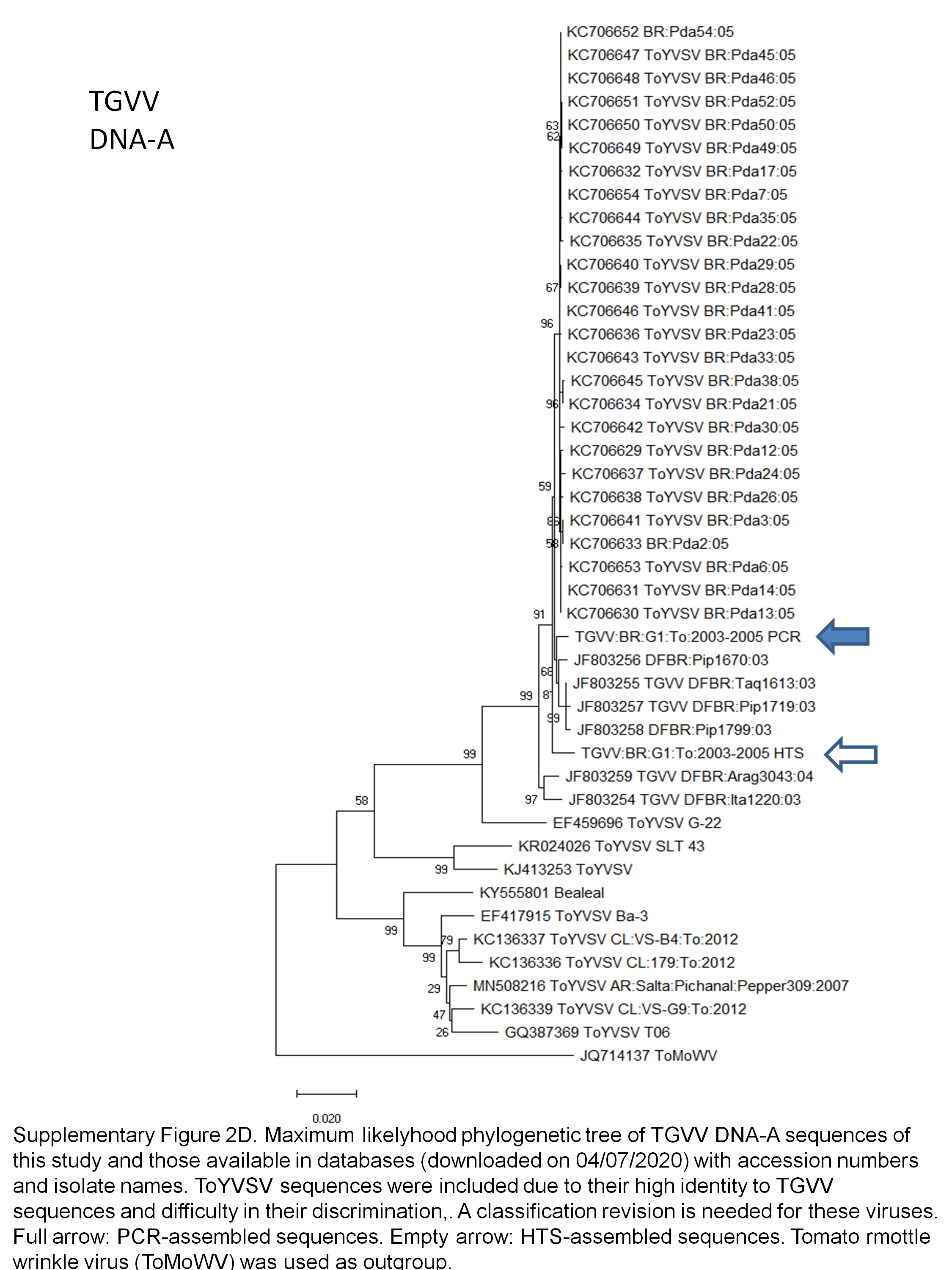

Supplement: Supplementary file 4 [file Image_4.tif]

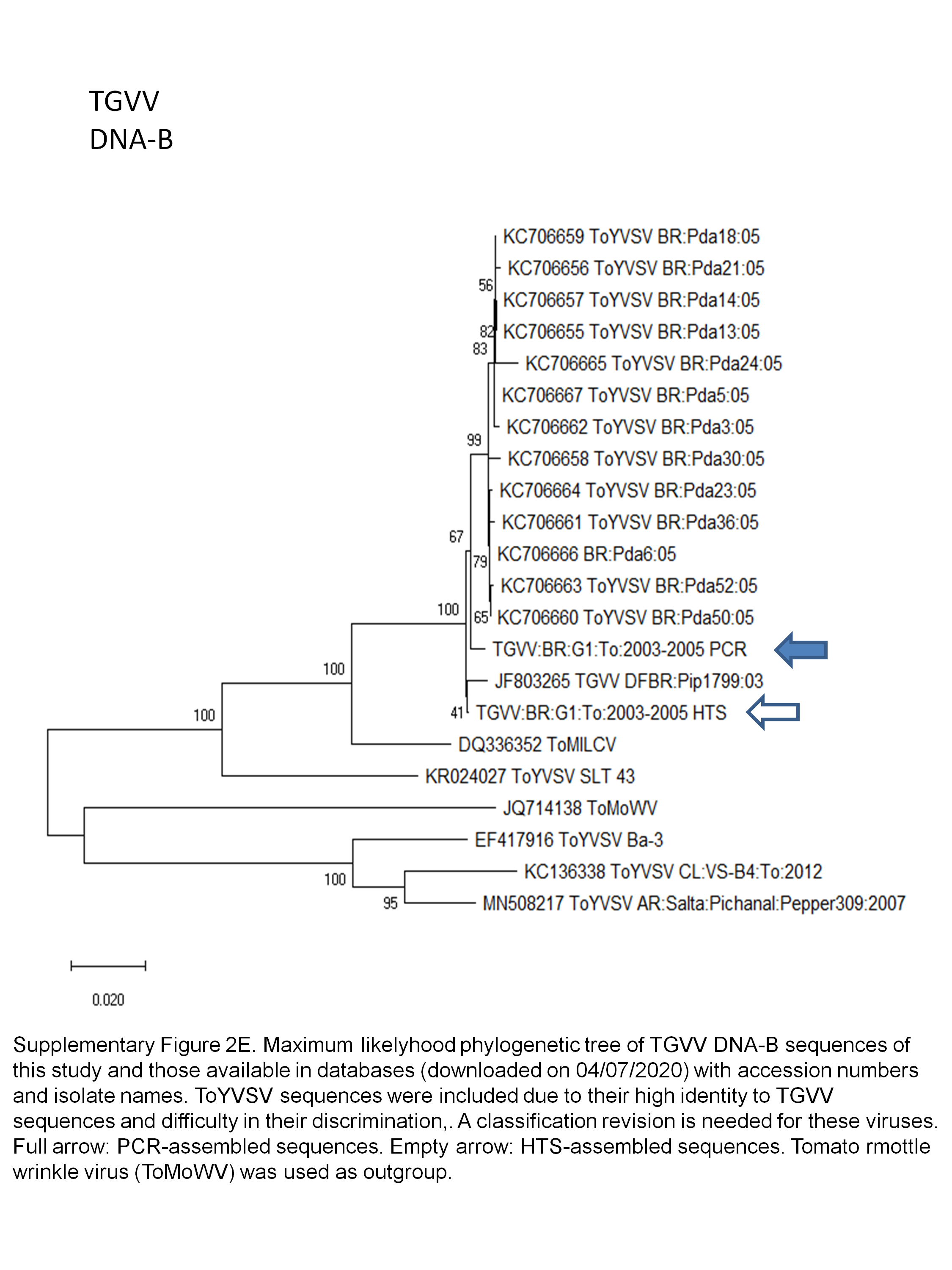

Supplement: Supplementary file 5 [file Image_5.tif]

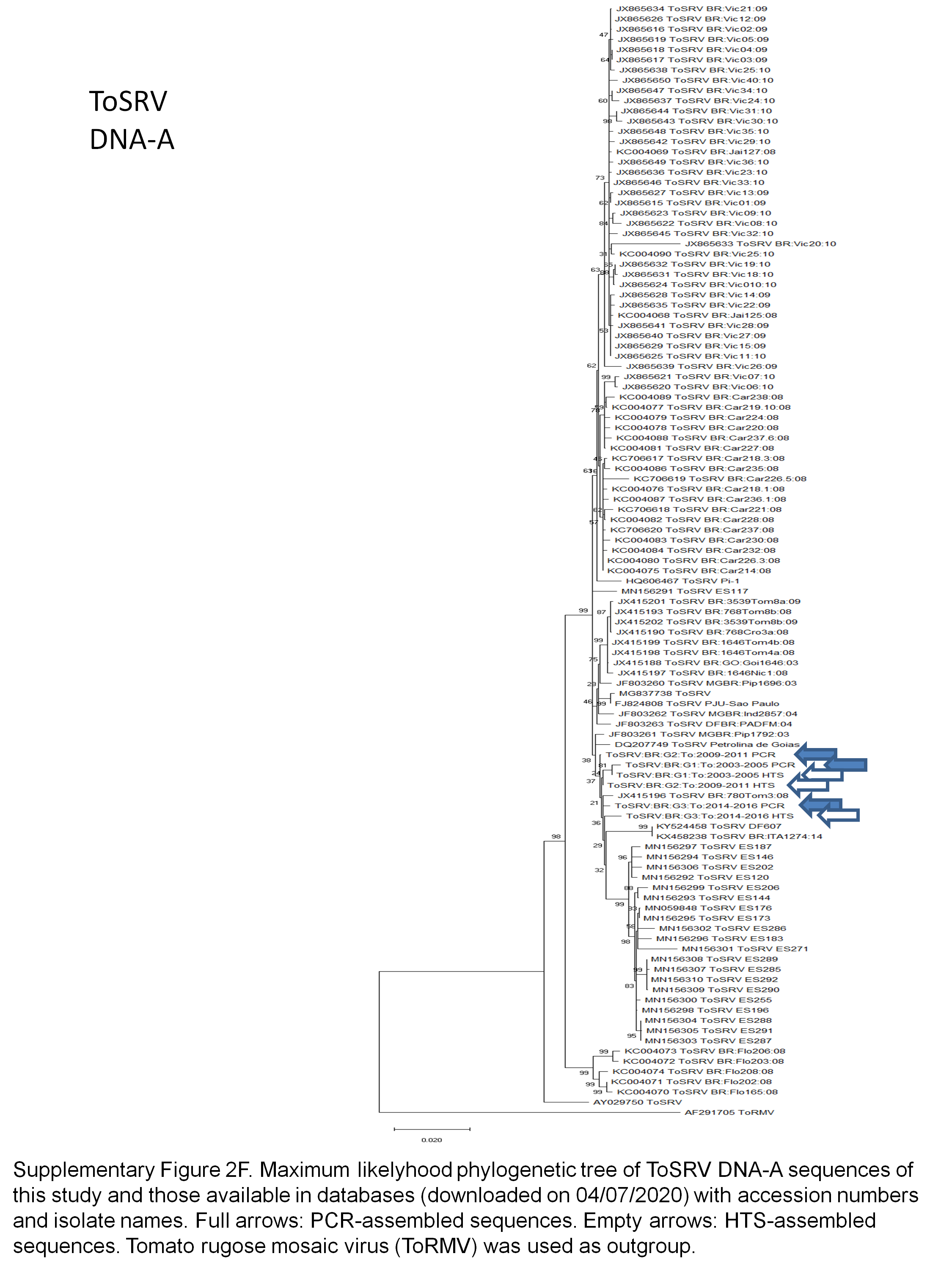

Supplement: Supplementary file 6 [file Image_6.tif]

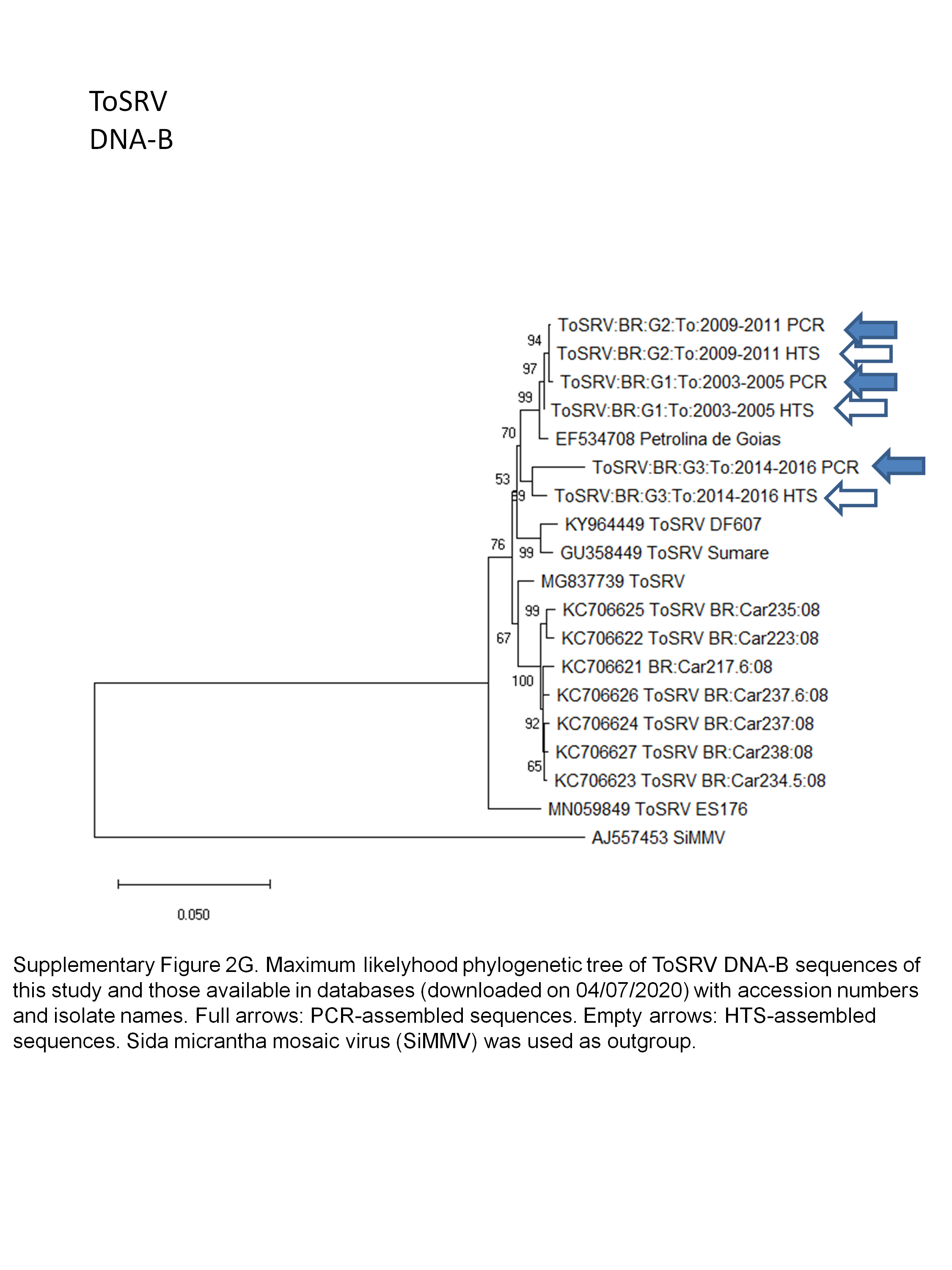

Supplement: Supplementary file 7 [file Image_7.tif]

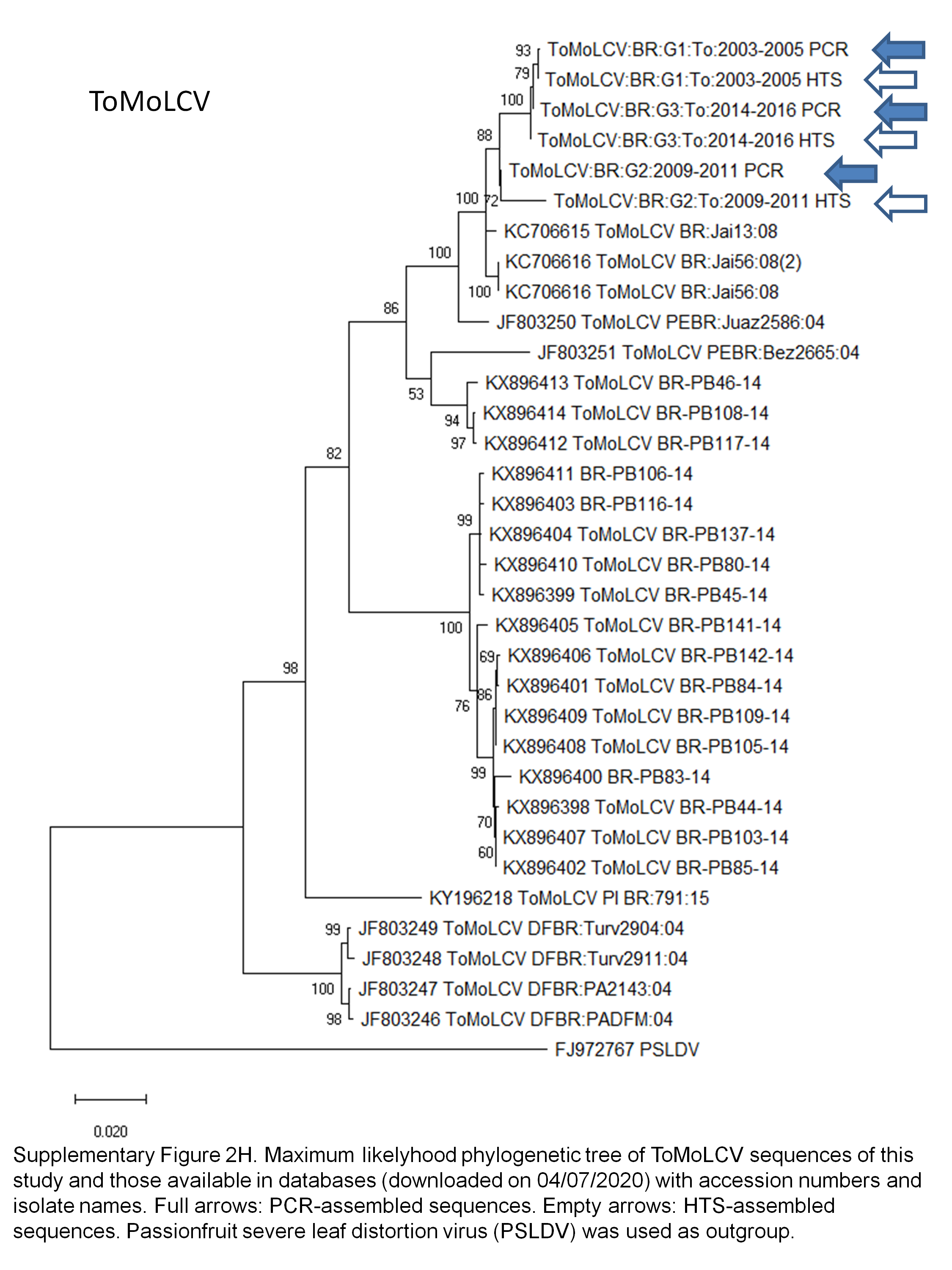

Supplement: Supplementary file 8 [file Image_8.tif]

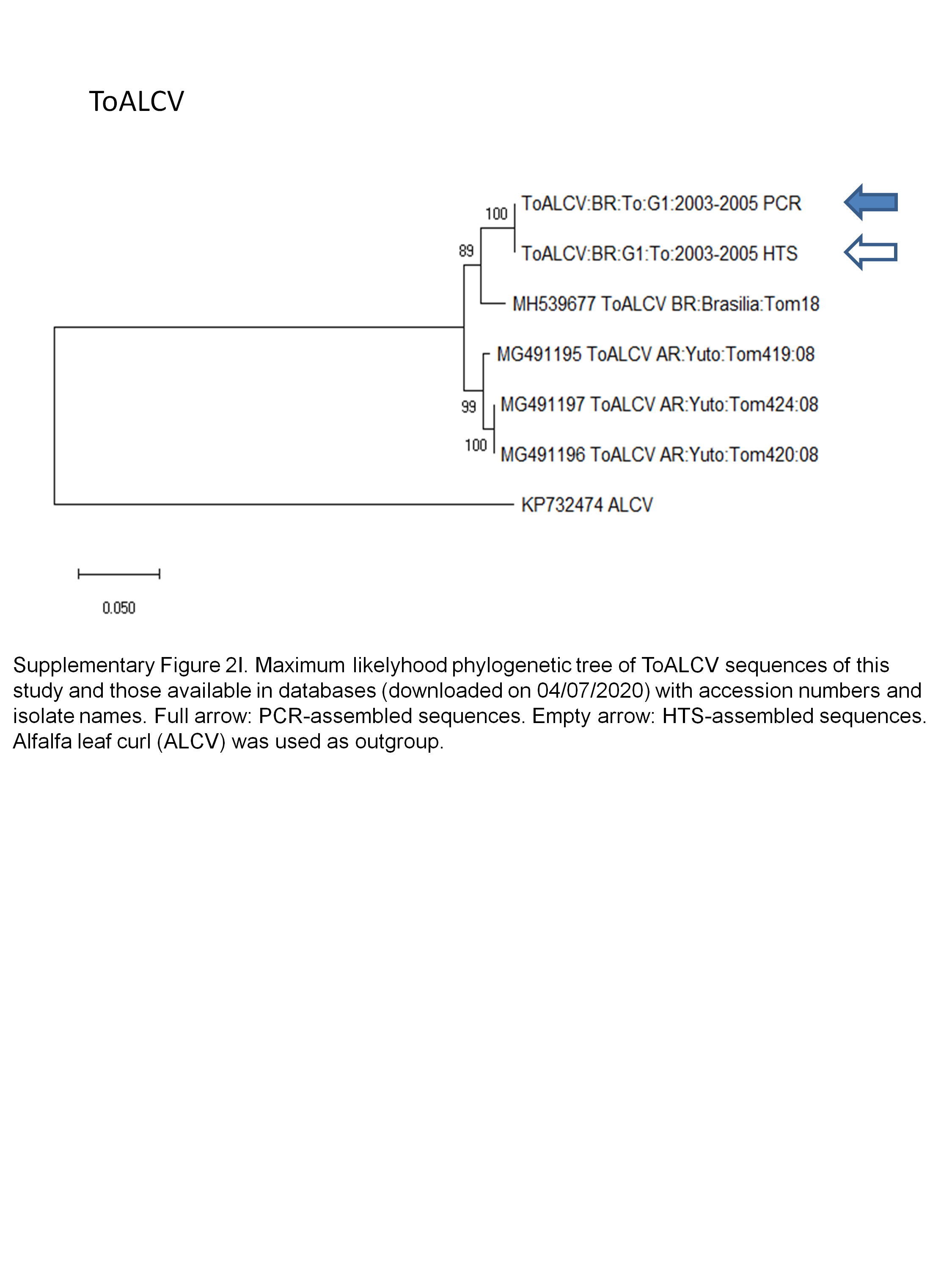

Supplement: Supplementary file 9 [file Image_9.tif]

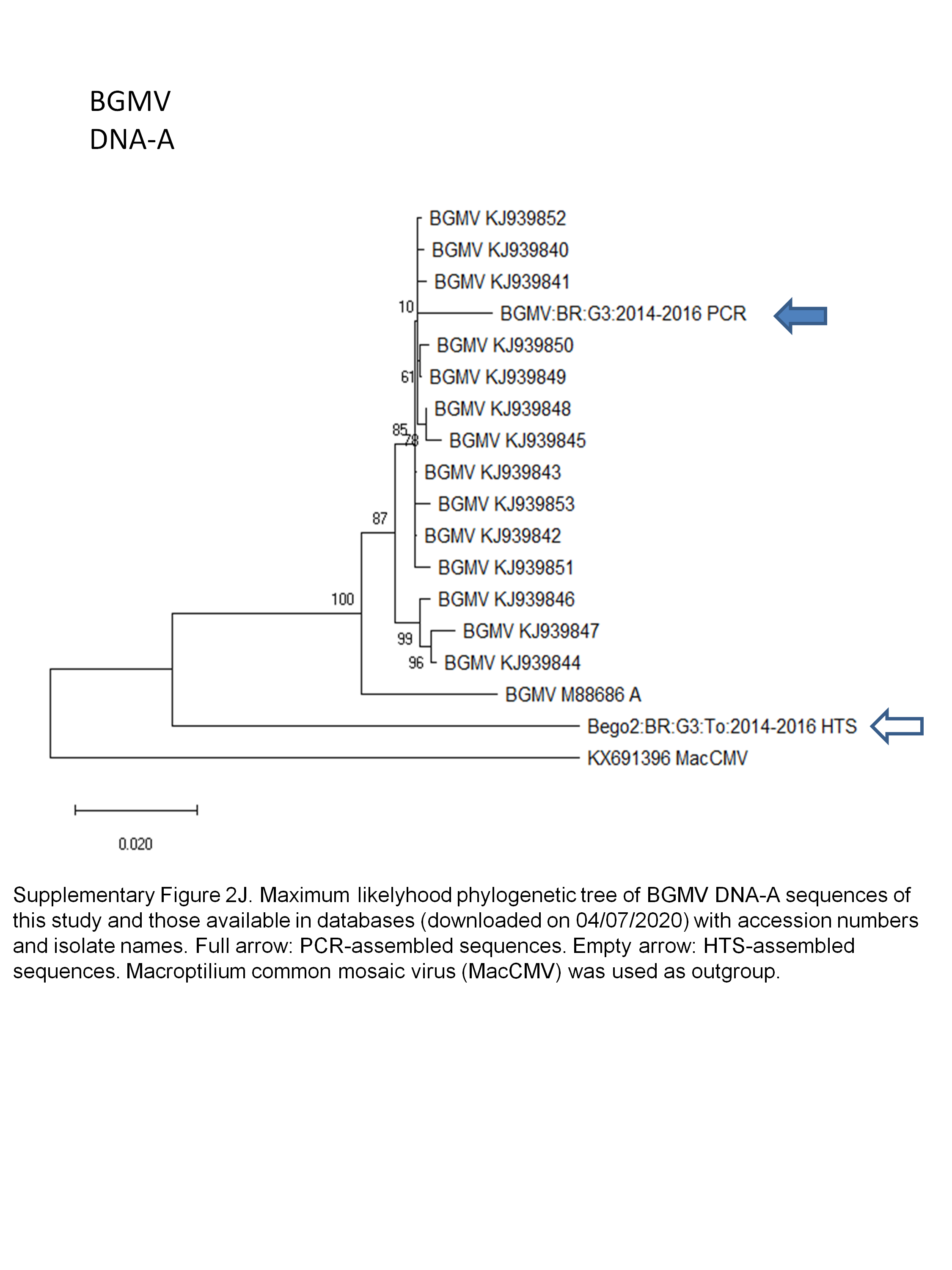

Supplement: Supplementary file 10 [file Image_10.tif]

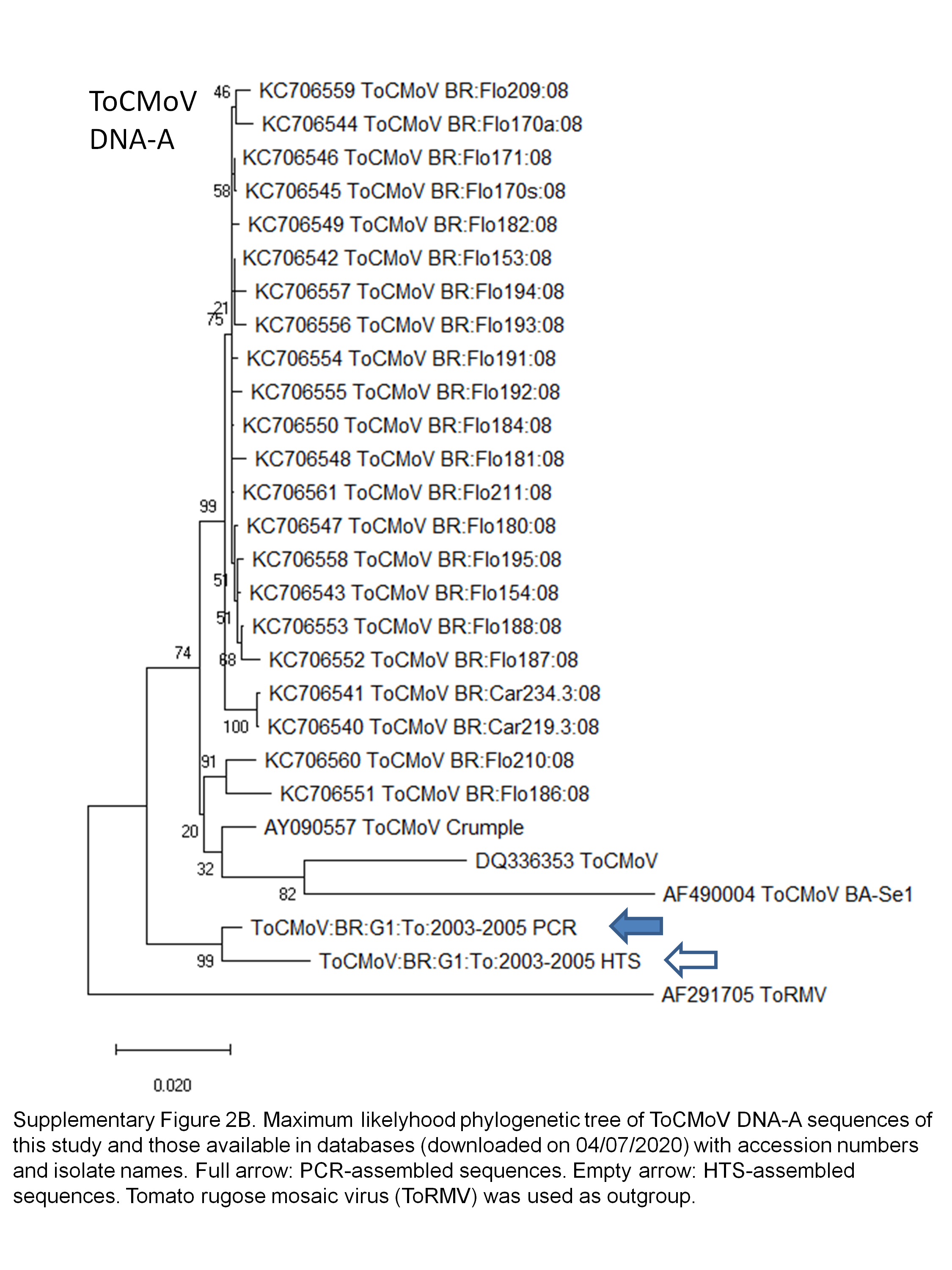

Supplement: Supplementary file 11 [file Image_11.tif]
