# Supplementary material for: A Temporal Diversity Analysis of Brazilian Begomoviruses in Tomato Reveals a Decrease in Species Richness between 2003 and 2016
Source: Front Plant Sci. 2020 Aug 6;11:1201. doi: 10.3389/fpls.2020.01201 (PMC7424291; doi:10.3389/fpls.2020.01201)
Supplement: Supplementary file 12 [file Table_1.docx]

Supplementary Table 1. Localization of the sampling areas of tomato plants infected by geminiviruses

| Group | Field | Coordinates | No. samples |
| --- | --- | --- | --- |
| 1 | 1 | 15°38'51.9"S 47°30'51.5"W | 30 |
|  | 2 | 15°39’30.83”S 47°32’3.14”W | 2 |
|  | 3 | 15°39’25.35”S 47°32’1.36”W | 3 |
|  | 4 | 15°31’21.0”S 47°29’43.6”W | 8 |
|  | 5 | 15°37'55.4"S 47°32'57.7"W | 11 |
|  | 6 | 15°38’5.23”S 47°31’56.0”W | 21 |
|  | 7 | 15°35'36.3"S 47°32'11.4"W | 18 |
|  | 8 | 15°33'37.7"S 47°32'28.5"W | 10 |
|  | 9 | 15°33'53.7"S 47°31'55.3"W | 4 |
|  | **Total** |  | **107** |
| 2 | 1 | 15°39'07.5"S, 47°31'11.8"W | 28 |
|  | 2 | 15°37'08.3"S 47°31'56.3"W | 22 |
|  | 3 | 15°43'10.380"S 47°19'22.994"W | 4 |
|  | 4 | 15°39’01.7”S 47°30’41.8”W | 28 |
|  | 5 | 15°31'50.2"S, 47° 29'26.9"W | 14 |
|  | 6 | 15°39’33.642S 47°32’4.636”W | 2 |
|  | 7 | 15°37'08.4"S 47°32'02.9"W | 7 |
|  | 8 | 15°31'53.335"S 47°29'12.473"W | 1 |
|  | 9 | 15°37'05.1"S 47°31'59.7"W | 7 |
|  | 10 | 15° 37’34.2”S, 47° 32’07.6”W | 5 |
|  | **Total** |  | **118** |
| 3 | 1 | 15°39'16.8”S 47°31’27.4”W | 12 |
|  | 2 | 15°38’5.23”S, 47°31’56.70”W | 16 |
|  | 3 | 15°39'07.5”S 47°31’11.8”W | 25 |
|  | 4 | 15°39’33.6”S 47°32’4.6”W | 8 |
|  | 5 | 15°40'04.3"S 47°24'19.8"W | 5 |
|  | 6 | 15°39’26.3”S, 47°32’00.1”W | 47 |
|  | 7 | 15°32’49.9”S, 47°26’46.7”W | 1 |
|  | 8 | 15°33'25.4"S 47°33'02.9"W | 2 |
|  | 9 | 15°41'59.813"S 47°23'9.254"W | 13 |
|  | **Total** |  | **129** |
