# Supplementary material for: A Temporal Diversity Analysis of Brazilian Begomoviruses in Tomato Reveals a Decrease in Species Richness between 2003 and 2016
Source: Front Plant Sci. 2020 Aug 6;11:1201. doi: 10.3389/fpls.2020.01201 (PMC7424291; doi:10.3389/fpls.2020.01201)
Supplement: Supplementary file 13 [file Table_2.docx]

**Supplementary Table 2.** Specific primer sets used for detection and genome-wide amplification of geminivirus components DNA-A and DNA-B

| Component | Name | Sequence (5’-3’) | Virus^1^ | Amplicon (kbp) |
| --- | --- | --- | --- | --- |
| DNA-A | TGVV1Fo | AAAGCSTTGGATAGATTTTC | TGVV (detection)^2^ | 0.5 |
|  | TGVV6Re | GAGCTATCGGGTCTCATC |  |  |
|  | TGVV144Fo | CTTTAATTCAAAATGCCAAAGCGA | TGVV (genome-wide amplification) | 2.6 |
|  | TGVV140Re | GTCGACCACTTACGAATGTC |  |  |
|  | SiMA1563Fo | AGTGTCCAGCCTCTCAATGC | SiMMV (detection) | 0.6 |
|  | SiMA2356Re | GACCTGGTCTCCCCAACAAG |  |  |
|  | SiMMV1001Fo | CCTGTGTATGCAACGCTTAAG | SiMMV (genome-wide amplification | 2.7 |
|  | SiMMV958Re | CATGCCATATACAGTAACAAAGCA |  |  |
|  | SiMMV81Fo | CGTGGAGCTCTGGTGTCCGC | SiMMV (genome-wide amplification | 2.7 |
|  | SiMMV70Re | GGGGGGGAGAGAGATCGCACG |  |  |
|  | ToMoLCVFo | TCCCAGAAGCTGTCGTTGAC | ToMoLCV (detection) | 0.8 |
|  | ToMoLCVRe | CGGAGGAGATGCAAGAGTGG |  |  |
|  | ToMoLCV72Fo | TCAATAAATGACAAATATGACCGT | ToMoLCV (genome-wide amplification | 2.6 |
|  | ToMoLCV67Re | CTGTACCACATAGTAAAAGACAAA |  |  |
|  | ToMoLCV2534Fo | ACTCTATGGCAATTGGTGTAT | ToMoLCV (genome-wide amplification | 2.6 |
|  | ToMoLCV2541Re | GCCATAGAGTTTTAGAGAGAACT |  |  |
|  | ToSRV-1F | GCTAAACATTCGGTTGATATACC | ToSRV (detection)^2^ | 0.9 |
|  | ToSRV-2R | CGTGTTGTTTAAGCGTTTCTGCG |  |  |
|  | ToSRV183Fo | TGATGCCCCATGGCGTTT | ToSRV (genome-wide amplification | 2.6 |
|  | ToSRV180Re | ACGCTTAGGCATTTTGAATTAAAGCAAA |  |  |
|  | ToSRV39Fo | ATTAAAGTAAAGTGATTGTCTGTG | ToSRV (genome-wide amplification | 2,6 |
|  | ToSRV45Re | CTTTAATTGAAACTAAAGGGGTG |  |  |
|  | ToCMoV1Fo | AGTGCTTCCATGACTGATCC | ToCMoV (detection) | 0.6 |
|  | ToCMoV4Re | ATGTGAAGGCCCAAAAAGAATCG |  |  |
|  | ToCMoV1427Fo | TAGCAGCCGATGTTGAACT | ToCMoV (genome-wide amplification | 2.6 |
|  | ToCMoV1443Re | ACTCCCCCCTCTATCAAAGTT |  |  |
|  | ToALCV340Pst_U2Fo^3^ | ACGCATTTCTGCAGACTTGCGCGGATCGA | ToALCV (detection) | 2.9 |
|  | ToALCV363Pst_U1Re^3^ | CCGCGCAAGTCTGCAGAAATGCGTTGTAAC |  |  |
|  | ToALCV340Pst_U2Fo^3^ | ACGCATTTCTGCAGACTTGCGCGGATCGA | ToALCV (genome-wide amplification | 2.9 |
|  | ToALCV_U2Re^3^ | GAACGATCGGGGAAATTCGAAAATGCGTTGTAACTTCTC |  |  |
|  | ToALCV601Fe | AAAAGCGAAGTACCCCAAACCG | ToALCV (genome-wide amplification | 2,9 |
|  | ToALCV443Re | GATAAAGTTCTTCCCACTCGA |  |  |
|  | ToML95Fo | CCAATCAGAAATGGTCCTCAA | Bego1:BR:G1 (detection) | 2.6 |
|  | ToML100Re | GATTGGACGGTCATATTTGTC |  |  |
|  | BGMU1 Fo^3^ | ATGGTGGAGCACGACACTCGTTCAAAAGTCTCTATGAATCGG | Bego2:BR:G3 (detection and genome-wide amplification) | 2.9 |
|  | BGMU12528 Re^3^ | TTTTGAACGAGAGCTCAATCGGGGTACACC |  |  |
|  | ToRMV1028Fo | CCTAACTGATCAATGTACAACATAAG | ToRMV (detection) | 2.9 |
|  | ToRMV1031Re | AGGCGTTATTTCCATTAATAATGTAAT |  |  |
| DNA-B | ToSRV38F | GCGAAATCGTGTACGTTGCC | ToSRV (detection) | 0.3 |
|  | ToSRV329R | ACCCACACGAAAGCAGAGTT |  |  |
|  | ToSRV336Fo | ACGTCTATACATATTGTCCAGG | ToSRV (genome-wide amplification | 2.6 |
|  | ToSRV317Re | CAGAGTTTAAATATGCATTCATTTGTC |  |  |
|  | ToSRV1242Fe | TTGTTCAGTAATAATGTTCAGCTC | ToSRV (genome-wide amplification | 2.6 |
|  | ToSRV1244Re | AGTACTTGTATTTATTATTACAGCGAG |  |  |
|  | SiMMV46F | GTGCCCGTTGGATACCTCTT | SiMMV (detection) | 0.9 |
|  | SiMMV923R | TCCTTCGACCTTTGGGGTGG |  |  |
|  | ToCMoV177Fo | CGGAACTTTATCGAATTTCGCAT | ToCMoV (detection and genome-wide amplification) | 2.6 |
|  | ToCMoVRe | TCAACGGTCTTAGATATATGGACG |  |  |
|  | TGVV509Fo | TGAGAACCAGTTGGTCCG | TGVV (detection and genome-wide amplification) | 2.6 |
|  | TGVV495Re | GCGACAACTTAATATCCTCATTGAC |  |  |
|  | TGVV134Fo | GAAATAAATTACAGCTTTTACGGTA | TGVV (detection and genome-wide amplification) | 2.6 |
|  | TGVVRe | ATTAAAGTTACTCCACTAACTATATTTC |  |  |

^1^TGVV: tomato golden vein virus; SiMMV: sida micrantha mosaic virus; ToMoLCV: tomato mottle leaf curl virus; ToSRV: tomato severe rugose virus; ToCMoV: tomato chlorotic mottle virus; ToALCV: tomato apical leaf curl virus; Bego1:BR:G1 tomato begomovirus-1; Bego2:BR:G3 tomato begomovirus-2; ToRMV tomato rugose mosaic virus

^2^ToSRV specific primers by Fernandes, F.R., Albuquerque, L.C., Inoue-Nagata, A.K. (2010). Development of a species-specific detection method for three Brazilian tomato begomoviruses. Tropical Plant Pathology 35, 43-47. doi: [10.1590/S1982-56762010000100007](https://doi.org/10.1590/S1982-56762010000100007)

^3^Long primers designed for Gibson assembly to produce an infectious clone
