# Supplementary material for: A Temporal Diversity Analysis of Brazilian Begomoviruses in Tomato Reveals a Decrease in Species Richness between 2003 and 2016
Source: Front Plant Sci. 2020 Aug 6;11:1201. doi: 10.3389/fpls.2020.01201 (PMC7424291; doi:10.3389/fpls.2020.01201)
Supplement: Supplementary file 14 [file Table_3.docx]

Supplementary Table 3. High-Throughput Sequencing (HTS) data summary of reads and contigs mapping to geminivirus sequences dataset.

| Samples | Total reads | Trimmed | Reads Q20 (%)^1^ | Reads Q30 (%)^2^ | Velvet | MEGAHIT |
| --- | --- | --- | --- | --- | --- | --- |
| 2003-2005 | 25,522,962 | 23,894,300 | 95.5 | 92.8 | 1570 | 430 |
| 2009-2011 | 21,442,638 | 20,105,579 | 95.7 | 93.1 | 1522 | 1205 |
| 2014-2016 | 19,960,206 | 18,869,564 | 96.1 | 93.6 | 1436 | 1139 |

^1^ Q20(%): ratio of reads with Phred Quality Score greater than 20.

^2^ Q30(%):ratio of reads with Phred Quality Score greater than 30.
