# Supplementary material for: A Temporal Diversity Analysis of Brazilian Begomoviruses in Tomato Reveals a Decrease in Species Richness between 2003 and 2016
Source: Front Plant Sci. 2020 Aug 6;11:1201. doi: 10.3389/fpls.2020.01201 (PMC7424291; doi:10.3389/fpls.2020.01201)
Supplement: Supplementary file 15 [file Table_4.docx]

Supplementary Table 6. HTS-assembled geminivirus sequences in each group (G)

| G | Fasta sequence |
| --- | --- |
| 1 | >Sida micrantha mosaic virus SiMMV:BR:G1:To:2003-2005 segment DNA-A, complete sequence  ACCGGATGGCCGCGCGATTTTTTCCCCCCCCCCACGTGGCGCGCTGGTGGTCGTGCGATCTCTCTCCCCCTCTCTCGCGCGACGTGGAGCTCTGGTGTCCGCGCGTTCCCCCCCGCGCGTTCTGCCTTTAATTTAAATTAAAGGAAATAACTTTCATCAGGACCAATGAAATTGCGTCTTTTAAGCCTAGATATTTGCGAAAGACTTGGGCCCTAAGTTGTTTGACAGCTATAAAATTAAGTCATGCATGACGTCAGTAATTATTTCGAAATGCCCAAGCGGGATCCCTCATGGCGCCAGATGGCGGGAACCTCAAAGGTTAGCCGCTCTACCAACTTTTCCCCTCGTGGAGGAGGAGGCCCAAAATACAACAAGGCCTCGGAATGGGTTCACAGGCCCATGTACAGGAAGCCCAGGATATACAGGATGATAAGGACTCCTGATGTTCCAAGAGGCTGTGAAGGGCCCTGTAAGGTCCAGTCCTACGAGCAGCGTCACGATATCTCACATGTCGGCAAGGTCATGTGCATATCTGATGTCACACGTGGCAACGGTATAACCCACCGTGTCGGTAAGCGTTTCTGCGTTAAGTCTGTGTACATTTTAGGGAAGATATGGATGGACGAGAACATCAAGCTCAAGAACCACACGAACAGTGTCATGTTCTGGTTGGTCAGAGACCGTAGACCGTATGGTACTCCTATGGATTTTGGCCAGGTGTTCAACATGTTTGACAACGAGCCTAGCACTGCAACTGTTAAGAACGATCTTCGGGATCGTTTTCAGGTCATGCACAAGTTCTACGCCAAGGTCACTGGTGGTCAATATGCCAGCAACGAGCAGGCTCTGGTCAAGAGGTTCTGGAAGGTCAACAATCATGTGGTGTACAACCACCAGGAAGCTGGCAAGTATGAGAATCACACGGAGAATGCTTTGTTACTGTATATGGCATGTACTCATGCCTCTAACCCTGTGTATGCAACGCTTAAGATTCGGATCTACTTCTACGATTCGATAACCAATTAATAAAATTTGAATTTTATATCATGATCTTCAAGTACATAGTTTACATAGGCTTTGTCAGTGGCAAAGCGAACAGCTCTAATTACATTGTTAAGCGTGATTACGCCTAATTGATCTAAATACATCATAATTAAATATTTAAATCTATTTAAATATGTCATCCCAGAAGCTCTCAGGGATGTCGTCCAGATTTGGAAGTTGAGATAAGCCTTGTGGAGACCCAGTACTCTCCTCAGGTTGTGGTTGAACCGTATTTGTACGTGGTAGACTCTGGTCCTCGTGTATAATATGTCCTCGACGTTGTACATCTTGAAATAAAGGGGATTTGATATCTCCCAAATATAAACGCCATTCTCTGCCTGACGTGCAGTGATGAGTTCCCCTGTGCGTGAATCCATGTCCTCTGCAGTCGATGTGTACGTAAATAGAGCACCCGCACTCTATATCAATTCGTCGTCTCCTGATTCCTCGTTTTTTAGCAATCCTGTGTCTCGGTTTGATAGAGGGGGGCGTCGAGGAAGATGAATTTAGCATTGTGTAGTGTCCAGCCTCTCAATGCCGCATTTTCCTCTTTGTCTAGGAAGTCTTTATAGCTGGCCCCCTCTCCAGGATTGCACAACACGATTGAAGGGACCCCACCTTCAATCAAACGAGGCTTTCCGTACTTGCAGTTTGTCTGCCATTTCTGTTGGGCCCCTATTAGCTCTTTCCAGTGTTTCAACTTTAGGTAATGCGGAGCGACGTCATCAATGACGTTATACTCCACTTGATCAGAATATACCTTTGAGTTGAAATCCAGATGGCCACTGAAGTAATTATGTGGGCCTAATGCTCTAGCCCACATCGTCTTGCCCGTTCTGCAATCACCTTCAATGATGATACTAATAGGTCGTTCCGGCCGCGCAGCGGCACCTCTCCCAAAATAGTCATCTGCCCAAGCTTGCATCTCTCTCGGTACGCTAGTGAAAGAGGAGAGGGGAAACGTAGGGGCCCATGGCTCCGGAGCCCTTGTAAATATCCTATCTAAATTACTATTTAGATTGTGAAACTGAAATAAGAACTTTTCAGGCAGCTTCTCACGGATTATCTGCAGGGCGACGTCTTTGGAAGGTGCGTTCAAGGCTTCTGCGGCAGCGTCGTTAGTTGTCTGGCAACCGCCTCTAGCACTTCTTCCGTCGATTTGGAATTCCCCCCACTCGATAGTATCTCCGTCCTTGTCGACATAGGATTTGACGTCGGACGATGACTTAGCTCTCTGAATGTTCGGATGGAAATGTGTTGACCTTGTTGGGGAGACCAGGTCGAAGAATCGTTGATTTTGGCAGCAGTAGTTTCCCTCAAATTGAAGAAGCACGTGGAGATGAGGCTCCCCATTTTCATGGAGCTCTCTGCAAACCTTGATGAACTTCTTATTTGTAGGAGTGTTTAGGGTTTTTAATTGGGAAAGTGCTTCTTCTTTCGATAATGAGCATTTGGGATATGTGAGGAAATAATTCTTCGCTTTTATTTGAAAGCGCTTTGGAGCTGATGGCATATTTGTAAATATGACCCTTACTACCAATTGGTAGCTGCTCTAAAACTCATATGAATTGGTAGTTATGGTAGCTCTTATATAGTAGAAGTTCTTAAGGGCCTTAAGGGCCTAAAGCGGCCATCCGCACTAATATT |
| 1 | >Tomato chlorotic mottle virus ToCMoV:BR:G1:To:2003-2005 segment DNA-A, complete sequence  ACCGGATGGCCGCGCGATTTTTTTAAGGCCCATTTATGTTGGGCCTCTCTTTTGGGCCTTCACCTTTAATTTTATTGAAAGTAAATGTGGCCCAATCATGATGCATTTGACGAGCTAAGATATTTACAACTACTTAACGCCCAAGTTGCATAACGGCTATAAATTAAACGTGCATATGTCGATAACTTTAATTCAAAATGCCTAAGCGCGATGCCCCATGGCGCCACATGGCTAGTACATCTAAGATTAGCCGGGGCGTTAATAATTCTCCTCGAGCAGGAGTTGGGCTACGGTCCAACAAGGCCTCTGACTGGGTAAACAGGCCCATGTACAGGAAGCCCAAGATATATCGGATGTATAGAACCCCCGATGTTCCAAGGGGTTGTGAAGGCCCATGTAAAGTGCAATCGTTTGAACAGCGTCACGACATTTCCCATACTGGTAAGGTGATGTGTGTTTCCGATGTGACACGTGGTAACGGTATTACACACCGTGTGGGTAAGCGTTTCTGTGTTAAGTCTGTGTATATTCTGGGTAAGATATGGATGGATGAGAACATCAAGTTGAAGAACCACACGAACAGTGCCATGTTCTGGTTGGTCAGGGACCGTAGACCGTATGGCACCCCTATGGACTTTGGCCAGGTGTTTAACATGTTCGATAACGAGCCTAGCACCGCCACTGTGAAAAACGATCTCCGTGATCGTTTTCAAGTTATGCACAAGTTCTATGCTAAGGTCACAGGTGGACAATACGCAAGTAACGAGCAGGCGCTTGTCAAGCGTTTCTGGAAGGTCAACAACCATGTCGTTTACAACCATCAAGAGGCAGGGAAATACGAGAATCATACTGAGAACGCCTTGCTATTGTATATGGCATGTACTCATGCCTCTAACCCCGTGTATGCTACATTGAAAATTCGGATCTATTTTTATGATTCGATTACTAATTAATAAAATTTAAATTTTATTGAATGATTTTCGAGTACAGCATTTACATATGATTTGTCTGTTGCGAAACGAACAGCTCTGATTACATTATTAATGGAAATAACGCCTAACTGATCAATGTACAACATAATTAAATATTTAAATCTATTTAAATATGTCATCCCAGAAGCTCTCAGGGATGTCGTCCAGATTTGGAAGTTGAGATAAGCCTTGTGGAGACCCAGTACTCTCCTCAGGTTGTGGTTGAACCGTATTTGTACGTGGTAGACTCTGGTCCTCGTGTATAATATGTCCTCGACGTTGTACATCTTGAAATAAAGGGGATTTGATATTTCCCAAATATAAACGCCATTCTCTGCCTGACGTGCAGTGATGAGTTCCCCTGTGCGTGAATCCATGTCCTCTGCAGTCTATGTGTACGAAAATGGAGCAGCCGCACTGTAAATCAATTCGTCTTCTTCGTATAGCTCTCCTCTTAGCAGCCCGATGTTGAACTTTGATAGAGGGGGGAGTTGAGGAAGATGAATTGTGCATTGTGCTTTGTCCAACTACTTAATGCTGAGTTTTCCTCTTTGTCGAGGAAACATTTATAGCTGGCCCCCTCGCCAGGATTGCAAAGCACGATGCATGGGATACCACCTTTAATTTGAACTGGCTTTCCGTATTTGCAGTTTGATTGCCAATCCTTTTGGGCCCCAAGCAATTCTTTCCAGTGCTTTAACTTTAGATAGTGCGGTGCGATGTCATCAATGACGTTATATTCAACATGATTTGAATAAACCCTAGGATTGAAATCGAGGTGACCACTCAAGTAATTATGGGACCCTAATGCACGTGCCCACATTGTTTTGCCACTTCGAGAATCACCCTCGATTATTATACTGATAGGTCTCTCCGGCCGCGCAGCGGCATCTCTCCCGAAATAGTCATCTACCCAATCTTGCATCTCGACAGGCACGTTAGTGAACGACGAGAGTGGAAACGGAGGAACCCAAGGTTCTGGAGCCTTTGCGAAAATACGCTCTAAATTTGACCTAATGTTATGATGTTGAAGCACGAAATCTTTGGGTTGTTCTTCTTTTAATATATTGAGTGCTTCCATGACTGATCCTGCATTGAGAACCTTGGCGTACGTGTCGTTTGCAGATTGCTGACCTCCTCTAGCTGATCTGCCATCGATCTGGAAAACTCCATGATCAACGAAGTCTCCGTCTTTTTCCACGTATGATTTGACGTCTGACGAGCTCTTAGCTCCCTGTATGTTTGGATGGAAATGTGCTGACCTACTTGGGGAGACGAGGTCGAAGAACTTGTTGTTTTTGCAGTTGTATTTACCTTCGAATTGGATGAGCACGTGGAGATGAGGTTGCCCATCTTCGTGTAGTTCTCTCGAGACACGAATGAACAATTTATTAGTTGGTGTGTTTAGTGCTAATAATTGGGAAAGTGCTTCTTCTTTGGAAAGAGAACAGTGTGGATATGTTAGAAAATAATTTTTGGAATTTACTCGGAAATGTCTGGGCGGTGGCATATTTGTAATAATAGTTGGGCACCGATTGGGGTCTTCTCAAACTTACTAAAGCAATTGGGGACTGGGGTCTTATATATACTAGAACCCTCTATAGAACTTTCAATCTCGTTCACACACGTGGCGGCCATCCGATATAATATT |
| 1 | >Tomato chlorotic mottle virus ToCMoV:BR:G1:To:2003-2005 segment DNA-B, complete sequence  ACCGGATGGCCGCGCGATTTTTTTAAGGCCCATTTATGTTGGGCCTCTCTTTTGGGCCTTCACCTTTAATTTTATTGAAAGTAAATTTTATTTGAGTTCCGCTTTCGGATGGCCGCCACGTCCATATATCTAAGACCGTTGAATTAAATATGATAAGCGGAACTTTATCGAATTTCGCATGGTGTTTTTTATTGGTTATTAATATTGAAATTCGACCGTTAGATTGCACTTGATATTGAATATCGACCATCGTTCTCGAATAATGTGTCATTTGTACAAGTATTTGTTATGTGTGCAATCATACAACATACGTAACGTGGACCAATTAAATATTGACAGTATAACCAAAATAACCCATTGATGCAATTACAGCTATCTATATAATGTGATTGATATTGATATTTTTAAAACATCCACTAAGAATTTTGAACATGTATTTTAATAGTAATAAGCGTCGTTGGTCGTCCATCAACCGTCGTAATAACGTACGATATTCTACGTTTAAAAGGTCACATGCTGTGGTACGCAATGATGGGAAACGTCGATTGGGTATTTCAAACAAGTCCCTCGACGATACTAAGATGTCTTCACATAGGATTCATGAAAACCAATATGGGCCTGACTTTGTATTGGGCCATAACACAGCGTTATCCACTTTTATAACTTACCCCTCTCTTGGTAAAAGTGAGCCCAACCGTATTAAGTCATACATTAAGTTGAAACGTCTGCGCTTTAAAGGTACTGTTAAGATTGAACGTGTTCCTACCGATATGAACATGAATGGAATACCTGCTAAGATTGACGGGGTATTTTCTTTGGTTATTGTTGTCGATCGTAAACCTCATTTGAGTCCTTCTGGTAGTCTCTACACATTCGATGAGTTATTTGGTGCTAGGATCCATAGCCATGGAAATTTAGCGATAATCCCTTCTCTGAAGGATCGTTTTTACATACGTCATGTTGCTAAACGTGTCTTGTCAGTGGAGAAGGATAGTAATATGATTGACGTTGATGGGACGACAACACTCTCTAGTAGGCGTTATAATTGCTGGGCAAATTTTAGAGATTTCGATCATGAATCCTGTAACGGTGTATATGCTAACATAAGCAAGAACGCCTTACTAGTTTATTATTGTTGGATGTCGGAAACGGTATCCAAAGCATCTACATTTGTATCATTTGATCTTGATTATATTGGATAATTATATAGATCAATTTGCACATTATAAGTTGATAAGTTTAAATATGATAATAAATTGTCCTAGTGCTTTTATTAAATACTCAGAAAATCTTAAATTTATTTTAAAGACTTTGGTTCAGATGGAGTACAATTGGTATTAATACATTCTTGTACTGTTGACTTAACAATCTCATTTAATTGGGCCAAAGATAAGGTAATGTTGGACTGCGTTCTTTGCGCCGCAACGATTGACGCTGATTCTCCTGGGTCTAGAATTATCGTGTCCAATCTGTTCAGGTCCTTGTATGGATTTAGATCGTTGCCCCGTTCGGAGTCCACACATGAATTGCCAAGTCCAATTGTACTTCTGGAGGCCCACGACTCTCCAGGTTTAATTTCTATTGGGCCGTTAAGCCCAAATCTGGATGTCGAAGCGCATCTGACTAATTTCCTCTCCCATATCCCGTAACCCACATGAACAAAATCGATATCCTTATCTGAAAATTGCTTCGACAATATTTTAACTGTTGGTGCCCGGAAAGGGATATCTACTGAGTGTTTTGCTGTGGATAGTTTTAACTTGCCCTTGAACTTGGCGAAATGGGTCCTTTGGTGCACATTCGTATCGCTAACCCTGTAGTATAATTTCCATGGAATTGGGTCCTTCAGCGAAAAGAATGACGACGAGAAATAATGTAAGTCTATGTTACATCTAATTGGGAAGGTCCATGACGCTTGCAATGACTCGTTGTCCGTCATTCTCTTGTCATGTATTTCTACTATAACGGATCCTGTGGCGTTTATCGGGACTTGCTGTCTGTATTCGATGACACAATGATCTATTTTCATGCAACTCCGACTGAGTTTAGCACTAATTTGAGATGCCGTAGATGGAAATTGTAGAACAATTTCAGTTAGATCATGAGACAATTGATATTCATCTCTTTGAGACTCAACATAATTAAAAGCATTTGGAGGGATAACCAATTGAGAACTCATTATATTAAAAAAAGGCTGCGCAGCTGAATCGACAACTGGAATTAATAAGCGTAAATGATATGAACACACTAACAAGAATGTCAGAAGACAATATGTGTTGCGMTGTGACGATTAACGAAATATATTGTGGTAATGATGATAGTCAGTACTAACTGGCTTGTATTTATAGAGTGTGTATAGGAAAGAGAACAGTGTGGATATGTTAGAAAATAATTTTTGGAATTTACTCGGAAATGTCTGGGCGGTGGCATATTTGTAATAATAGTTGGGCACCGATTGGGGTCTTCTCAAACTTACTAAAGCAATTGGGGACTGGGGTCTTATATATACTAGAACCCTCTATAGAACTTTCAATCTCGTTCACACACGTGGCGGCCATCCGATATAATATT |
| 1 | >Tomato golden vein virus TGVV:BR:G1:To:2003-2005 segment DNA-A, complete sequence  ACCGGATGGCCGCGCGATTTTTTGTATGTGGTCCCACTGTGAGTGACCAATCAGATTGTGTCCTGGGCGGTTATTTATTTCGAAATACTTAGGCGCTAAGTTGTTAAAGTTGTATAAATTAGACATTCGTAAGTGGTCGACATACTTTAATTCAAAATGCCAAAGCGAGATGCCCCATGGCGTATAGTGGCAGGGACCACGAAAGTATCCCGCTCTTCTAATTATTCACCTCGGGGAGGTATACCCAAGCGGGATGCTTGGGTAAACAGGCCCATGTACAGGAAGCCCAGGATATATCGTACGTTGAGAGGGCCTGATGTTCCTAAAGGGTGTGAAGGCCCATGTAAAGTTCAATCCTATGAGCAACGTCATGACATTTCTCATCTTGGCAAGGTGATGTGTATCTCTGATGTGACACGTGGTAATGGTATTACACACCGTGTTGGTAAGCGTTTTTGTGTTAAGTCTGTGTACATATTGGGTAAGATATGGATGGACGAAAATATTAAACTGAAGAACCATACCAACAGTGTTATTTTTTGGTTAGTGAGGGATCGTAGACCCTATGGGACACCTATGGATTTTGGTCAGGTGTTTAACATGTTTGATAATGAGCCTAGTACTGCTACGGTGAAGAACGATCTTCGTGATCGTTATCAAGTCATTCATCGATTCAATGCTAAGGTTACGGGTGGACAGTATGCTAGCAACGAGCAAGCTCTTGTTCGGCGTTTCTGGAAGGTCAATAATCACGTGGTTTACAATCATCAGGAAGCAGGGAAATATGAGAATCATACGGAGAACGCCCTGTTATTGTATATGGCATGTACTCATGCCTCTAACCCCGTGTATGCTACTTTGAAAATTCGGATCTATTTTTATGATTCGATTACTAATTAATAAAATTTAAATTTTATTGAATGATTTTCGAGTACAGCATTTACATATGATTTGTCTGTTGCGAAACGAACAGCTCTGATTACATTATTAATGGAAATAACGCCTAATCGATCTAGATACAATAAGACTAAATATTTAAATCTAGTTAAATATGTCATCCCAGAAGCTCTCAGGGATGTCGTCCAGACTTGGAAATTTAGAAATGCCTTGTGGAGACCCAACGCTTTCCGCAGGTTGTGGTTGAAACGTATCTGCACATGGTATATCCTGGTCGTTGTGTAAGAGGGGTCCTCGACGTTGGTTATCTTGAAATAGAGGGGATTTGTAATCTCCCAAATAAAGACGCCATTCTCTGCTTGAGGCACAGTGATGGGTTCCGCGGTGCGTGAATCCATTATTTCTACAGTTGATGTGGATGTATATGGAGCAGCCGCAGTTTAGGTCTATGCGTTTACGCCGGGTTGTTTTTTGTTTGGCTGCTCTGTGCCGTGGCTTGATAGAGGGGGGAGTTGAGGAAGATGAATTTAGCATTATGCAGTGTCCAAGCTCTTAGAGATGCATTTTCATGTTTGTCAAGGAAGTCTTTATAACTAGCCCCCTCTCCTGGATTGCAAAGCACGATTGATGGGATCCCTCCTTTAATTTGAACCGGCTTTCCGTACTTGCAATTGCTTTGCCAGTCACGCTGGGACCCAATAAGTTCTTTCCAGTGCTTCATTTTTAGATATTGTGGAGTGACATCATCAATGACGTTATACTCCACTTCATTTGAATAAACTTTAGAATTAAAATCCAAGTGTCCACTCAAATAATTATGTGATCCTAGTGCACGAGCCCACATAGTCTTCCCCGTCCTGGAATCACCCTCAATGATGATACTAATAGGTCTTTCCGGCCGCGCAGCGGCACCCCTTCCGAAATACTCATCAGCCCATTGCTGCATCTCATCAGGAACGTGAGTAAAGGAAGATAATTCAAATAGAGGGACCCATGGGGCTGGAGCCTTTGCAAATATTTTTTGGACATGTGATTGTATTTTATCCAAATGAAGAACATAATCTCTAGGTTGTTCTTCTTTTAATATATTTAAGGCCTTGGATAGATTTTCTGCGTTTAGAACCTTTGCATAAGTGTCATTGGCAGTTTGGCAGCCTCCTCTTGCTGATCTTCCATCGATCTGGAAATTTCCAAAATCAATGAAGTCTCCGTCTTTTTCCATATAGGTCTTGACGTCGGACGAGCTTTTAGCTCCCTGAATGTTTGGATGGAAATGTGCTGATCTTGTTGGGGATACCAAGTCGAAGAATCGTTGATTCTGGCATTTGAATTTGCCTTCGAATTGAATAAGCACATGGAGATGAGGTTCCCCATCTTCGTGAAATTCTCTGGTTACACGAATAAATTTCTTGTTTGTTGGTGTTTTAAGTGCCTGTAATTGGGAAAGTGTTTCCTCTTTGGTTAATGAACAATGAGGGTATGTAAGGAAGAAATTTTTTGCATTTACAGTGAAACGCTTTGGCGGTGGCATTGTAGTAAATAAGAAGATGAGACCCGATAGCTCTCCTTCAAACTTGGCGAAATGAATTGGGGAATGGGTCTCAATATATAGTAGAGTCCATTATAGAATATAATTGCCACGTGGCGGCCATCCGATATAATATT |
| 1 | >Tomato golden vein virus TGVV:BR:G1:To:2003-2005 segment DNA-B, complete sequence  ACCGGATGGCCGCGCGATTTTTTTTTGGTGGACCCATTCTGAAAGTAATTGAGCGCATTTTTGACGTCCGCGAATTGAGTTGAGCGCAATATTTGAGTTCCGGAAATATAGTTAGTGGAGTAACTTTAATTTGAAATAAATTACAGCTTTTACGGTAACCAATCAGCTTGCGATTCAGGAGTCTATTTATCTATTACATTTATGTCAGTGTCAAGGTATACGGTATTCTTCACGTGGACCAATTAATTGTTCGCTATGGAGTCTAACTAATTAATATATGCAGTGATGATATATATAAATTCCATATATATTGTGATAGATATGATATCGCATCATGTCTCCTACTAGCTATAGACGTGGTTGGTCGTTTGCTCAACGACGAGGTTATTCTCGTAAATATTTGTTCAAACGTCCTTATTATTTTAAACGTACAGATGGGAAACACCGATCGGGTAACTCTAATCAGGTCAATGAGGATATTAAGTTGTCGCAACAGCGTATACATGAGAACCAGTTTGGTCCGGAATTTGTAATGGGTCATAATACAGCAATATCGACTTTTATTACTTTTCCTAGTCTATGTAAAACTGAACCGGGCAGATCCAGGGCATATATTAAGTTGAAACGTTTACGTTTCAAAGGTACCGTCAAGATTGAGCGTGTTCATGCTGATGTGACTATGGATGGTATAATACCAAAGATTGAAGGCGTATTTTCTATGGTTGTCGTTGTTGATCGTAAACCCCATTTGAGTTCATCTGGTTGTCTGCATACATTTGATGAGCTCTTTGGTGCACGGATCCACAGTCATGGCAATTTGGCTGTAACTCCATCTTTGAAAGAGCGTTTTTACATACGTCACGTTTGGAAGAAAGTAATATCTGTTGAGAAGGACAGTATGATGGTTGATCTTGAAGGAACGACATCATTGAGTAATAAGCGTTTTAATTGTTGGTCTGCTTTTAAAGATCTTGATCGTGATTCATGTAATGGTGTTTATGCGAATATAAGCAAGAACGCCCTTTTAGTTTATTACTGTTGGATGTCGGATACGATGTCTACGGCGTCATCATTTGTATCGTTCGATCTTGATTATGTTGGTTAAAAATTAAAATATTAATGTATTTATACTTACATGAGAAAATAATTCATTTAAGAAAACGTGAGAAAATAAATCATTTAAGAAAACGTGAGAAAATAAATCATTTAAGAAAACGTAATCACATAATATAATTATAATTACTGAAATATTGGCTGCGCAGCTGCATGCAAAAAAAAATATTGGCAACTTGATATTTACTTCAATGATTTGGGTTGTGGAGGAATACAGTTAGATTTAATACATTCTTGGACTGTTGACCTAACAAGATCGTTTAATTGGGCCATTGACAGCGTGATGTTGGATTGCGTTCTTTGGGCCCCAATTATTGAAGCCGAATCACCTGGATCTAATATGGCAGTTCCTAGCCTATTTAGATCTTTGTATGGATGTAACGCCTCCCCTAGGTCTGATTGGGCTTCTAATTGGGTCGTCTCTATTGTACTCCTTGTGGCCCATGACTCTCCTGGATTTAGTTCTATTGGGCCATGAAGCCCAAATCTAGAAGTTGATGCCGATCTTATCAATTTTCTCTCCCACTTTCCATAACCTACATGAGAGAAGTCGATGTCATTGACGGTAAATTGTTTGGACATTATCTTCACTGTGGGTGCCCTGAAAGGTATATCAACCGAATGTTTTGCCGTTGATAGCTTCAATTTTCCTTTGAATTTTGCGAAGTGGGTCATCTGATGAACGTTTGAGTCAGAAACCTTATAATATAGCTTCCATGGAATTGGGTCTTTGAGTGAGAAAAATGATGACGAGAAATAGTGGAGGTCTATGTTGCACCTGATTGGAAAAGTCCATGACGCCTGTAAGGATTCATTTTCAGTCATCCTCTTGTCATGAATCTCCACAATTACTGAACCAGTTGCGTTGATTGGTACCTGTTGTCTATATTCAATGACGCAATGATCAATCTTCATACAGCTTCGATTTAGTCTTGCACTTAATTGAGTTGCTTTTGATGGAAATTGCAATATGATTTCGGTAAGGTCATGAGATAGTTGATATTCATCACGACGAGATTCTATATAATTAAAGGCGTTGGGTGGGCAAACTACTTGAGAGTCCATATGTATTACAAGATTGAATAAGTGTAGAGAATAATTTAGAAAAAGAAAAGGAAACAGTAGTAGTGATAGATAAGATGAGTGAATGGATGTGATATTATACAGAATTGTCGATCCTTATATAGACTCTTAACTGGTTATGGATAATTTGGTTAAACAATACTGAGTATACTTACTAAATAAGTTTATAAAGTTATGGTGAAAAGCTTTGATGGTGGTATTTATGTAAATAAGGAGGTGAGACCCGATAGCTCTCCTTCAAACTTGGCGAAATGAATTGGGGAATGGGTCTCAATATATAGTAGAGTCCATTATAGAATATAATTGCCACGTGGCGGCCATCCGATATAATATT |
| 1 | > Tomato mottle leaf curl virus ToMoLCV:BR:G1:To:2003-2005 complete sequence  ACCGGATGGCCGCGCGGGTTTTTTTGACCCGCTCCGTGACATATTTTTTGTCTTTTACTATGTGGTACAGTCAATAAATGACAAATATGACCGTCCAATCAGAAATGGTCCTCAAAGCCTAATTATTAAAAAATACTTGGTCACTAAGTTTGGTAAAGTTTATAAATGGTCCTTCCTCGTAATGTTATACCAACTTTAAGTCATAATGCCTAAGCGTGATGCCCCATGGCGCTCAATGGCGGGGACCTCAAAGGTTAGTCGGTCCGCCAATTTCTCCCCTCGTGGAGGAATCGGGTCGAAATCAGAGAAGGCCCAGGCCTGGGTTAACAGGCCCATGTTCAGGAAGCCCAGGATATATCGGATGTATAGAACCCCCGATGTACCAAAGGGATGCGAAGGCCCATGTAAGGTCCAATCCTTTGAGCAGCGTCACGATGTCCTTCATACGGGGAAGGTGATGTGTATTTCCGATGTCACTCGTGGTAACGGTATTACCCACCGCGTGGGTAAACGTTTTTGTGTCAAGTCTGTGTATATATTAGGCAAGGTCTGGATGGATGACAACATCAAGTTGAAGAACCACACGAATAGTGTGATGTTCTGGTTAGTTAGGGACAGGAGACCGTATGGCACTCCCATGGATTTTGGCCAGGTGTTCAACATGTTTGACAACGAGCCCAGTACCGCCACGGTGAAGAACGATCTCCGCGATCGTTACCAGGTCCTGCACCGGTTTTATGCCAAGGTTACAGGTGGACAATACGCCAGCAACGAGCAGGCTATTGTCAAGAGGTTCTGGAAGGTCAACAACCATGTGGTGTACAACCATCAGGAGGCTGGGAAATACGAGAACCATACGGAGAACGCTCTCTTATTGTATATGGCATGTACTCATGCCTCTAACCCTGTGTATGCTACTTTAAAAATTCGGATCTATTTTTATGATTCGATAACAAATTAATAAAGTTTAAATTTTATTGAATGATCTTCGAGTACATAATTTACATATGGTTTGTCTGTTGCGAATTGAACAGCTCTAATTACATTATTAATAGAAATGACTCCTAAACGATCTAAATACATATTAACTAAATCCCTAAACCTATTTAAATAAGTCGTCCCAGAAGCTGTCGTTGACGTCGTCCATATTTGGAAGTTGAGGAAGCACTTGTGGAGATCCAACGCTCTCCTCAGGTTGTGGTTGAACCGTATCTGTATGTGGTATATCCTTGTGTTCGTGTGTATCGGGTTCTCTACGTTTGTTATCTTGAAATATAGGGGATTTGGGACCTCCCAGATAAACGCGCCATTCTGTGCCTGATGTGCAGTGATGAGTTCCTCGGTGCGTGAATCCATTGCCGATGCAGTCGATGTGTTGATATATTGTGCAGCCACAGTTTAGGTCTATGCGCTTGCGTCGAATGACCCTCTTCTTAGCTATCCTGTGTTGTGGTTTTATAGAGGGGGGTGTCGAGGAAGATGAATTTTGCATTGTGGAGTGTCCAGTTTTTGAGGGCTGTGTTTTCCTCTTTGTCGAGAAAATCTTTATAGCTGGCCCCTTCTCCTGGATTGCATAGCACGATTGAGGGTATCCCTCCTTTAATTTGAACTGGCTTTCCGTATTTGCAGTTTGATTGCCAGTCCTTTTGGGCCCCAATAAGCTCTTTCCAATGCTTCATCTTTAGATATTGCGGAGTGATGTCATCAATGACATTGTACTCCGCTTCGTTTGAGTAAACCCTATGATTGAAATCCAGGTGTCCACTCAAATAGTTATGTGGGCCTAATGCACGCGCCCACATTGTCTTCCCCGTCCTCGAATCACCCTCGATAATCAAACTAATAGGTCTCTCCGGCCGCGCAGCGGGATCCAGACCAAAATAATCATCAGCCCACCATTGCATCTCCTCCGGAACCTCAGTAAAAGAGGAGAGTGGAAACGGAGGAACCCATGGTTCCGGAGCCTTTTTAAAGATGCGTTCTAAATTAGTATGGATGTTGTGATGTTGAAGGACGAAGTCCTTCGGTTGCTCCTCCCTCAATATATTGAGGGCCTCCATGACGGATCCTGCGTTGAGGATCTTGGCATACGTGTCGTTGGCAGATTGCTGACCTCCTCTAGCTGATCTGCCGTCGATTTGGAAAACTCCATGATCAATGAAGTCTCCGTCTTTCTCCACGTAGGATTTGACATCTGATGAGCTCTTAGCTCCCTGAACGTTCGGATGGAAATGTGTTGATCTGGTTGGGGATATGAGGTCGAACAATCGTTCGTTTGTGCACTGTAATTTCCCTTCGAACTGGATGAGCACGTGGAGATGAGGTTCCCCATCTTCGTGAAATTCTCTGGCAACTCGGATGAACAATTTATTAACTGGCGTCTGTAACCCTAATAATTGAGATAGAGCTTCTTCTTTTGTTAAAGAGCACTTTGGATAAGTGAGGAAATAATTTTTAGAGTTGATTCTAAAACGACGTGGTAATGGCATTTTTGTAATTAAGGCGTGTACACCAATTGAGTTCTCTCTAAAACTCTATGGCAATTGGTGTATTGGGGTACAATATATACTAGAACCCTCAATAGAACTTTCAATCTCGTTCACACACGTGGCGGCCATCCGATATAATATT |
| 1 | >Tomato rugose mosaic virus ToRMV:BR:G1:To:2003-2005 segment DNA-A, complete sequence  ACCGGATGGCCGCGCGATTTTTTTAAGGCCCATTTATGTTGGGCCTCTCTTTTGGGCCTTCACCTTTAATTTTATTGAAAGTAAATGTGGCCCAATCATGATGCATTTGACGAGCTAAGATATTTACAACTACTTAACGCCCAAGTTGCATAACGGCTATAAATTAAACGTGCATATGTCGATAACTTTAATTCAAAATGCCTAAGCGCGATGCCCCATGGCGCCACATGGCTAGTACATCTAAGATTAGCCGGGGCGTTAATAATTCTCCTCGAGCAGGAGTTGGGCTACGGTCCAACAAGGCCTCTGACTGGGTAAACAGGCCCATGTACAGGAAGCCCAAGATATATCGGATGTATAGAACCCCCGATGTTCCAAGGGGTTGTGAAGGCCCATGTAAAGTGCAATCGTTTGAACAGCGTCACGACATTTCCCATACTGGTAAGGTGATGTGTGTTTCCGATGTGACACGTGGTAACGGTATTACACACCGTGTGGGTAAGCGTTTCTGTGTTAAGTCTGTGTATATTCTGGGTAAGATATGGATGGATGAGAACATCAAGTTGAAGAACCACACGAACAGTGCCATGTTCTGGTTGGTCAGGGACCGTAGACCGTATGGCACCCCTATGGACTTTGGCCAGGTGTTTAACATGTTCGATAACGAGCCTAGCACCGCCACTGTGAAAAACGATCTCCGTGATCGTTTTCAAGTTATGCACAAGTTCTATGCTAAGGTCACAGGTGGACAATACGCAAGTAACGAGCAGGCGCTTGTCAAGCGTTTCTGGAAGGTCAACAACCATGTAGTCTACAACCATCAAGAGGCAGGGAAATACGAGAATCATACTGAGAACGCCTTGCTATTGTATATGGCATGTACTCATGCCTCTAACCCCGTGTATGCTACATTGAAAATTCGGATCTATTTTTATGATTCGATTACTAATTAATAAAATTTAAATTTTATTGAATGATTTTCGAGTACAGCATTTACATATGATTTGTCTGTTGCGAAACGAACAGCTCTGATTACATTATTAATGGAAATAACGCCTAACTGATCAATGTACAACATAAGTAAATATTTAAATCTATTTAAATATGTCATCCCAGAAGCTCTCAGGGATGTCGTCCAGATTTGGAAGTTGAGATAAGCCTTGTGGAGACCCAGTACTCTCCTCAGGTTGTGGTTGAACCGTATTTGTACGTGGTAGACTCTGGTCCTCGTGTATAATATGTCCTCGACGTTGTACATCTTGAAATAAAGGGGATTTGATATCTCCCAAATATAAACGCCATTCTCTGCCTGACGTGCAGTGATGAGTTCCCCTGTGCGTGAATCCATGTCCTCTGCAGTCTATGTGTACGAAAATGGAGCAGCCGCACTGTAAATCAATTCGTCTTCTTCGTATAGCTCTCCTCTTAGCAGCCCGATGTTGAACTTTGATAGAGGGGGGAGTTGAGGAAGATGAATTGTGCATTGTGCTTTGTCCAACTATTTAATGCTGAGTTTTCTTCTTTGTCGAGGAAACATTTATAGCTGGCCCCCTCGCCAGGATTGCAAAGCACGATGCATGGGATACCACCTTTAATTTGAACTGGCTTTCCGTATTTGCAGTTTGATTGCCAATCCTTTTGGGCCCCAAGCAATTCTTTCCAGTGCTTTAACTTTAGATAGTGCGGTGCGATGTCATCAATGACGTTATATTCAACATGATTTGAATAAACCCTAGGATTGAAATCTAGGTGTCCACTCAAATAATTATGGGCCCCTAATGCACGTGCCCACATCGTCTTTCCCGTTCGAGAATCACCTTCAATGATGATACTAATAGGTCGTTCCGGCCGCGCAGCGGCACCTCTCCCAAAATAGTCATCTGCCCAAGCTTGCATCTCTCTCGGTACGCTAGTGAAAGAGGAGAGGGGAAACGTAGGGGCCCATGGCTCCGGAGCCCTTGTAAATATCCTATCTAAATTACTATTTAGATTGTGAAACTGAAATAAGAACTTTTCAGGCAGCTTCTCACGGATTATCTGCAGGGCGACGTCTTTGGAAGGTGCGTTCAAGGCTTCTGCGGCAGCGTCGTTAGTTGTCTGGCAACCGCCTCTAGCACTTCTTCCGTCGATTTGGAATTCCCCCCACTCGATAGTATCTCCGTCCTTGTCGACATAGGATTTGACGTCGGACGATGACTTAGCTCTCTGAATGTTCGGATGGAAATGTGTTGACCTTGTTGGGGAGACCAGGTCGAAGAATCGTTGATTTTGGCAGCAGTAGTTTCCCTCAAATTGAAGAAGCACGTGGAGATGAGGCTCCCCATTTTCATGGAGCTCTCTGCAAACCTTGATGAACTTCTTATTTGTAGGAGTGTTTAGGGTTTTTAATTGGGAAAGTGCTTCTTCTTTCGATAATGAGCATTTGGGATATGTGAGGAAATAATTCTTCGCTTTTATTTGAAAGCGCTTTGGAGCTGATGGCATATTTGTAAATATGACCCTTACTACCAAATGGTAGCTGCTCTAAAACTCATATGAATTGGTAGTTATGGTAGCTCTTATATAGTAGAAGTTCTTTTTAAGGAGATTGCTACACGTGGCGGCCATCCGTTATAATATT |
| 1 | >Tomato severe rugose virus ToSRV:BR:G1:To:2003-2005 segment DNA-A, complete sequence  ACCGGATGGCCGCGCGATTTTTCACCCCTTTAGTTTCAATTAAAGTAAAGTGATTGTCTGTGGCCCAATCATATTGGGCCTGTCGAGCTTAGATATTTGTAACAACTTAAGGCCCAAGTTGTTAAACGGCTATAAATTGAACATACACTTTACTTTTGCTTTAATTCAAAATGCCTAAGCGTGATGCCCCATGGCGTTTAACGGCGGGAACTTCAAAGGTTTCCCGCTCTGTCAATTATTCTCCCCGTGCAGGATATGGACCCAAATATAACAAGGCCGCTGAGTGGGTGAACAGGCCCATGTACAGGAAGCCCAGGATCTACCGTACTTTGAGAGGCCCAGATGTTCCTAGAGGCTGTGAAGGGCCTTGTAAGGTTCAGTCTTACGAGTCTCGTCATGATGTTTCCCATGTCGGGAAGGTGATTTGTGTGTCTGACGTTACACGTGGTAACGGTATTACTCACCGTGTTGGTAAGCGTTTCTGCGTGAAGTCTGTATATATTTTAGGGAAGGTATGGATGGACGAGAGCATCAAGTTGAAGAATCACACAAATAGTGTGATGTTCTGGTTGGTTAGAGATCGGAGACCTTATTCGTCACCTATGGATTTTGGCCAGGTGTTCAACATGTTTGACAACGAGCCTAGCACTGCAACTGTTAAGAACGATCTTCGGGATCGTTTTCAGGTCATGCACAAGTTTTATGCCAAGGTTACTGGTGGACAGTATGCCAGTAATGAGCAGGCATTAGTGAAGCGCTTTTGGAAGGTCAACAACAACGTAGTCTATAACCATCAGGAGGCAGGGAAATACGAGAATCATACTGAGAACGCCTTGCTATTGTATATGGCATGTACTCATGCCTCTAACCCCGTGTATGCTACATTGAAAATTCGGATCTATTTTTATGATTCGATTACTAATTAATAAAATTTAAATTTTATTGAATGATTTTCGAGTACAGCATTTACATATGATTTGTCTGTTGCGAAACGAACAGCTCTGATTACATTATTAATGGAAATAACGCCTAATCGATCTAGATACAATAAGACTAAATATTTAAATCTAGTTAAATATGTCGTCCCAGAAGCTGTCAGTGAAGTCGTCCATATCTGGAAGTTGAGGAAGCTCTTGTGGAGATGCAATGCTCTCCGCAGGTTGTGGTTGAACCGTATTTGGACGTGGTAGACTCTGCTCGCGGTGTACAGTGGATCCTCCACTCTGTTTATCTTGAAATAGAGGGGATTTGATATCTCCCAAATATAAACGCCATTCTCTGCCTGACGTGCAGTGATGAGTTCCCCTGTGCGTGAATCCATGTCCTCTGCAGTCGATGTGTACGTAAATAGAGCACCCGCACTCTATATCAATTCGTCGTCTCCTGATTCCTCGTTTTTTAGCAGCTCTGTGTTGTACCTTGATAGAGGGGGGTGTTAAGGAAGACGAATTTCGCATTGTGCTTTGTCCAATTATTTAGACTTGCATTTTCTTCTTTGTCGAGGAAACATTTATAGCTGGCCCCCTCGCCAGGATTGCAAAGCACGATGCATGGGATACCACCTTTAATTTGAACTGGCTTTCCGTATTTGCAGTTTGATTGCCAATCCTTTTGGGCCCCAAGCAATTCTTTCCAGTGCTTTAACTTTAGATAGTGCGGTGCGATGTCATCAATGACGTTATATTCAACATGATTTGAATAAACCCTAGGATTGAAATCTAGGTGTCCACTCAAATAATTATGGGCCCCTAATGCACGTGCCCACATCGTCTTTCCCGTTCGAGAATCACCTTCAATGATGATACTAATAGGTCGTTCCGGCCGCGCAGCGGCACCTCTCCCAAAATAGTCATCTGCCCAAGCTTGCATCTCTCTCGGTACGCTAGTGAAAGAGGAGAGGGGAAACGTAGGGGCCCATGGCTCCGGAGCCCTTGTAAATATCCTATCTAAATTACTATTTAGATTGTGAAACTGAAATAAGAACTTTTCAGGCAGCTTCTCACGGATTATCTGCAGGGCGACGTCTTTGGAAGGTGCGTTCAAGGCTTCTGCGGCAGCGTCGTTAGTTGTCTGGCAACCGCCTCTAGCACTTCTTCCGTCGATTTGGAATTCCCCCCACTCGATAGTATCTCCGTCCTTGTCGACATAGGATTTGACGTCGGACGATGACTTAGCTCTCTGAATGTTCGGATGGAAATGTGTTGACCTTGTTGGGGAGACCAGGTCGAAGAATCGTTGATTTTGGCAGCAGTAGTTTCCCTCAAATTGAAGAAGCACGTGGAGATGAGGCTCCCCATTTTCATGGAGCTCTCTGCAAACCTTGATGAACTTCTTATTTGTAGGAGTGTTTAGGGTTTTTAATTGGGAAAGTGCTTCTTCTTTCGATAATGAGCATTTGGGATATGTGAGGAAATAATTCTTCGCTTTTATTTGAAAGCGCTTTGGAGCTGATGGCATATTTGTAAATATGACCCTTACTACCAAATGGTAGCTGCTCTAAAACTCATATGAATTGGTAGTTATGGTAGCTCTTATATAGTAGAAGTTCTTTTAAGGAGATTGCTACACGTGGCGGCCATCCGTTATAATATT |
| 1 | >Tomato severe rugose virus ToSRV:BR:G1:To:2003-2005 segment DNA-B, complete sequence  ACCGGATGGCCGCGGGATTTTTCTTATCTGCTACGTGGCGAAATCGTGTACGTTGCCTCGCGCTTTCCATTTTAATTGAGCGCCTTTTTGAAGTCCGCGAAATGAGTTAATTGTCTTTTTGAAATCCGCTGTTTGTGCATCACCTTTAATTTGAATTAAAGGTTGGATAGTTCATATTGATCAATCATTTCGCTTGTTTATTTCCTGTCGTGGTGTAATTACAACCGTTCGTTAAAAATATAAGAAATTTACGACGTGGACTGTCTAAATTTCATCTACATAGTTAATTTGACAAATGAATGCATATTTAAACTCTGCTTTCGTGTGGGTTTACACCACGTCTATACATATTGTCCAGGTTATTTTGTATAAGTATAATTTTTATTTTGTCTTATCTTATTATCTGAACATGTATCCCATTAAGTATAGACGTGGAATGTTGTTTAATCATCGACGAGGTTACTCATCTAATCCCGTATTTAAGCGTTTACACGGAGCGAAACGAAGTGATTTCAAGCGTCGTTCGAGTAATCAGATTAAGAGTATGGATGAGACTAAAATGTCTGTTCAGCGGATTCATGAGAACCAGTTTGGCCCTGAATTTGTAATTGGCCACAATTCTGCCATATCCACATTCATTACATTCCCTACTCTTTGTAAGACTGTCCCTAACCGTTGTAGGTCATACATAAAGTTAAGACGTCTACGTTTTAAAGGAACAATCAAGATTGACCGTGTTCATGCTGAGGTGAATATGGACGGTACAAGTCCAATGATTGAAGGAGTCTTCTCTCTGGTTGTGGTCGTTGATCGCAAACCTCATTTGGGCTCATCTGGAACTCTGCATTCTTTTGATGAGATATTTGGTGCAAGGATTCATAGCCATGGTAACCTGGCAATAGTATCCTCTCTGAAAGAGCGTTTTTACATACGTCACGTTTGGAAGAAAGTAATATCTGTTGAGAAGGATACAACCATGGTTGATGTTGAAGGAAGTACTACTTTATCTAACAGGCGTTTTAATTGTTGGTCATCCTTTAAGGATATTGACCGTGAATCATGTAATGGTGTTTATGCAAACATAAGCAAGAACGCCCTGTTAGTTTATTACTGTTGGATGTCTGATAATGTGTCTAAGGCATCGACATTTGTATCATTTGACCTTGATTATGTTGGCTAAATAGTAAGTTTAATACGTTATTAGTAATAATGTTTAACTCGCTGTAATAATAAATACAAGTACTTGTTCAGTAATAATGTTCAGCTCGCAGTAATAACAAAAAAAATGTGTTTAACAATATAGAATTTATTTTAAAGATTTTGGCTGTGAAGGAGTACAGTTGCTGTTTATACATTCATGGGCCGCTGCTTTAACAAGTTCGTTTAATTGGGCCATTGACAGTGTTATGTTCGATTGGGCCCTTTGAGCACCAATTACGGACGCAGAATCACCTGGGTCTAATGTGCCTGTACCCAATCTATGTAATTGTCTGTATGGATGCATCGCGTTCTCTATATCTGAGCCCGCCTCTGATGGGCTTATGCCTACAGTACTTCTAACAGCCCATGACTCTCCGGGCATTATTTCAAGTGGGTTGTGAAGGCCCAATCTTGATATTGAGGCGGATCTGATCATCTTCCTTTCCCATTTGCCATAGCCTACGTGACTGAAATCAATGTCCTTATCTGTAAATTGTTTTGAAAGGATCTTCACCGTTGGGGCACGGAAAGGTATATCAACCGAATGTTTAGCCGTCGAGAGTTTCAGTCTCCCTTTGAATTTAGCGAAGTGGGTACTCTGGTGAACGTTCGTGTCACATACTCTGTAGTACAATTTCCATGGAATTGGGTCCTTAAGGGAGAAGAACGACGACGAAAAGTAGTGAAGATCTATGTTACATCTGATAGGAAAGGTCCAAGACGCCTGTAGTGATTCATTGTCCGTCATTCTTCTGTCATGAATTTCGACTATAACTGAACCTACGGCGTTAATTGGTACCTGTTGTCTGTATTCTATTACGCAATGGTCGATTTTCATACAGCTACGACTAAGCCTTGCGCTTAATTGAGACGCCGTTGAAGGGAATTGAAGCATAATCTCGGTTAGATCATGGGAAAGCTGATATTCATCACGCTTAGATTCTATATAATTAAATGCGTTGGGAGGATTTACAAGCTGAGATTCCATTATTGAAAATAGGGAGCGCAGCGACAATGTTTGAGGAAATTTAATAAGGGAAGATGATAATATTTTCGTCAACTGAATATATGACAAGAAAATTGTCTGTTGATCCAATTGGGAAATTGGAGATGATAATAACTAAAAAACGAGGAATCAGGAGACGACGAATTGAGAGGAGATATTGTCTATTTTAGATGTAATTGATAAATACCCCTCTATTTGCTCTTTAAATAGAATTTTTAAGGCAATGGCATATTTGTAAATATGACCCTTACTACCAAATGGTAGCTGCTCTAAAACTCATATGAATTGGTAGTTATGGTAGCTCTTATATAGTAGAAGTTCTCTTTAAGGAGATTGCTACACGTGGCGGCCATCCGTTATAATATT |
| 1 | >Tomato apical leaf curl virus ToALCV:BR:G1:To:2003-2005 complete sequence  ACGGCTTTGCCCCTCACTTTCTTTCTTATCTGATCTGCCACGTGGTTGATCCGCCTTAATTTACGGAAAAGTTGTCGGGTATAGTATAAAGCAGCCGTAACTTTTGACTAAGTTCAAAAGTATTGAGCCCACATGTCTTGGCGGGAAACTTTTCCGTAACTTCGATTACTTGCGGAGCAAGTAATCTTATTATAAAAGAATAAATAAATGAATAAAAAATTCAATGGACTGGACCTTCAACGCTTGGGTCTATCTATTTGTTTTTATAAGTACAGTTATCATTACTGTATTTAATGGAGTTACCAGTATACAGCTCCTCAGAATATCCGAGAAGTTACAACGCATTTCTGCAGACTTGCGCGGATCGATTAATCACCTGGCAATTGCAATTGGTGTGGCAGAAGGTCGAGTATTACAACCTCCACAAGGCGGACGAGTTCTTCGAGTGGGAAGAACTTTATCAGTTCCTAATAGAGTACAGGAAGACCGTCCGACGGGCGGGGAGACGCAAGTCTCACCATAAAAAAATAGAAATCTATAATCTCTTTTGGATTAAAAGAGATGGTAGGAAATCAGGCCTTAATAAGTTGGGGTGCGAAGAAAAAGCGAAGTACCCCAAACCGTTCGATTTGGACTCGGAGAGTTCGTAGGCGTCTTGCCTATTACCCTAGGTATATACCTAGGGGTCCCAAAAAAAATACCCGTGATAAGGTTTATACATTTACCAGTTATAAAGCCTTTAGCGATTTAGGTGGTGTATGGTTGTTAAATGGTTTTGCCCAAGGATTGGGTAATAACCAAAGAAATGGAAGCGTTGAAATTATTCGCGCTTTAAATTTGCGTTTAGGTGTTGCTATAGCAGAAGGTGCCACAGCATTAAATTTTGCCAGGGACTGGAACTGTACCTGGTATGTAATTGCAGACCAGAGTCCTGGCACAACTATTCCAGCTTTTTCAGACATTTTTACCACAGGTTCTGCTGCTGTTGATGGTCTGGAATATATTCCAGATGATATGCATCTTCGTTTTCAGATTAAGATGAAGGGTAAATTGAAGTTTATTACTTCTGGAGTTAGTTATTCTACAGCAGTTACTTCCAAAACTGGCTATAATAAGTGCCATGATATAGTTAGAAAATTTAAATCAAAGTTGAATGTAAGAACTGATTATGATAGTAACAGTTCTGGAGGTGCTATTACAGATATAAAAAAGGGTGCTTTGTATTTAGTTATTAATCCCGAATTCAGCTGTTACGCTGGTGTAAATTGCACTATGTATCATAGTGGTCCTTATTATAAATAAAATATTTTAGTTTGTTCTTACGTATATCACCCGTCACCCTCACCTTCCCCCCCAAGAATGTGTTCATACGGTTGAGCCCAGAGCCCTCACCTTCCCCCCCCAAATAAGCAGGCGTTCTTTGATTTGGTAGGGCTTTAGCCCTACCAAATCAAAAGAACAAGATGGAATGAAGAAATAGTGGTTTATCTTGGCAGCAATTCCTTAAAAGGGTTAAGCAATAGCAGAGTTACATTCATACGGAACAGGGTATAATATGATAAAATAACTCTAAAAAATTGATCCATCAATGAACTCATAAAGGACATTAGACTGACACCAAACATACAAATCAGAACGACTGATAGCCTCTTTATAAGAACTATCAGGATTACATAATATTATCGAAGGAATGCCTCCTTTTATTGTACATTTTTTCCTATACTTTTCATTAACCGTGAAATCTTTTTGTGCTCCAAGTATTTCCTTTTTGCACGGCAAGTATTGAAACGGTATGTCGTCGAATACATTATATATAGCGTGTGGATTATACTCAGTCCAGTTGACTCCTCCGCAGAAATAATTGTGTAGTCCTAAGCTTCTTGCCCATGCTGTTTTACCTGTTCTCGTCGGACCTTCAACAATCAGCGTTAATGGCCTATCTGGTTTATTCTCCGGGTCCTGAATCATTATAAATGTGTGGTCTAATGTTAGTGAAATTATCTGGGTTTTCAAGAATCTGTTCTATAGATTCTTGTGCCATATTATCATAAAAATCTAAATCATTTTGTGTTAATTGCGGAATGTCTAACAAAATGAAATCTCTATGTACCACACATATGTTCTGTTCTACCCAATTTTTAATGGGCTCGGGTACGCCATTGAATATTGAGAATTTGGGTGTAAAATTCGCACTTGGTTTTGGCCACTTTTTGTTGGCCATGTATTCGAGGTTTCGCAGTTGTGTTGCGTATGTATAAGGTTGTTCTCGTTGACATCTGCCGAGGAATTCCTCGGCGTTAGTGGATTCGTCGATGATCTTCCCCCATATAGTATCCCGAGATTTCTTAGGACTGCGTCTTGAAGCTCTAAGTAATCCTCGTTCCTCGAATACTCCTCCCTTAGCGATGTAGTCTGCAACGTCAGCGTCCCTTCTTGGAATCTGGGCGTTTGGATGGTATGTGCTCTCTCTATTAGGGTCTGTAATGTCGAAGAATCTTGGGTTTCTTGTACTGAATTTCTCGTCCAATTGGACGAGGCAGTGGAGATGCAGCTCCCCATCTTGATGAGTCTCTTGACAGACCCTTGCATATGTAATTTTAAAATCATTTAAAAGGTGAAATAAATAGTCTATGACGAATAATGGAATGAGAGGGCATTTTGGGTATGTAAGGAAAATTGATTTTCCCTGTAGGCGAAATGTGTTTGGTGTTCTTGCCATTTTTCTCTTTATTATATTCTATTACTATGGCTTTGCGGAGCCAGAGCCAATTTATAGTCGAAGGTGTTAAATGGGCTTTGTGGCTCTATTCAGGTCAGCCGTTGATTATGGTATTACGTGGCAGAAATCCGTGAAGGGGCAAAGCCATATAATATT |
| 1 | >Tomato begomovirus-1 Bego1:BR:G1:To:2003-2005 complete sequence  ACCGGATGGCCGCGCGGTTTTTTTTGACCCGCTCCGTAAATCATTTCTTTGTAATTTACTATGTGGTACAGTCAATAAATGACAAATATGACCGTCCAATCAGAAATGGTCCTCAAAGCCTAATTATTAAAAAATACTTGGTCACTAAGTTTGGTAAAGTTTATAAATGGTCCTTCCTCGTAATGTTATACCAACTTTAAGTCATAATGCCTAAGCGTGATGCCCCATGGCGCTCAATGGCGGGGACCTCAAAGGTTAGTCGGTCCGCCAATTTCTCCCCTCGTGGAGGAATCGGGTCGAAATCAGAGAAGGCCCAGGCCTGGGTAAACAGGCCCATGTACAGGAAGCCCAAGATATATCGGATGTATAGAACCCCCGATGTTCCAAGGGGTTGTGAAGGCCCATGTAAAGTCCAATCGTTTGAGCAGCGTCACGATGTCCTTCATACGGGGAAGGTGATGTGTATTTCCGATGTCACTCGTGGTAACGGTATTACCCACCGCGTGGGTAAACGTTTTTGTGTCAAGTCTGTGTATATATTAGGCAAGGTCTGGATGGATGACAACATCAAGTTGAAGAACCACACGAATAGTGTGATGTTCTGGTTGGTTAGAGATCGGAGACCTTATTCGWCACCTATGGATTTTGGCCAGGTGTTCAACATGTTTGACAACGAGCCTAGCACTGCAACTGTTAAGAACGATCTTCGGGATCGTTTTCAGGTCATGCACAAGTTTTATGCCAAGGTTACTGGTGGACAGTATGCCAGTAATGAGCAGGCATTAGTGAAGCGCTTTTGGAAGGTCAACAACAACGTAGTCTATAACCATCAGGAGGCAGGGAAATACGAGAATCATACTGAGAACGCCTTGCTATTGTATATGGCATGTACTCATGCCTCTAACCCCGTGTATGCTACATTGAAAATTCGGATCTATTTTTATGATTCGATTACTAATTAATAAAATTTAAATTTTATTGAATGATTTTCGAGTACAGCATTTACATATGATTTGTCTGTTGCGAAACGAACAGCTCTGATTACATTATTAATGGAAATAACGCCTAATCGATCTAGATACAATAAGACTAAATATTTAAATCTAGTTAAATATGTCGTCCCAGAAGCTGTCAGTGAAGTCGTCCATATCTGGAAGTTGAGGAAGCTCTTGTGGAGATGCAATGCTCTCCGCAGGTTGTGGTTGAACCGTATTTGGACGTGGTATATCCTTGTGTTCGTGTGTATCGGGTTCTCTACGTTTGTTATCTTGAAATATAGGGGATTTGGGACCTCCCAGATAAACGCGCCATTCTGTGCCTGATGTGCAGTGATGAGTTCCTCGGTGCGTGAATCCATTGCCGATGCAGTCGATGTGTTGATATATTGTGCAGCCACAGTTTAGGTCTATGCGCTTGCGTCGAATGACCCTCTTCTTAGCTATCCTGTGTTGTGGCTTGATAGAGGGGGGTGTTAAGGAAGACGAATTTCGCATTGTGCTTTGTCCAATTATTTAGACTTGCATTTTCTTCTTTGTCGAGGAAACATTTATAGCTGGCCCCCTCGCCAGGATTGCAAAGCACGATGCATGGGATACCACCTTTAATTTGAACTGGCTTTCCGTATTTGCAGTTTGATTGCCAATCCTTTTGGGCCCCAAGCAATTCTTTCCAGTGCTTTAACTTTAGATAGTGCGGTGCGATGTCATCAATGACGTTATATTCAACATGATTTGAATAAACCCTAGGATTGAAATCTAGGTGTCCACTCAAATAATTATGGGCCCCTAATGCACGTGCCCACATCGTCTTTCCCGTTCTCGAATCACCCTCGATAATCAAACTAATAGGTCTCTCCGGCCGCGCAGCGGGATCCAGACCAAAATAATCATCAGCCCACCCTTGCATCTCCTCCGGAACCTCAGTAAAAGAGGAGAGTGGAAACGGAGGAACCCATGGTTCCGGAGCCTTTTTAAAGATGCGTTCTAAATTAGACCTAATGTTATGATGTTGAAGCACGAAATCTTTGGGTTGTTCTTCTTTTAATATATTGAGTGCTTCCATGACTGATCCTGCATTGAGAACCTTGGCGTACGTGTCGTTTGCAGATTGCTGACCTCCTCTAGCTGATCTGCCATCGATCTGGAAAACTCCATGATCAACGAAGTCTCCGTCTTTTTCCACGTATGATTTGACGTCTGACGAGCTCTTAGCTCCCTGTATGTTTGGATGGAAATGTGCTGACCTACTTGGGGAGACGAGGTCGAAGAACTTGTTGTTTTTGCAGTTGTATTTACCTTCGAATTGGATGAGCACGTGGAGATGAGGTTGCCCATCTTCGTGTAGTTCTCTCGAGACACGAATGAACAATTTATTAGTTGGTGTGTTTAGTGCTAATAATTGGGAAAGTGCTTCTTCTTTGGAAAGAGAACAGTGTGGATATGTTAGAAAATAATTTTTGGAATTTACTCGGAAATGTCTGGGCGGTGGCATTTTTGTAAATAAGGGTGTGTACACCAATTGAGTTCTTTCTAAAACTCTATGGCAATCGGTGTATTGGGGTACAATATATACCAGAAGCCTCTATAGAACTTTCAATCCTAGTCACACACGTGGCGGCTATCCGTATAATATT |
| 2 | >Sida micrantha mosaic virus SiMMV:BR:G2:To:2009-2011 segment DNA-A, complete sequence  ACCGGATGGCCGCGCGATTTTTCCCCCCCCCCCACGTGGCGCTCTGGTGGTCGTGCGATCTCTCTCCCCCCCTCTCGCGCGACGTGGAGCTCTGGTGTCCGCGCGTTCCCCCTCGCGCGTTCTGCCTTTAATTTAAATTAAAGGAAATAACTTTCATCAGGACCAATGAAATTGCGTCTTTATAGCCTAGATATCTGCGAAAGACTTGGGCCCTAAGTTGTTGGCCTGCTATATAATTAAGTCATGCATGACGTCAGGAATTAATTCAAAATGCCCAAGCGGGATCCCTCATGGCGCCAGATGGCGGGAACCTCCAAGGTTAGCCGCTCTTCTAATTTCTCACCTCGTGGAGGTGGAGGCCCAAAATACAACAAGGCCTCAGAATGGGTCAACAGGCCCATGTACAGGAAGCCCAGGATATACAGGACGCTCAGGACGCCCGATGTTCCAAGAGGCTGTGAAGGGCCTTGTAAAGTCCAGTCATACGAGCAACGCCATGATATCTCACATGTCGGGAAGGTCATGTGCATCTCTGACGTCACACGTGGCAACGGTATAACCCACCGTGTCGGTAAGCGTTTCTGCGTTAAGTCTGTRTACATTTTAGGGAAGATATGGATGGACGAGAACATCAAGCTCAAGAACCACACGAATAGTGTGATGTTCTGGTTAGTTAGGGACAGGAGACCGTATGGCACTCCCATGGATTTTGGCCAGGTGTTCAACATGTTTGACAACGAGCCCAGTACTGCCACGGTGAAGAACGATCTCCGCGATCGTTACCAGGTCATGCACAAGTTCTATGCCAAGGTTACAGGTGGACAATACGCCAGCAACGAGCAGGCTATTGTCAAGAGGTTCTGGAAGGTCAACAACCATGTGGTGTACAACCATCAGGAGGCTGGGAAGTATGAGAATCACACGGAGAATGCTTTGTTACTGTATATGGCATGTACTCATGCCTCTAACCCTGTGTATGCAACGCTTAAGATTCGAATCTACTTCTACGACGCGATAACCAATTAATAAAATTTGAATTTTATATCATGATCTTCAAGTACATAGTTTACATAGGCTTTGTCAGTGGCAAAGCGAACAGCTCTAATTACATTGTTAAGCGTGATTACGCCTAATTGGTCTAAGTACATCATGACTAGTCTCCTAAACCTAGTCAAATAAGTCGTTCCAGAAGCTGTCAGAGAAGTCGTCCAGACTTGGAAGTTCAGGTAAGCCTTGTGGAGACCCAATGCTCTCCTGAGGTTGTGGTTGAACCGTATTTGGACGTGGTAGACTCTGCTCGCGGTGTACAGTGGATCCTCCACTCTGTTTATCTTGAAATAGAGGGGATTTGATATCTCCCAAATATAGACGCCATTCTCTGCCTGACGTGCAGTGATGAGTTCCCCTGTGCGTGAATCCATGTCCTCTGCAGTCGATGTGTACGTAAATAGAGCACCCGCACTCTATATCAATTCGTCGTCTCCTGATTCCTCTCTTCTTGGCAATCCTGTGTCTCGGTTTGATAGAGGGGGGCGTCGAGGAAGATGAATTTAGCATTGTGGAGTGTCCAGCCTCTCAATGCCGCATTTTCCTCTTTGTCTAGGAAGTCTTTATAGCTGGCCCCCTCCCCAGGATTGCACAACACGATTGAAGGGACCCCACCTTCAATCAAACGAGGCTTGCCGTACTTGCAGTTTGTCTGCCATTTCTGTTGGGCCCCTATTAGTTCTTTCCAGTGTTTCAACTTTAGGTAATGCGGAGCGACGTCATCAATGACGTTATACTCCACTTGATCAGAATATACCTTTGAGTTGAAATCCAGATGGCCACTGAAGTAATTATGTGGGCCTAGAGCCCTAGCCCACATCGTCTTGCCCGTTCTGCAATCACCTTCAATGATGATACTAATAGGTCGTTCCGGCCGCGCAGCGGCACCTCTCCCAAAATAGTCATCTGCCCAAGCTTGCATCTCTCTCGGTACGCTAGTGAAAGAGGAGAGGGGAAACGTAGGGGCCCATGGCTCCGGAGCCCTTGTAAATATCCTATCTAAATTACTATTTAGATTGTGAAACTGAAATAAGAACTTTTCAGGCAGCTTCTCACGGATTATCTGCAGGGCGACGTCTTTGGAAGGTGCGTTCAAGGCTTCTGCGGCAGCGTCGTTAGCTGTCTGGCAACCGCCTCTAGCACTTCTTCCGTCGATTTGGAATTCCCCCCACTCGATAGTATCTCCGTCCTTGTCGACATAGGATTTGACGTCGGACGATGACTTAGCTCTCTGAATGTTCGGATGGAAATGTGTTGACCTTGTTGGGGAGACCAGGTCGAAGAATCGTTGATTTTGGCAGCAGTAGTTGCCCTCAAATTGAAGAAGCACGTGGAGATGAGGCTCCCCATTTTCATGGAGCTCTCTGCAAACCTTGATGAACTTCTTATTTGTAGGAGTGTTTAGGGTTTTTAATTGGGAAAGTGCTTCTTCTTTCGATAATGAGCATTTGGGATATGTGAGGAAATAATTCTTCGCTTTTATTTGAAAGCGCTTTGGAGCTGATGGCATATTTGTAAATATGACCCTTACTACCAAATGGTAGCTGCTCTAAAACTCATATGAATTGGTAGTTATGGTAGCTCTTATATACTAGAAGTTCCTTTAAGGAGATTAAGGGCCTAAAGCGGCCATCCGCACTAATATT |
| 2 | >Tomato mottle leaf curl virus ToMoLCV:BR:G2:To:2009-2011 complete sequence  ACCGGATGGCCGCGCGGGTTTTTTTTGACCCGCTCCGTGACGTATTTTTTGTCTTTTACTATGTGGTCCAGTCAATAAATGACAAATATGACCGTCCAATCAGAAATGGTCCTCAAAGCCTAATTATTTAAAAATACTTGGTCACTAAGTTTGGTAAAGTTTATAAATGGTCCTTCCTCGTAATGTTATACCAACTTTAAGTCATAATGCCTAAGCGTGATGCCCCATGGCGCTCAATGGCGGGGACCTCAAAGGTTAGTCGGTCCGCCAATTTCTCCCCTCGTGGAGGAATCGGGTCGAAATCAGAGAAGGCCCAGGCCTGGGTTAACAGGCCCATGTTCAGGAAGCCCAGGATATATCGGATGTATAGAACCCCCGATGTACCAAAGGGATGCGAAGGCCCATGTAAGGTCCAATCCTTCGAGCAGCGTCACGATGTCTCTCATACGGGGAAGGTGATGTGTATTTCCGATGTCACTCGTGGTAACGGTATTACCCACCGCGTGGGTAAACGTTTTTGTGTCAAGTCTGTGTATATATTAGGCAAGGTCTGGATGGACGACAACATCAAGTTGAAGAACCACACGAATAGTGTGATGTTCTGGTTAGTTAGGGACAGGAGACCGTATGGCACTCCCATGGATTTTGGCCAGGTGTTCAACATGTTTGACAACGAGCCCAGTACTGCCACGGTGAAGAACGATCTCCGCGATCGTTACCAGGTCCTGCACCGGTTCTATGCCAAGGTTACAGGTGGACAATACGCCAGCAATGAGCAGGCATTAGTGAAGCGCTTTTGGAAGGTCAACAACAACGTAGTCTACAACCATCAGGAGGCAGGGAAATACGAGAATCATACTGAGAACGCCTTGCTATTGTATATGGCATGTACTCATGCCTCTAACCCCGTGTATGCTACATTGAAAATTCGGATCTATTTTTATGATTCGATTACTAATTAATAAAATTTAAATTTTATTGAATGATTTTCGAGTACAGCATTTACATATGATTTGTCTGTTGCGAAACGAACAGCTCTGATTACATTATTAATAGAAATAACGCCTAAACGATCTAAATACATATTAACTAAATCCCTAAACCTATTTAAATAAGTCGTCCCAGAAGCTGTCGTTGACGTCGTCCATATTTGGAAGTTGAGGAAGCACTTGTGGAGATCCAACGCTCTCCTCAGGTTGTGGTTGAACCGTATCTGTATGTGGTATATCCTTGTGTTCGTGTGTATCGGGTTCTCTACGTTTGTTATCTTGAAATATAGGGGATTTGGGACCTCCCAGATAAACGCGCCATTCTGTGCCTGATGTGCAGTGATGAGTTCCTCGGTGCGTGAATCCATTGCCGATGCAGTCGATGTGTTGGTATATTGTGCAGCCACAGTTTAGGTCTATGCGCTTGCGTCGAATGACCCTCTTCTTAGCTATCCTGTGTTGTGGTTTTATAGAGGGGGGTGTCGAGGAAGATGAATTTTGCATTGTGGAGTGTCCAGTTTTTGAGGGCTGTGTTTTCCTCTTTGTCGAGAAAATCTTTATAGCTGGCCCCTTCTCCTGGATTGCATAGCACGATTGAGGGTATCCCTCCTTTAATTTGAACTGGCTTTCCGTATTTGCAGTTTGATTGCCAGTCCTTTTGGGCCCCAATGAGCTCTTTCCAATGCTTCATCTTTAGATATTGCGGAGTGATGTCATCAATGACATTGTACTCCGCTTCGTTTGAGTAAACCCTATGATTGAAATCCAGGTGTCCACTCAAATAGTTATGTGGGCCTAATGCACGCGCCCACATTGTCTTCCCCGTCCTCGAATCACCCTCGATGATCAAACTAATAGGTCTCTCCGGCCGCGCAGCGGGATCCAGACCAAAATAATCATCAGCCCACCCTTGCATCTCCTCCGGAACCTCAGTAAAGGAGGAGAGTGGAAACGGAGGAACCCATGGTTCCGGAGCCTTTTGAAAGATGCGTTCTAAATTAGAATGGATGTTGTGATGTTGAAGGACGAAGTCCTTCGGTTGCTCCTCTCTCAATATATTGAGGGCCTCCATGACGGATCCTGCGTTGAGGATCTTGGCATACGTGTCGTTGGCAGATTGCTGACCTCCTCTAGCTGATCTGCCGTCGATCTGGAAAACTCCATGATCAATGAAGTCTCCGTCTTTCTCCACGTAGGATTTGACATCTGATGAGCTCTTAGCTCCCTGAACGTTCGGATGGAAATGTGTTGATCTGGTTGGGGATATGAGGTCGAACAATCGTTCGTTTGTGCACTGTAATTTCCCTTCGAACTGGATGAGCACGTGGAGATGAGGTTCCCCATCTTCGTGTAATTCTCTGGCAACTCGGACGAACAATTTATTAACTGGCGTCTGTAACCCTAATAATTGAGATAGAGCTTCTTCTTTTGTTAAAGAGCACTTTGGATAAGTGAGGAAATAATTTTTAGAGTGGATTCTAAAACGACGTGGTAATGGCATTTTTGTAATTAAGGCGTGTACACCAATTGAGTTCTCTCTAAAACTCTATGGCAATTGGTGTATTGGGGTACAATATATACTAGAAGTCTCTATAGAACTATTAATCATAGTCACACACGTGGCGGCCATCCGTATAATATT |
| 2 | >Tomato severe rugose virus ToSRV:BR:G2:To:2009-2011 segment DNA-A, complete sequence  ACCGGATGGCCGCGCGATTTTTCACCCCTTTAGTTTCAATTAAAGTAAAGTGATTGTCTGTGGCCCAATCATATTGGGCCTGTCGAGCTTAGATATTTGTAACAACTTAAGGCCCAAGTTGTTAAACGGCTATAAATTGAACATACACTTTACTTTTGCTTTAATTCAAAATGCCTAAGCGTGATGCCCCATGGCGTTTAACGGCGGGAACTTCAAAGGTTTCCCGCTCTGTCAATTATTCTCCCCGTGCAGGATATGGACCCAAATATAACAAGGCCGCTGAGTGGGTGAACAGGCCCATGTACAGGAAGCCCAGGATCTACCGTACTTTGAGAGGCCCAGATGTTCCTAGAGGCTGTGAAGGGCCTTGTAAGGTTCAGTCTTACGAGTCTCGTCATGATGTTTCCCATGTCGGGAAGGTGATTTGTGTGTCTGACGTTACACGTGGTAACGGTATTACTCACCGTGTTGGTAAGCGTTTCTGCGTGAAGTCTGTATATATTTTAGGGAAGGTATGGATGGACGAGACCATCAAGTTGAAGAATCACACAAATAGTGTGATGTTCTGGTTGGTTAGAGATCGGAGACCTTATTCGTCACCTATGGATTTTGGCCAGGTGTTCAACATGTTTGACAACGAGCCTAGCACTGCAACTGTTAAGAACGATCTTCGGGATCGTTTTCAGGTCATGCACAAGTTTTATGCCAAGGTTACTGGTGGACAGTATGCCAGTAATGAGCAGGCATTAGTGAAGCGCTTTTGGAAGGTCAACAACAACGTAGTCTACAACCATCAGGAGGCAGGGAAATACGAGAATCATACTGAGAACGCCTTGCTATTGTATATGGCATGTACTCATGCCTCTAACCCCGTGTATGCTACATTGAAAATTCGGATCTATTTTTATGATTCGATTACTAATTAATAAAATTTAAATTTTATTGAATGATTTTCGAGTACAGCATTTACATATGATTTGTCTGTTGCGAAACGAACAGCTCTGATTACATTATTAATGGAAATAACGCCTAATCGATCTAGATACAATAAGACTAAATATTTAAATCTAGTTAAATATGTCGTCCCAGAAGCTGTCAGTGAAGTCGTCCATATCTGGAAGTTGAGGAAGCTCTTGTGGAGATGCAATGCTCTCCTCAGGTTGTGGTTGAACCGTATTTGGACGTGGTAGACTCTGCTCGCGGTGTACATTGGATCCTCCACTCTGTTTATCTTGAAATAGAGGGGATTTGATATCTCCCAAATATAAACGCCATTCTCTGCCTGACGTGCAGTGATGAGTTCCCCTGTGCGTGAATCCATGTCCTCTGCAGTCGATGTGTACGTAAATAGAGCACCCGCACTCTATATCAATTCGTCGTCTCCTGATTCCTCGTTTTTTAGCAGCTCTGTGTTGTACCTTGATAGAGGGGGGTGTTAAGGAAGACGAATTTCGCATTGTGCTTTGTCCAATTATTTAGACTTGCATTTTCTTCTTTGTCGAGGAAACATTTATAGCTGGCCCCCTCGCCAGGATTGCAAAGCACGATGCATGGGATACCACCTTTAATTTGAACTGGCTTTCCGTATTTGCAGTTTGATTGCCAATCCTTTTGGGCCCCAAGCAATTCTTTCCAGTGCTTTAACTTTAGATAGTGCGGTGCGATGTCATCAATGACGTTATATTCAACATGATTTGAATAAACCCTAGGATTGAAATCTAGGTGTCCACTCAAATAATTATGGGCCCCTAATGCACGTGCCCACATCGTCTTTCCCGTTCGAGAATCACCTTCAATGATGATACTAATAGGTCGTTCCGGCCGCGCAGCGGCACCTCTCCCAAAATAGTCATCTGCCCAAGCTTGCATCTCTCTCGGTACGCTAGTGAAAGAGGAGAGGGGAAACGTAGGGGCCCATGGCTCCGGAGCCCTTGTAAATATCCTATCTAAATTACTATTTAGATTGTGAAACTGAAATAAGAACTTTTCAGGCAGCTTCTCACGGATTATCTGCAGGGCGACGTCTTTGGAAGGTGCGTTCAAGGCTTCTGCGGCAGCGTCGTTAGCTGTCTGGCAACCGCCTCTAGCACTTCTTCCGTCGATTTGGAATTCCCCCCACTCGATAGTATCTCCGTCCTTGTCGACATAGGATTTGACGTCGGACGATGACTTAGCTCTCTGAATGTTCGGATGGAAATGTGTTGACCTTGTTGGGGAGACCAGGTCGAAGAATCGTTGATTTTGGCAGCAGTAGTTGCCCTCAAATTGAAGAAGCACGTGGAGATGAGGCTCCCCATTTTCATGGAGCTCTCTGCAAACCTTGATGAACTTCTTATTTGTAGGAGTGTTTAGGGTTTTTAATTGGGAAAGTGCTTCTTCTTTCGATAATGAGCATTTGGGATATGTGAGGAAATAATTCTTCGCTTTTATTTGAAAGCGCTTTGGAGCTGATGGCATATTTGTAAATATGACCCTTACTACCAAATGGTAGCTGCTCTAAAACTCATATGAATTGGTAGTTATGGTAGCTCTTATATAGTAGAAGTTCTTTTTAAGGAGATTGCTACACGTGGCGGCCATCCGTTATAATATT |
| 2 | >Tomato severe rugose virus ToSRV:BR:G2:To:2009-2011 segment DNA-B, complete sequence  ACCGGATGGCCGCGGGATTTTTCTTATCTGCTACGTGGCGAAATCGTGTACGTTGCCTCGCGCTTTCCATTTTAATTGAGCGCCTTTTTGAAGTCCGCGAAATGAGTTAATTGTCTTTTTGAAATCCGCTGTTTGTGCATCACCTTTAATTTGAATTAAAGGTTGGATAGTTCATATTGATCAATCATTTCGCTTGTTTATTTCCTGTCGTGGTGTAATTACAACCGTTCGTTAAAAATATAAGAAATTTACGACGTGGACTGTCTAAATTTCATCTACATAGTTAATTTGACAAATGAATGCATATTTAAACTCTGCTTTCGTGTGGGTTTACACCACGTCTATACATATTGTCCAGGTTATTTTGTATAAGTATAATTTTTATTTTGTCTTATCTTATTATCTGAACATGTATCCCATTAAGTATAGACGTGGAATGTTGTTTAATCATCGACGAGGTTACTCATCTAATCCCGTATTTAAGCGTTTACACGGAGCGAAACGAAGTGATTTCAAGCGTCGTTCGAGTAATCAGATTAAGAGTATGGATGAGACTAAAATGTCTGTTCAGCGGATTCATGAGAACCAGTTTGGCCCTGAATTTGTAATTGGCCACAATTCTGCCATATCCACATTCATTACATTCCCTACTCTTTGTAAGACTGTCCCTAACCGTTGTAGGTCATACATAAAGTTAAGACGTCTACGTTTTAAAGGAACAATCAAGATTGACCGTGTTCATGCTGAGGTGAATATGGACGGTACAAGTCCAATGATTGAAGGAGTCTTCTCTCTGGTTGTGGTCGTTGATCGCAAACCTCATTTGGGCTCATCTGGAACTCTGCATTCTTTTGATGAGATATTTGGTGCAAGGATTCATAGCCATGGTAACCTGGCAATAGTATCCTCTCTGAAAGAGCGTTTTTACATACGTCACGTTTGGAAGAAAGTAATATCTGTTGAGAAGGATACAACCATGGTTGATGTTGAAGGAAGTACTACTTTATCTAACAGGCGTTTTAATTGTTGGTCATCCTTTAAGGATATTGACCGTGAATCATGTAATGGTGTTTATGCAAACATAAGCAAGAACGCCCTGTTAGTTTATTACTGTTGGATGTCTGATAATGTGTCTAAGGCATCGACATTTGTATCATTTGACCTTGATTATGTTGGCTAAATAGTAAGTTTAATACGTTATTAGTAATAATGTTTAACTCGCTGTAATAATAAATACAAGTACTTGTTCAGTAATAATGTTCAGCTCGCAGTAATAACAAAAAAATTGTGTTTAACAATATAGAATTTATTTTAAAGATTTTGGCTGTGAAGGAGTACAGTTGCTGTTTATACATTCATGGGCCGCTGCTTTAACAAGTTCGTTTAATTGGGCCATTGACAGTGTTATGTTCGATTGGGCCCTTTGAGCACCAATTACGGACGCAGAATCACCTGGGTCTAATGTGCCTGTACCCAATCTATGTAATTGTCTGTATGGATGCATCGCGTTCTCTATATCTGAGCCCGCCTCTGATGGGCTTATGCCTACAGTACTTCTAACAGCCCATGACTCTCCGGGCATTATTTCAAGTGGGTTGTGAAGGCCCAATCTTGATATTGAGGCGGATCTGATCATCTTCCTTTCCCATTTGCCATAGCCTACGTGACTGAAATCAATGTCCTTATCTGTAAATTGTTTGGAAAGGATCTTCACCGTTGGGGCACGGAAAGGTATATCAACCGAATGTTTAGCCGTCGAGAGTTTCAGTCTCCCTTTGAATTTAGCGAAGTGGGTACTCTGGTGAACGTTCGTGTCACATACTCTGTAGTACAATTTCCATGGAATTGGGTCCTTGAGGGAGAAGAACGACGACGAAAAGTAGTGAAGATCTATGTTACATCTGATAGGAAAGGTCCAAGACGCCTGTAGTGATTCATTGTCCGTCATTCTTCTGTCATGAATTTCGACTATAACTGAACCTACGGCGTTAATTGGTACCTGTTGTCTGTATTCTATTACGCAATGGTCGATTTTCATACAGCTACGACTAAGCCTTGCGCTTAATTGAGACGCCGTTGAAGGGAATTGAAGCATAATCTCGGTTAGATCATGGGAAAGCTGATATTCATCACGCTTAGATTCTATATAATTAAATGCGTTGGGAGGATTTACAAGCTGAGATTCCATTATTGAAAATAGGGAGCGCAGCGACAATGTTTGAGGAAATTTAATAAGGGAAGATGATAATATTTTCGTCAACTGAATATATGACAAGAAAATTGTCTGTTGATCCAATTGGGAAATTGGAGATGATAATAACTAAAAAACGAGGAATCAGGAGACGACGAATTGAGAGGAGATATTGTCTATTTTAGATGTAATTGATAAATACCCCTCTATTTGCTCTTTAAATAGAATTTTTAAGGCAATGGCATATTTGTAAATATGACCCTTACTACCAAATGGTAGCTGCTCTAAAACTCATATGAATTGGTAGTTATGGTAGCTCTTATATAATAGAAGTTCTTTTTAAGGAGATTGCTACACGTGGCGGCCATCCGTTATAATATT |
| 3 | >Tomato mottle leaf curl virus ToMoLCV:BR:G3:To:2014-2016, complete sequence  ACCGGATGGCCGCGCGGGTTTTTTTGACCCGCTCCGTGACATATTTTTTGTCTTTTACTATGTGGTACAGTCAATAAATGACAAATATGACCGTCCAATCAGAAATGGTCCTCAAAGCCTAATTATTTAAAAATACTTGGTCACTAAGTTTGGTAAAGTTTATAAATGGTCCTTCCTCGTAATGTTATACCAACTTTAAGTCATAATGCCTAAGCGTGATGCCCCATGGCGCTCAATGGCGGGGACCTCAAAGGTTAGTCGGTCCGCCAATTTCTCCCCTCGTGGAGGAATCGGGTCGAAATCAGAGAAGGCCCAGGCCTGGGTTAACAGGCCCATGTTCAGGAAGCCCAGGATATATCGGATGTATAGAACCCCCGATGTACCAAAGGGATGCGAAGGCCCATGTAAGGTCCAATCCTTTGAGCAGCGTCACGATGTCCTTCATACGGGGAAGGTGATGTGTATTTCCGATGTCACTCGTGGTAACGGTATTACCCACCGCGTGGGTAAACGTTTTTGTGTCAAGTCTGTGTATATATTAGGCAAGGTCTGGATGGATGACAACATCAAGTTGAAGAACCACACGAATAGTGTGATGTTCTGGTTAGTTAGGGACAGGAGACCGTATGGCACTCCCATGGATTTTGGCCAGGTGTTCAACATGTTTGACAACGAGCCCAGTACTGCCACGGTGAAGAACGATCTCCGCGATCGTTACCAGGTCCTGCACCGGTTCTATGCCAAGGTTACAGGTGGACAATACGCCAGCAACGAGCAGGCTATTGTCAAGAGGTTCTGGAAGGTCAACAACCATGTGGTGTACAACCATCAGGAGGCTGGGAAATACGAGAACCATACGGAGAACGCTCTCTTATTGTATATGGCATGTACTCATGCCTCTAACCCTGTGTATGCTACTTTAAAAATTCGGATCTATTTTTATGATTCGATAACAAATTAATAAAGTTTAAATTTTATTGAATGATCTTCGAGTACATAATTTACATATGGTTTGTCTGTTGCGAATTGAACAGCTCTAATTACATTATTAATAGAAATGACTCCTAAACGATCTAAATACATATTAACTAAATCCCTAAACCTATTTAAATAAGTCGTCCCAGAAGCTGTCGTTGACGTCGTCCATATTTGGAAGTTGAGGAAGCACTTGTGGAGATCCAACGCTCTCCTCAGGTTGTGGTTGAACCGTATCTGTATGTGGTATATCCTTGTGTTCGTGTGTATCGGGTTCTCTACGTTTGTTATCTTGAAATATAGGGGATTTGGGACCTCCCAGATAAACGCGCCATTCTGTGCCTGATGTGCAGTGATGAGTTCCTCGGTGCGTGAATCCATTGCCGATGCAGTCGATGTGTTGGTATATTGTGCAGCCACAGTTTAGGTCTATGCGCTTGCGTCGAATGACCCTCTTCTTAGCTATCCTGTGTTGTGGTTTTATAGAGGGGGGTGTCGAGGAAGATGAATTTTGCATTGTGGAGTGTCCAGTTTTTGAGGGCTGTGTTTTCCTCTTTGTCGAGAAAATCTTTATAGCTGGCCCCTTCTCCTGGATTGCATAGCACGATTGAGGGTATCCCTCCTTTAATTTGAACTGGCTTTCCGTATTTGCAGTTTGATTGCCAGTCCTTTTGGGCCCCAATAAGCTCTTTCCAATGCTTCATCTTTAGATATTGCGGAGTGATGTCATCAATGACATTGTACTCCGCTTCGTTTGAGTAAACCCTATGATTGAAATCCAGGTGTCCACTCAAATAGTTATGTGGGCCTAATGCACGCGCCCACATTGTCTTCCCCGTCCTCGAATCACCCTCGATAATCAAACTAATAGGTCTCTCCGGCCGCGCAGCGGGATCCAGACCAAAATAATCATCAGCCCACCCTTGCATCTCCTCCGGAACCTCAGTAAAAGAGGAGAGTGGAAACGGAGGAACCCATGGTTCCGGAGCCTTTTTAAAGATGCGTTCTAAATTAGTATGGATGTTGTGATGTTGAAGGACGAAGTCCTTCGGTTGCTCCTCCCTCAATATATTGAGGGCCTCCATGACGGATCCTGCGTTGAGGATCTTGGCATACGTGTCGTTGGCAGATTGCTGACCTCCTCTAGCTGATCTGCCGTCGATTTGGAAAACTCCATGATCAATGAAGTCTCCGTCTTTCTCCACGTAGGATTTGACATCTGATGAGCTCTTAGCTCCCTGAACGTTCGGATGGAAATGTGTTGATCTGGTTGGGGATATGAGGTCGAACAATCGTTCGTTTGTGCACTGTAATTTCCCTTCGAACTGGATGAGCACGTGGAGATGAGGTTCCCCATCTTCGTGAAATTCTCTGGCAACTCGGACGAACAATTTATTAACTGGCGTCTGTAACCCTAATAATTGAGATAGAGCTTCTTCTTTTGTTAAAGAGCACTTTGGATAAGTGAGGAAATAATTTTTAGAGTTGATTCTAAAACGACGTGGTAATGGCATTTTTGTAATTAAGGCGTGTACACCAATTGAGTTCTCTCTAAAACTCTATGGCAATTGGTGTATTGGGGTACAATATATACTAGAACCCTCAATAGAACTTTCAATCTCGTTCACACACGTGGCGGCCATCCGATATAATATT |
| 3 | >Tomato severe rugose virus ToSRV:BR:G3:To:2014-2016 segment DNA-A, complete sequence  ACCGGATGGCCGCGCGATTTTTCACCCCTTTAGTTTCAATTAAAGTAAAGTGATTGTCTGTGGCCCAATCATATTGGGCCTGTCGAGCTTAGATATTTGTAACAACTTAAGGCCCAAGTTGTTAAACGGCTATAAATTGAACATACACTTTACTTTTGCTTTAATTCAAAATGCCTAAGCGTGATGCCCCATGGCGTTTAACGGCGGGAACTTCAAAGGTTTCCCGCTCTGTCAATTATTCTCCCCGTGCAGGATATGGACCCAAATATAACAAGGCCGCTGAGTGGGTGAACAGGCCCATGTACAGGAAGCCCAGGATCTACCGTACTTTGAGAGGCCCAGATGTTCCTAGAGGCTGTGAAGGGCCTTGTAAGGTTCAGTCTTACGAGTCTCGTCATGATGTTTCCCATATCGGGAAGGTGATTTGTGTGTCTGACGTTACACGTGGTAACGGTATTACTCACCGTGTTGGTAAGCGTTTCTGTGTGAAGTCTGTATATATTTTAGGGAAGGTATGGATGGACGAGACCATCAAGTTGAAGAATCACACAAATAGTGTGATGTTCTGGTTGGTTAGAGATCGGAGACCTTATTCGTCACCTATGGATTTTGGCCAGGTGTTCAACATGTTTGACAACGAGCCTAGCACTGCAACTGTTAAGAACGATCTTCGGGATCGTTTTCAGGTCATGCACAAGTTTTATGCCAAGGTTACTGGTGGACAGTATGCCAGTAATGAGCAGGCATTAGTGAAGCGCTTTTGGAAGGTCAACAACAACGTAGTCTACAACCATCAGGAGGCAGGGAAATACGAGAAACATACTGAGAACGCCTTGCTATTGTATATGGCATGTACTCATGCCTCTAACCCTGTGTATGCTACTTTAAAAATTCGGATCTATTTTTATGATTCGATAACAAATTAATAAAATTTAAATTTTATTGAATGATCTTCGAGTACATAATTTACATATGATTTGTCTGTTGCGAAACGAACAGCTCTGATTACATTATTAATGGAAATAACGCCTAATCGATCTAGATACAATAAGACTAAATATTTAAATCTACTTAAATATGTCGTCCCAGAAGCTGTCAGTGAAGTCGTCCATATCTGGAAGTTGAGGAAGCTCTTGTGGAGATGCAATGCTCTCCTCAGGTTGTGGTTGAACCGTATTTGGACGTGGTAGACTCTGCTCGCGGTGTACATTGGATCCTCCACTTTGTTTATCTTGAAATAGAGGGGATTTGATATCTCCCAAATATAGACGCCATTCTCTGCCTGACGTGCAGTGATGAGTTCCCCTGTGCGTGAATCCATGTCCTCTGCAGTCGATGTGTACGTAAATAGAGCACCCGCACTCTATATCAATTCGTCGTCTCCTGATTCCTCGTTTTTTAGCAGCTCTGTGTTGTACCTTGATAGAGGGGGGTGTTAAGGAAGACGAATTTCGCATTGTGCTTTGTCCAATTATTTAGACTTGCATTTTCTTCTTTGTCGAGGAAACATTTATAGCTGGCCCCCTCGCCAGGATTGCAAAGCACGATGCATGGGATACCACCTTTAATTTGAACTGGCTTTCCGTATTTGCAGTTTGATTGCCAATCCTTTTGGGCCCCAAGCAATTCTTTCCAGTGCTTTAACTTTAGATAGTGCGGTGCGATGTCATCAATGACGTTATATTCAACATGATTTGAATAAACCCTAGGATTGAAATCTAGGTGTCCACTCAAATAATTATGGGCCCCTAATGCACGTGCCCACATCGTCTTTCCCGTTCGAGAATCACCTTCAATGATGATACTAATAGGTCGTTCCGGCCGCGCAGCGGCACCTCTCCCAAAATAGTCATCTGCCCAAGCTTGCATCTCTCTCGGTACGCTAGTGAAAGAGGAGAGGGGAAACGTAGGGGCCCATGGCTCCGGAGCCCTTGTAAATATCCTATCTAAATTACTATTTAGATTGTGAAACTGAAATAAGAACTTTTCAGGCAGCTTCTCACGGATTATCTGCAGGGCGACGTCTTTGGAAGGTGCGTTCAAGGCTTCTGCGGCAGCGTCGTTAGCTGTCTGGCAACCGCCTCTAGCACTTCTTCCGTCGATTTGGAATTCCCCCCACTCGATAGTATCTCCGTCCTTGTCGACATAGGATTTGACGTCGGACGATGACTTAGCTCTCTGAATGTTCGGATGGAAATGTGTTGACCTTGTTGGGGAGACCAGGTCGAAGAATCGTTGATTTTGGCAGCAGTAGTTGCCCTCAAATTGAAGAAGCACGTGGAGATGAGGCTCCCCATTTTCATGGAGCTCTCTGCAAACCTTGATGAACTTCTTATTTGTAGGAGTGTTTAGGGTTTTTAATTGGGAAAGTGCTTCTTCTTTCGATAATGAGCATTTGGGATATGTGAGGAAATAATTCTTCGCTTTTATTTGAAAGCGCTTTGGAGCTGATGGCATATTTGTAAATATGACCCTTACTACCAAATGGTAGCTGCTCTAAAACTCATATGAATTGGTAGTTATGGTAGCTCTTATATAGTAGAAGTTCTTTTTAAGGAGATTGCCACACGTGGCGGCCATCCGTTATAATATT |
| 3 | >Tomato severe rugose virus ToSRV:BR:G3:To:2014-2016 segment DNA-B, complete sequence  ACCGGATGGCCGCGCGATTTTTCTTATCTGCTACGTGGCGAAATCGTGTACGTTGCCTCGCGCTTTCCATTTTAATTGAGCGCTTTTTTGAAGTCCGCGAAATGAGTTAATTGTCTTTTTGAAATCCGCTATTGTGAATCACCTTTAATTTGAATTAAAGGTTGGATAGTTCATATTGATCAATCATTTCGCTGGTTTATTTCCTGTCGTGGTGTAATTACAACCGTTCGTTAAAAATATAAGAAATTTACGACGTGGACTGTCTAAATTTCATCTACATAGTTAATTTGACAAATGAATGCATATTTAAACTCCGCTTTCGTGTGGGTTTACACCACGTCTATACATATTGTACAGGTTATTTTGTATAAGTATAATTTTTATTTTGTCTTATCTTATTATATGAACATGTATCCCATTAAGTATAGACGTGGAATGTTGTTTAATCATCGACGAGGTTACTCATCTAATCCCGTATTTAAGCGTTTACACGGAGCGAAACGAAGTGATTTCAAGCGTCGTTCGAGTAATCAGATTAAGAGCATGGATGAGACTAAAATGTCTGTTCAGCGGATTCATGAGAACCAGTTTGGCCCTGAATTTGTAATTGGCCACAATTCTGCCATATCCACATTCATTACATTCCCTACTCTTTGTAAGACTGTCCCGAACCGTTGTAGGTCATACATAAAGTTAAGACGACTACGTTTTAAAGGAACAATCAAGATTGACCGTGTTCATGCTGAGGTGAATATGGACGGTACAAGTCCAATGATTGAAGGAGTCTTCTCTCTGGTTGTAGTCGTTGATCGCAAACCTCATTTGGGTTCATCTGGAACTCTGCATTCTTTTGATGAGATATTTGGTGCAAGGATTCATAGCCATGGTAATTTGGCAATAGTATCCTCTCTGAAAGAGCGTTTTTACATACGTCACGTTTGGAAGAAAGTAATATCTGTTGAGAAGGATACAACCATGGTTGATGTTGAAGGAAGTACTATTTTATCTAACAGGCGTTTTAATTGTTGGTCATCCTTTAAGGATATTGACCGTGAATCATGTAATGGTGTTTATGCAAACATAAGCAAGAACGCCCTGTTAGTTTATTACTGTTGGATGTCTGATAATGTGTCTAAGGCATCGACATTTGTATCATTTGACCTTGATTATGTTGGCTAAATAGTAAGTGTAATACGTGATTAGTAATAATATTTAACTCGCTGTAATAATAAATACAAGTACTTGTTCAGTAATAATGTTCAACTCGCAGTAATAACAAAAAAATATGTTTAATAATATAGAATTTATTTTAAAGATTTTGGCTGTGAAGGAGTACAGTTGCTGTTTATACATTCATGGGCCGCTGCTTTAACAAGTTCGTTTAATTGGGCCATTGACAGTGTTATGTTCGATTGGGCCCTTTGAGCCCCAATTACGGACGCAGAATCACCTGGGTCTAATGTGCCTGTACCCAGTCTATGTAATTGTCTGTATGGATGCATCGCGTTCTCTATATCTGAGCCCGCCTCTGATGGGCTTAAGCCTACAGTACTTCTAACAGCCCATGACTCTCCGGGCATTATTTCAAGTGGGCTGTGAAGGCCCAATCTTGATATTGAGGCGGATCTAATCATCTTCCTTTCCCATTTGCCATAGCCTACGTGACTGAAATCAATGTCCTTTTCTGTAAATTGTTTTGAAAGGATCTTCACCGTTGGGGCACGGAAAGGTATATCAACCGAATGTTTAGCCGTCGAGAGTTTCAGTCTCCCTTTGAATTTAGCGAAGTGGGTACTCTGGTGAACGTTCGTGTCACATACTCTGTAGTACAATTTCCATGGAATTGGGTCCTTAAGGGAGAAGAACGACGACGAAAAGTAGTGAAGATCTATGTTACATCTGATAGGAAAGGTCCACGACGCCTGTAGTGATTCATTGTCCGTCATTCTTCTGTCATGAATTTCGACTATAACTGAACCTACGGCGTTAATTGGTACCTGTTGTCTGTATTCTATTACGCAATGGTCGATTTTCATACAGCTACGACTAAGCCTTGCGCTTAATTGAGACGCCGTTGAAGGGAATTGAAGCATTATCTCGGTTAGATCATGGGAAAGCTGATATTCATCACGCTTAGATTCTATATAATTAAATGCGTTGGGAGGATTCACAAGCTGAGATTCCATTATTGAAAATAGGGAGCGCAGCGACAATGTTTGAGGAAAGTTAATAAGGGAAGATGATAATATTTTCGTCAACTGAATATATGACAAGAAAATTGTCTGTTGATCCAATTGGGAAATTGGAGATGATAATAAGTAAAAAACGAGGAATCAGGAGACGACGAATTGAGAGGAGATATTGTCTATTTTAGGTGTAATTGATAAATACCCCTCTACTTGCTCTTTAAATAGAATTTTCAAGCCAAGGGCATATTTGTAAATATGACCCTTACTACCAAATGGTAGCTGCTCTAAAACTCATATGAATTGGTAGTTATGGTAGCTCTTATATAGTAGAAGTTCTTTTCAAGGAGATTGCCACACGTGGCGGCCATCCGTTATAATATT |
| 3 | >Tomato begomovirus-2 Bego2:BR:G3:To:2014-2016, complete sequence  ACCGGATGGCCGCGCGATTTTTTTTTAAGGCCCATTCATTTTGGGCCGCTTGGGCCGTTCTTTATTTGAATTAAAGTTAAGTCATTTATTCTGACCAATAACTTTAGTTCTGACGGGTTTAGTTAAATTTGAACTTGGCGACCAAGTTCTTTACTAAACTATAAATTTAATCTTGTATGGACCATGCTCTTTAATTCAAAATGCCTAAGCGTGATGCTCAATGGCGCCACATGGCAGGTACGTCAAAAATTAGCCGTTCGGCTAATTTCTCTCCTCGTGGGGGAAATGGGCCTAAATACAACAAGGCCGCTGAATGGGTTAACAGGCCTATGTATAGGAAGCCCAGGATATATCGAACTATACGAGGCCCTGATGTTCCACGGGGCTGTGAAGGCCCGTGCAAGGTTCAGTCTTATGAACAACGACATGATGTGTCACATGTTGGGAAGGTTATGTGTATATCTGATGTAACAAGAGGTAATGGTATTACCCATCGTGTAGGAAAACGTTTTTGTGTTAAGTCTGTTTATATTTTAGGTAAGATATGGATGGACGAGAATATCAAGGTGAAGAATCACACGAACAGTGTGATGTTTTGGTTAGTCAGAGATCGTAGACCGTATAGTTCTCCTATGGACTTTGGTCAAGTTTTTAATATGTTTGACAACGAGCCCAGTACTGCCACGGTGAAGAACGATCTCCGCGATCGTTACCAGGTCCTGCACCGGTTTTATGCCAAGGTTACAGGTGGACAATACGCCAGCAACGAGCAGGCTATTGTCAAGAGGTTCTGGAAGGTCAACAACCATGTGGTGTACAACCATCAGGAGGCTGGGAAATACGAGAACCATACGGAGAACGCCCTCTTATTGTATATGGCATGTACTCATGCCTCTAACCCTGTGTATGCTACTTTAAAAATTCGGATCTATTTTTATGATTCGATAACAAATTAATAAAGTTTAAATTTTATTGAATGATCTTCGAGTACATAATTTACATATGGTTTGTCTGTTGCGAATTGAACAGCTCTAATTACATTATTAATAGAAATGACTCCTAAACGATCTAAATACATATTAACTAAATCCCTAAACCTATTTAAATAAGTCGTCCCAGAAGCTGTCGTTGACGTCGTCCATATTTGGAAGTTGAGGAAGCTCTTGTGGAGATGCAATGCTCTCCTCAGGTTGTGGTTGAACCGTATTTGGACGTGGTAGACTCTGCTCGCGGTGTACATTGGATCCTCCACTTTGTTTATCTTGAAATAGAGGGGATTTGATATCTCCCAAATATAGACGCCATTCTCTGCCTGACGTGCAGTGATGAGTTCCCCTGTGCGTGAATCCATGTCCTCTGCAGTCGATGTGTACGTAAATAGAGCACCCGCACTCTATATCAATTCGTCGTCTCCTGATTCCTCGTTTTTTAGCAGCTCTGTGTTGTACCTTGATAGAGGGGGGTGTTAAGGAAGACGAATTTCGCATTGTGCTTTGTCCAATTATTTAGACTTGCATTTTCATCTTTGTCGAGGAAACATTTATAGCTGGCCCCCTCGCCAGGATTGCAAAGCACGATGCATGGGATACCACCTTTAATTTGAACTGGCTTTCCGTATTTGCAGTTTGATTGCCAATCCTTTTGGGCCCCAAGCAATTCTTTCCAGTGCTTTAACTTTAGATAGTGCGGTGCGATGTCATCAATGACGTTATATTCAACATGATTTGAATAAACCCTAGGATTGAAATCTAGGTGTCCACTCAAATAATTATGGGCCCCTAATGCACGTGCCCACATCGTCTTTCCCGTTCGAGAATCACCTTCAATGATGATACTAATAGGTCGTTCCGGCCGCGCAGCGGCACCTCTCCCAAAATAGTCATCTGCCCAAGCTTGCATCTCTCTCGGTACGCTAGTGAAAGAGGAGAGGGGAAACGTAGGGGCCCATGGCTCCGGAGCCCTTGTAAATATCCTATCTAAATTACTATTTAGATTGTGAAACTGAAATAAGAACTTTTCAGGCAGCTTCTCACGGATTATCTGCAGGGCGACGTCTTTTGAAGAAGCATTCAATGCTTCGGATGCCGCATCATTAGCTGTCTGTTGACCTCCTCTAGCAGATCTTCCGTCGACTTGAAAATGACCCCATTCGATGTAATCACCGTCCTTCTCGATGTAGGACTTGACATCAGAGGATGATTTTGCACTCTGTATATTGGCATGGGAGACGGAAGAGGTTGTTGGGTGTTTGAGGTCGAACACTCTGCAATTCGTGCACTGGAACTTGCCTTCGAATTGGATAAGAGCATGCAGATGTGGTTCCCCATTCTCGTGAATTTCTCTGCAAATTCTGATGTATTTCTTGTTCACCGGTGTTTGTAGGTTCTGAAGTTGTTCAATTGCACTCTCTTTTGTAATTGAACATTGTGGGTATGTGAGGAAATAATTCTTTGCGTTTATTTTAAAACGCTTTGGTGGTGGCATACTCGTAAATAAGAGGGTGTACCCCGATTGAGCTCTCGTTCAAAAGTCTCTATGAATCGGTGTAATGGTGCCAATATATAGTAAGAAGTTCTTTAAGGATAGTCACACACGTGGCGGCCATCCGTATAATATT |
