# Supplementary material for: A Temporal Diversity Analysis of Brazilian Begomoviruses in Tomato Reveals a Decrease in Species Richness between 2003 and 2016
Source: Front Plant Sci. 2020 Aug 6;11:1201. doi: 10.3389/fpls.2020.01201 (PMC7424291; doi:10.3389/fpls.2020.01201)
Supplement: Supplementary file 16 [file Table_5.docx]

**Supplementary Table 4.** BLAST analysis of DNA-A and DNA-B contigs assembled by Velvet from G1, G2 and G3 libraries.

| **Group** | | **Contig/** **Genomic component** | **Contig length (nt)** | **BLAST (accession)^1^** | **Percentage Identity (%)** | **E-value** |
| --- | --- | --- | --- | --- | --- | --- |
| 1 | 1 | SiMMV DNA-A | 581 | SiMMV DNA-A (JX415194) | 94.15 | 0 |
|  | 2 | ToCMoV DNA-A | 146 | ToCMoV DNA-A (KC706556) | 95.89 | 3e -56 |
|  | 3 | ToCMoV DNA-B | 152 | ToCMoV DNA-B (KC706567) | 86.18 | 6e-46 |
|  | 4 | TGVV DNA-A | 159 | TGVV DNA-A (JF803259) | 96.23 | 3e -69 |
|  | 5 | TGVV DNA-B | 223 | TGVV DNA-B (JF803265) | 97.76 | 9e-106 |
|  | 6 | ToMoLCV | 601 | ToMoLCV (KX896403) | 97.84 | 0.0 |
|  | 7 | ToRMV DNA-A | 223 | ToRMV DNA-A (AF291705) | 95.07 | 4e -99 |
|  | 8 | ToSRV DNA-A | 156 | ToSRV DNA-A (KY524458) | 92.31 | 6e -61 |
|  | 9 | ToSRV DNA-B | 224 | ToSRV DNA-B (KC706627) | 95.09 | 1e -99 |
|  | 10 | ToALCV | 213 | ToALCV (MG491197) | 94.37 | 2e-92 |
| 2 | 1 | SiMMV DNA-A | 1089 | SiMMV DNA-A (KU852503) | 96.51 | 0.0 |
|  | 2 | ToMoLCV | 208 | ToMoLCV (AY049211) | 97.12 | 2e -96 |
|  | 3 | ToSRV DNA-A | 142 | ToSRV DNA-A (AY029750) | 94.37 | 1e -56 |
|  | 4 | ToSRV DNA-B | 265 | ToSRV DNA-B (EF534708) | 96.23 | 2e -123 |
| 3 | 1 | BGMV DNA-A | 745 | BGMV DNA-A (MG334552) | 99.87 | 0.0 |
|  | 2 | ToMoLCV | 193 | ToMoLCV (JF803249) | 94.30 | 2e -82 |
|  | 3 | ToSRV DNA-A | 141 | ToSRV DNA-A (KX828624) | 99.29 | 7e -65 |
|  | 4 | ToSRV DNA-B | 142 | ToSRV DNA-B (EF534708) | 96.48 | 5e - 61 |

**^1^**Accession with the best matched sequence by Blast analysis.
