# Supplementary material for: A Temporal Diversity Analysis of Brazilian Begomoviruses in Tomato Reveals a Decrease in Species Richness between 2003 and 2016
Source: Front Plant Sci. 2020 Aug 6;11:1201. doi: 10.3389/fpls.2020.01201 (PMC7424291; doi:10.3389/fpls.2020.01201)
Supplement: Supplementary file 17 [file Table_6.docx]

| **Group** | | **Contig/** **Genomic component** | **Contig**  **length (nt)** | **BLAST (accession)^1^** | **Percentage Identity (%)** | **E-value** |
| --- | --- | --- | --- | --- | --- | --- |
| 1 | 1 | SiMMV DNA-A | 1807 | SiMMV DNA-A (AJ55745) | 94.19 | 0.0 |
|  | 2 | ToCMoV DNA-A | 588 | ToCMoV DNA-A (AF490004) | 89.97 | 0.0 |
|  | 3 | ToCMoV DNA-B | 903 | ToCMoV DNA-B (AF491306) | 87.94 | 0.0 |
|  | 4 | TGVV DNA-A | 1132 | TGVV DNA-A (JF803254) | 98.23 | 0.0 |
|  | 5 | TGVV DNA-B | 2230 | TGVV DNA-B (JF803265) | 98.79 | 0.0 |
|  | 6 | ToMoLCV | 2631 | ToMoLCV (KC706615) | 98.29 | 0.0 |
|  | 7 | ToRMV DNA-A | 827 | ToRMV DNA-A (AF291705) | 96.25 | 0.0 |
|  | 8 | ToSRV DNA-A | 1204 | ToSRV DNA-A (DQ207749) | 99.50 | 0.0 |
|  | 9 | ToSRV DNA-B | 619 | ToSRV DNA-B (EF534708) | 97.25 | 0.0 |
|  | 10 | ToALCV | 2228 | ToALCV (MG491197) | 96.01 | 0.0 |
| 2 | 1 | SiMMV DNA-A | 953 | SiMMV DNA-A (AJ557451) | 91.61 | 0.0 |
|  | 2 | ToMoLCV | 926 | ToMoLCV (KX896409) | 86.93 | 0.0 |
|  | 3 | ToSRV DNA-A | 630 | ToSRV DNA-A (DQ207749) | 98.42 | 0.0 |
|  | 4 | ToSRV DNA-B | 770 | ToSRV DNA-B (EF534708) | 96.88 | 0.0 |
| 3 | 1 | BGMV DNA-A | 923 | BGMV DNA-A (M88686) | 93.50 | 0.0 |
|  | 2 | ToMoLCV | 639 | ToMoLCV (KX896405) | 91.86 | 0.0 |
|  | 3 | ToSRV DNA-A | 585 | ToSRV DNA-A (JX415198) | 94.87 | 0.0 |
|  | 4 | ToSRV DNA-B | 406 | ToSRV DNA-B (EF534708) | 98.28 | 0.0 |

**Supplementary Table 5.** BLAST analysis of DNA-A and DNA-B contigs assembled by MEGAHIT from G1, G2 and G3 libraries.

**^1^**Accession with the best matched sequence by Blast analysis.
